# Crystal Structures, Optical Behavior, and Magnetic Properties in Hydrated Lanthanide Iron Sulfates

**DOI:** 10.1021/acs.inorgchem.5c04526

**Published:** 2026-01-22

**Authors:** Chloe Jones, Silu Huang, Tyler L. Spano, Eric A. Gabilondo, Mohammed Al-Fahdi, Kara Trim, Mary Douglas, Rongying Jin, Andrew Miskowiec, P. Shiv Halasyamani, Ming Hu, Jie Ling

**Affiliations:** † Department of Chemistry, 14843 University of Alabama in Huntsville, Huntsville, Alabama 35899, United States; ‡ SmartState Center for Experimental Nanoscale Physics, Department of Physics and Astronomy, 2629 University of South Carolina, Columbia, South Carolina 29208, United States; § Nuclear Nonproliferation Division, 6146 Oak Ridge National Laboratory, Oak Ridge, Tennessee 37820, United States; ∥ Department of Chemistry, 14743 University of Houston, Houston, Texas 77204, United States; ⊥ Department of Mechanical Engineering, University of South Carolina, Columbia, South Carolina 29201, United States

## Abstract

Single crystals of LnFe­(SO_4_)_3_(H_2_O)_2_ (Ln = La, Ce, Pr, Nd, Sm, Eu, Gd, Dy, Ho, Er, Tm; compounds **1–11**) and LnFe­(SO_4_)_3_(H_2_O) (Ln = Tm, Yb, Lu; compounds **12–14**) were synthesized under hydrothermal conditions. Single-crystal X-ray diffraction (SCXRD) analysis revealed that the dihydrated compounds (**1–11**) crystallize in centrosymmetric (CS) structures, with the lanthanide ions adopting eight-coordinate geometries. In contrast, the monohydrated compounds (**12–14**) exhibit noncentrosymmetric (NCS) structures, where the lanthanide ions are seven-coordinated. Vibrating sample magnetometry (VSM) confirmed that compounds **2**, **4**, and **7–11** are paramagnetic below 400 K, with compound **8** displaying the highest magnetic susceptibility. Compounds **1**, **5**, **6**, **13**, and **14** show a sharp increase in magnetic susceptibility at Néel temperatures (*T*_N_) of approximately 72, 76, 70, 58, and 56 K, respectively, indicating antiferromagnetic ordering. High-temperature magnetic susceptibility measurements further support the presence of antiferromagnetic transitions in these compounds. Second harmonic generation (SHG) measurements showed that the noncentrosymmetric Yb (compound **13**) and Lu (compound **14**) compounds exhibit SHG intensities of 0.3× and 1.6× that of potassium dihydrogen phosphate (KDP), respectively.

## Graphical abstract

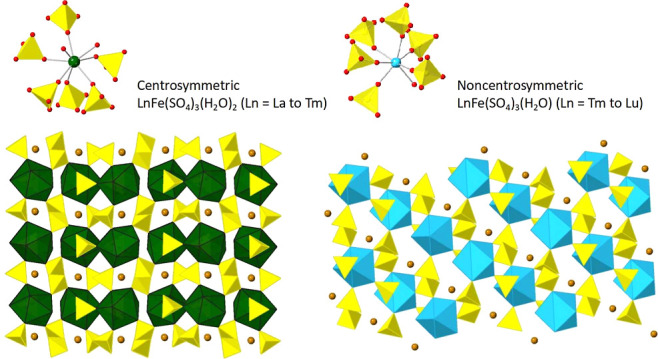

## Introduction

The integration of both 3d and 4f metal ions into a single compound has garnered considerable interest due to their potential in applications such as single-molecule magnets, gas storage, fluorescence, and optical sensing.^1–8^ The diverse coordination environments and electronic configurations of 3d and 4f metals contribute to their rich structural chemistry and varied electronic and magnetic behaviors. Typically, 3d metal ions adopt octahedral geometries with six-coordinated environments and exhibit a wide range of magnetic properties depending on their type and oxidation state. In contrast, trivalent 4f metal ions display coordination numbers varying from six to ten. Unlike 3d ions, the deeply buried 4f electrons in lanthanides experience weaker crystal-field effects. However, their strong spin–orbit coupling, large spin states, unquenched orbital angular momentum, and significant single-ion anisotropy can give rise to complex and fascinating magnetic phenomena in 3d/4f metal-based systems.

Most research on 3d/4f heterometallic compounds has primarily focused on systems with organic ligands, such as [Cu^II^LTb^III^(hfac)_2_]_2_ and Ni^II^Ln^II^ (Ln = Eu, Gd, Tb, Ho, Er, Y).^9,10^ In contrast, 3d/4f inorganic compounds remain relatively rare, with examples involving sulfate ligands being even scarcer. A family of lanthanide–copper hydroxysulfate compounds has been synthesized and their structurally and magnetically characterized.^11,12^ These include Ln_2_Cu­(SO_4_)_2_(OH)_4_ (Ln = Sm, Eu, Tb, Dy), Ln_2_Cu­(SO_4_)_2_(OH)_3_F·H_2_O (Ln = Gd, Ho, Yb), Ln_4_Cu­(SO_4_)_2_(OH)_10_, and LnCu­(SO_4_)­(OH)_3_ (Ln = Nd–Gd, Dy–Lu). In Ln_2_Cu­(SO_4_)_2_(OH)_4_, the Ln^3+^ ions form a distorted honeycomb lattice, with Cu^2+^ ions occupying the centers of the honeycomb framework. These isostructural compounds display distinct magnetic behaviors at low temperatures, likely due to variations in 3d–4f exchange interactions. In Ln_2_Cu­(SO_4_)_2_(OH)_3_F·H_2_O, the three-dimensional framework composed of [Ln_2_O_12_F_2_] units and [CuO_6_] chains exhibits paramagnetic behavior down to 2 K. The Weiss temperatures are close to zero, positive, or negative for Gd, Ho, and Yb analogues, respectively, suggesting different types of 3d/4f spin–orbital coupling effects. In the Ln_4_Cu­(SO_4_)_2_(OH)_10_ and LnCu­(SO_4_)­(OH)_3_ series, magnetic ordering varies with the lanthanide: GdCu­(SO_4_)­(OH)_3_ and HoCu­(SO_4_)­(OH)_3_ exhibit ferromagnetism, YCu­(SO_4_)­(OH)_3_ is antiferromagnetic, while Yb_4_Cu­(SO_4_)_2_(OH)_10_ shows paramagnetic behavior. Additionally, two series of mixed-ligand tellurite–sulfate 3*d*/4f compounds, Ln_2_Cu­(TeO_3_)_2_(SO_4_)_2_ (Ln = Y, Nd–Lu) and Ln_2_M­(TeO_3_)_2_(SO_4_)_2_ (Ln = Y, Nd–Lu; M = Co or Zn), have been reported.^13,14^ Structural variations across these series correlate with the decreasing ionic radii of the lanthanides, a result of lanthanide contraction. These compounds also exhibit intriguing magnetic properties arising from Co^2+^–Co^2+^, Ln^3+^–Ln^3+^, and Co^2+^–Ln^3+^ magnetic interactions.

Given the 3d^5^ electronic configuration of Fe^3+^, which imparts strong paramagnetic behavior, we systematically explored the synthesis of Ln^3+^/Fe^3+^ sulfate compounds and successfully discovered 14 new materials. Two distinct structural types were identified within this lanthanide iron sulfate hydrate series: centrosymmetric LnFe­(SO_4_)_3_(H_2_O)_2_ (Ln = La, Ce, Pr, Nd, Sm, Eu, Gd, Dy, Ho, Er, Tm) and noncentrosymmetric LnFe­(SO_4_)_3_(H_2_O) (Ln = Tm, Yb, Lu). These compounds were comprehensively characterized using single-crystal X-ray diffraction (SCXRD), powder X-ray diffraction (PXRD), infrared spectroscopy (IR), Raman spectroscopy, UV–vis–NIR spectroscopy, and thermogravimetric analysis (TGA). Magnetic properties were investigated using vibrating sample magnetometry (VSM). In addition, the second-harmonic generation (SHG) responses of the noncentrosymmetric LnFe­(SO_4_)_3_(H_2_O) (Ln = Yb, Lu) compounds were examined.

## Experimental Section

### Materials

Caution! Sulfuric acid is toxic and corrosive; it must be handled using the appropriate protective gear and training. The starting materials included: FeCl_3_·6H_2_O (Fisher Science Education, 99.9%), LaCl_3_·7H_2_O (Acros Organics, 99.99%), CeCl_3_·7H_2_O (Thermo Scientific, 99%), Pr_2_O_3_ (Thermo Scientific, 99%), Nd_2_O_3_ (Thermo Scientific, 99%), Sm_2_O_3_ (Stern Chemicals, 99.9%), Eu_2_O_3_ (Thermo Scientific, 99.9%), Gd_2_O_3_ (Thermo Scientific, 99.9%), Dy_2_O_3_ (Thermo Scientific, 99.9%), Ho_2_O_3_ (Thermo Scientific 99.9%), Er_2_O_3_ (Thermo Scientific, 99.9%)_,_ Tm_2_O_3_ (Thermo Scientific 99.9%), Yb_2_O_3_ (Thermo Scientific, 99.9%), Lu_2_O_3_ (Thermo Scientific, 99.9%), and sulfuric acid (0.5 M or 98%, Honeywell Fluka). Distilled and Millipore-filtered water with a resistance of 18.2 MΩ cm was used as the solvent.

### Syntheses

Single crystals of these new lanthanide iron sulfate compounds were synthesized via hydrothermal reactions. The selected reagents and solvent were sealed in a 23 mL PTFE-lined autoclave and heated at 230 °C for 4 days, followed by controlled cooling to room temperature at a rate of 9 °C per hour. The resulting products were washed with deionized water and ethanol, then dried under ambient conditions. The crystal habits of all analogues are comparable, and representative optical images of the crystals are shown in Figure 1.

**Figure 1:**
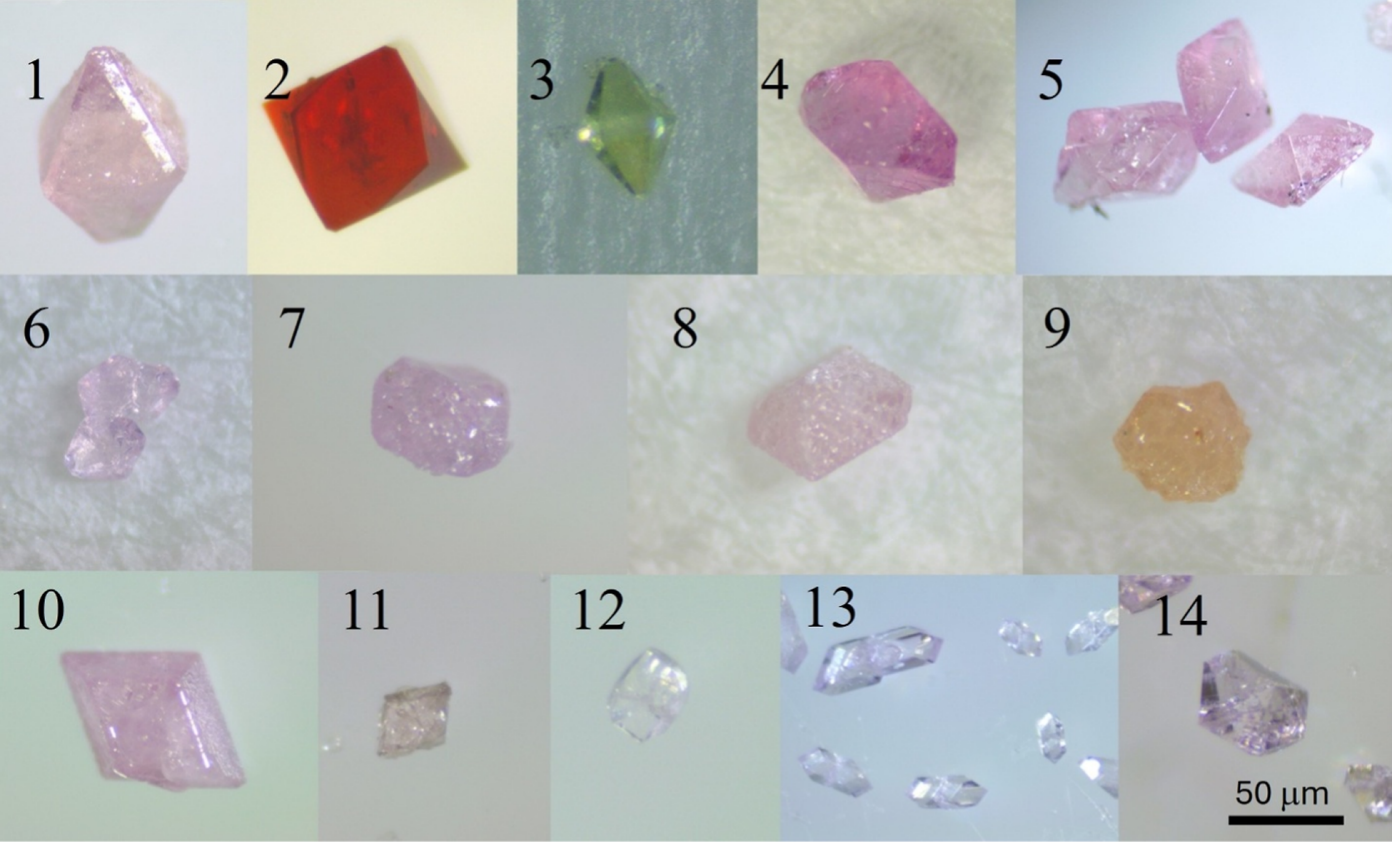
Optical images of crystals of LnFe­(SO_4_)_3_(H_2_O)_2_ (Ln = La (1), Ce (2), Pr (3), Nd (4), Sm (5), Eu (6), Gd (7), Dy (8), Ho (9), Er (10), Tm (11) and LnFe­(SO_4_)_3_(H_2_O) (Ln = Tm (12), Yb (13), Lu(14).

Compounds **1** (La) and **2** (Ce) were synthesized from reactions containing 0.25 mmol of LnCl_3_·7H_2_O (Ln = La and Ce), 0.25 mmol of FeCl_3_ ·6H_2_O, 0.5 mL of 98% H_2_SO_4_, and 1 mL of H_2_O. Pink and dark red crystals were obtained for La and Ce compounds, respectively.

Compounds **4** (Nd), **6** (Eu), **7** (Gd), **8** (Dy) and **9** (Ho) were synthesized from reactions containing 0.125 mmol of Ln_2_O_3_ (Ln = Nd, Eu, Gd, Dy, and Er), 0.25 mmol of FeCl_3_ ·6H_2_O, 1 mL of 0.5 M H_2_SO_4_, and 1.0 mL of H_2_O. Purple, light pink, pink, light pink, and light orange crystals were obtained for Nd, Eu, Gd, Dy, and Ho compounds, respectively.

Compounds **5** (Sm) and **12** (Tm) were synthesized from reactions containing 0.125 mmol of Ln_2_O_3_ (Ln = Sm and Tm), 0.25 mmol of FeCl_3_ ·6H_2_O, 1 mL of 98% H_2_SO_4_, and 0.5 mL of H_2_O. Pink and colorless crystals were obtained for Sm and Tm (compound **12**) compounds, respectively.

Compounds **3** (Pr), **10** (Er), **11** (Tm), **13** (Yb) and **14** (Lu) were synthesized from reactions containing 0.125 mmol of Ln_2_O_3_ (Ln = Pr, Ho, Tm, Yb, and Lu), 0.25 mmol of FeCl_3_ ·6H_2_O, 0.5 mL of 98% H_2_SO_4_, and 1.0 mL of H_2_O. Green, pink, colorless, light purple, and light purple crystals were obtained for Ho, Tm (compound **11**), Yb, and Lu compounds, respectively.

### Crystallographic Studies

Single crystals of all **14** compounds were selected using a polarized-light stereomicroscope and mounted on a MiTeGen loop for crystallographic studies. Crystallographic data of these compounds were collected on a Rigaku XtaLAB mini II single crystal X-ray diffractometer equipped with a graphite monochromator and Mo Ka radiation (λ = 0.71073 A) operating at 293 K. CrysAlisPro was used to perform such data reduction and multiscan absorption correction.^15^ Using Olex2, the structures were solved with the SHELXT structure solution program using intrinsic phasing and refined with the SHELXL refinement package using least squares minimization.^16,17^ All structural data were also checked for possible missing symmetry with the PLATON program and no higher symmetry was found.^18^ Data collection parameters and crystallographic information are listed in Table S1. The positional parameters, bond distances, and bond angles for Ln–O, Fe–O, and S–O can be found in Tables S2–S29 within the Supporting Information.

### Powder X-ray Diffraction

Powder X-ray diffraction (PXRD) patterns were collected using a Rigaku Miniflex 600 X-ray diffractometer with Cu–Kα radiation (λ = 1.54056 Å) over a 2θ range of 5°–60°, with a step size of 0.01°. The experimental patterns were compared to simulated PXRD patterns generated from single-crystal data using Mercury software.^19^

### Vibrational Spectroscopy

Infrared spectra were captured using a Thermo Nicolet iS50R FT-IR microspectrometer. The IR spectrum was collected with a diamond attenuated total reflectance (ATR) objective from 500 to 4000 cm^–1^ with a beam aperture of 100 μm. Final IR spectra consist of 64 total average scans. Raman spectra were collected using a Renishaw inVia micro-Raman spectrometer using a static scan with a 785 nm excitation wavelength, 5s exposure time, 20 accumulations of data, with 5% laser power (15 mW).

### UV–Vis–NIR

UV–vis NIR spectra were captured using an Agilent Cary 5000 UV–vis–NIR spectrophotometer equipped with a powder cell holder for diffuse reflectance measurements. Fine powder from each sample was individually loaded in the powder cell. The spectrum was taken from 200 to 2600 cm^–1^. The standard (100% transmittance) used was BaSO_4_. Zero percent transmittance was collected by removing the powder cell from the instrument. The Kubelka–Munk function was applied to transpose the reflectance spectrum to the absorbance data.^20,21^

### Thermal Analysis

Thermogravimetric analysis was obtained using a TA Discovery TGA 550 thermoanalyzer. 5–15 mg of sample was grinded into fine powder and placed in an alumina pan, then heated from 35 to 900 °C at a rate of 20 °C/min in open air. The resulting powders were analyzed by PXRD for phase identification post heating.

### SHG Activity Measurement

Powder SHG of compounds **13** and **14**, which adopt NCS crystal structures, was measured with a modified Kurtz–Perry system^22,23^ using a Nd/YAG solid-state laser at 1064 nm with KH_2_PO_4_ (KDP) in the particle size range of 90–125 μm serving as the reference.

### Computational Calculations

The structures were initially optimized with density functional theory (DFT) using the Vienna ab initio software (VASP).^24,25^ The optimization criteria in the structures were 1 × 10 −2 eV/Å and 1 × 10 −4 eV for forces and energy convergence, respectively. The exchange–correlation functional potential term in DFT formulation used the Perdew–Burke–Ernzerhof (PBE) generalized gradient approximation (GGA).^26^ The kinetic energy cutoff plane-wave basis of the wave function implemented in the optimization calculations was 520 eV for all the structures. Then, the electron localization function (ELF) was computed through self-consistent field (SCF) calculations with energy convergence criteria of 1×–4 eV for compound **14**. The kinetic energy cutoff plane-wave basis of the wave function applied in the SCF calculations was 520 eV for all the structures. Density of states (DOS) of compound **14** was also calculated using SCF calculations.

### Magnetic Property Measurement

The magnetic properties of the synthesized compounds were measured using vibrational sample magnetometry (VSM) in a Dynacool (Quantum Design). The magnetization (M) in both the zero-field-cooling (zfc) and field-cooling (fc) conditions were measured by applying 1 T DC magnetic field (H = 1 T). The magnetic susceptibility (χ) was calculated via χ = M/H.

### Bond-Valence Calculations

Bond valence sum (BVS) calculations were carried out for all 13 compounds by means of the equation of BVS = ∑exp­((*R*_0_–*R*)/b) in which *R* is the bond distance, *R*_0_ is the idealized tabulated bond length when the valence state is equal to one, and b is a bond valence parameter. The *R*_0_ values used for calculations are as follows: La^3+^-O^2–^, 2.148; Ce^3+^–O^2–^, 2.116; Pr^3+^–O^2–^, 2.098; Nd^3+^–O^2–^, 2.086; Sm^3+^–O^2–^, 2.063; Eu^3+^–O^2–^, 2.038; Gd^3+^–O^2–^, 2.031; Dy^3+^–O^2–^, 2.001; Ho^3+^–O^2–^, 2.025; Er^3+^–O^2–^, 1.988; Tm^3+^–O^2–^, 1.968; Yb^3+^–O^2–^, 1.965; Lu^3+^–O^2–^, 1.971; Fe^3+^–O^2–^, 1.759; and S^6+^–O^2–^, 1.624. A value of 0.37 was used for the b parameter in all calculations.^27,28^

## Results and Discussion

### Syntheses

All lanthanide iron sulfate compounds were synthesized under hydrothermal conditions, where initial H_2_SO_4_ concentration and water content proved critical for directing phase formation. As shown by the PXRD patterns (Figures S1–S14), most members of the series were obtained as single phases with typical yields of 30–65%. In contrast, the Pr (compound **3**) and two Tm compounds (compounds **11** and **12**) could not be prepared in phase-pure form. For Pr compound, the PXRD data consistently revealed minor but persistent unidentified secondary phases. For Tm, two hydration states—the dihydrate (11) and the monohydrate (12)—were reproducibly observed, with their relative formation strongly influenced by water content: the dihydrate dominated at higher water content (1.0 mL), whereas the monohydrate was favored under more limited hydration (0.5 mL). Despite extensive variation of synthetic parameters, including water content, acid concentration, reaction time, temperature, and reagent ratios, neither Tm phase could be isolated as a pure product, indicating that two hydration states are inherently competitive and coexist across a wide range of conditions.

### Crystal Structures

These **14** compounds represent the first reported examples of lanthanide iron sulfates. Single-crystal X-ray diffraction analysis revealed two distinct structural families: compounds **1–11** are dihydrated and crystallize in a centrosymmetric structure, whereas compounds **12–14** are monohydrated and adopt a noncentrosymmetric arrangement. A structural discontinuity is observed at thulium, which is unique in crystallizing in both structural types and space groups.

Compounds **1–11** crystallize in the centrosymmetric orthorhombic space group *Pbca* (no. 61) and feature a three-dimensional (3D) framework (Figure 2). The asymmetric unit contains one Ln, one Fe, three S, and 14 O atoms. Each Ln^3+^ (Ln = La to Tm) ion is coordinated by eight oxygen atoms, forming a LnO_8_ polyhedron with square antiprismatic geometry. Among these, six oxygen atoms originate from six corner-sharing sulfate ligands, while the remaining two oxygen atoms come from coordinated water molecules (Figure 2). The average Ln–O bond lengths range from 2.484 Å to 2.333 Å, decreasing linearly from La (1) to Tm (11), with the Ln–O­(water) bonds being slightly longer than the Ln–O­(sulfate) bonds. The Fe^3+^ ion adopts a slightly distorted octahedral FeO_6_ geometry, coordinated by six different sulfate groups in a corner-sharing arrangement (Figure 2). The Fe–O bond distances range from 1.964 (2) to 2.043(2) Å, and O–Fe–O bond angles vary from 79.62(9)° to 96.89(13)° (cis) and 167.66(9)° to 179.02(12)° (trans). Both LnO_8_ and FeO_6_ polyhedra are not directly connected, instead, they are linked via sulfate tetrahedra in a way that each sulfate group connect two LnO_8_ and two FeO_6_ polyhedra in a corner-sharing mode (Figure 2, and the shortest Ln^3+^···Ln^3+^, Ln^3+^···Fe^3+^, and Fe^3+^···Fe^3+^ distances are summarized in Table 1. Each sulfate group adopts a tetrahedral geometry with the S–O bond lengths falling in the range of 1.436(2) to 1.498(2) Å. The overall chemical formula for compounds **1–11** is LnFe­(SO_4_)_3_(H_2_O)_2_, and bond valence sum (BVS) calculations confirm the expected the oxidation states of Ln^3+^, Fe^3+^, S^6+^, and O^2–^ (Table S30).

**Figure 2:**
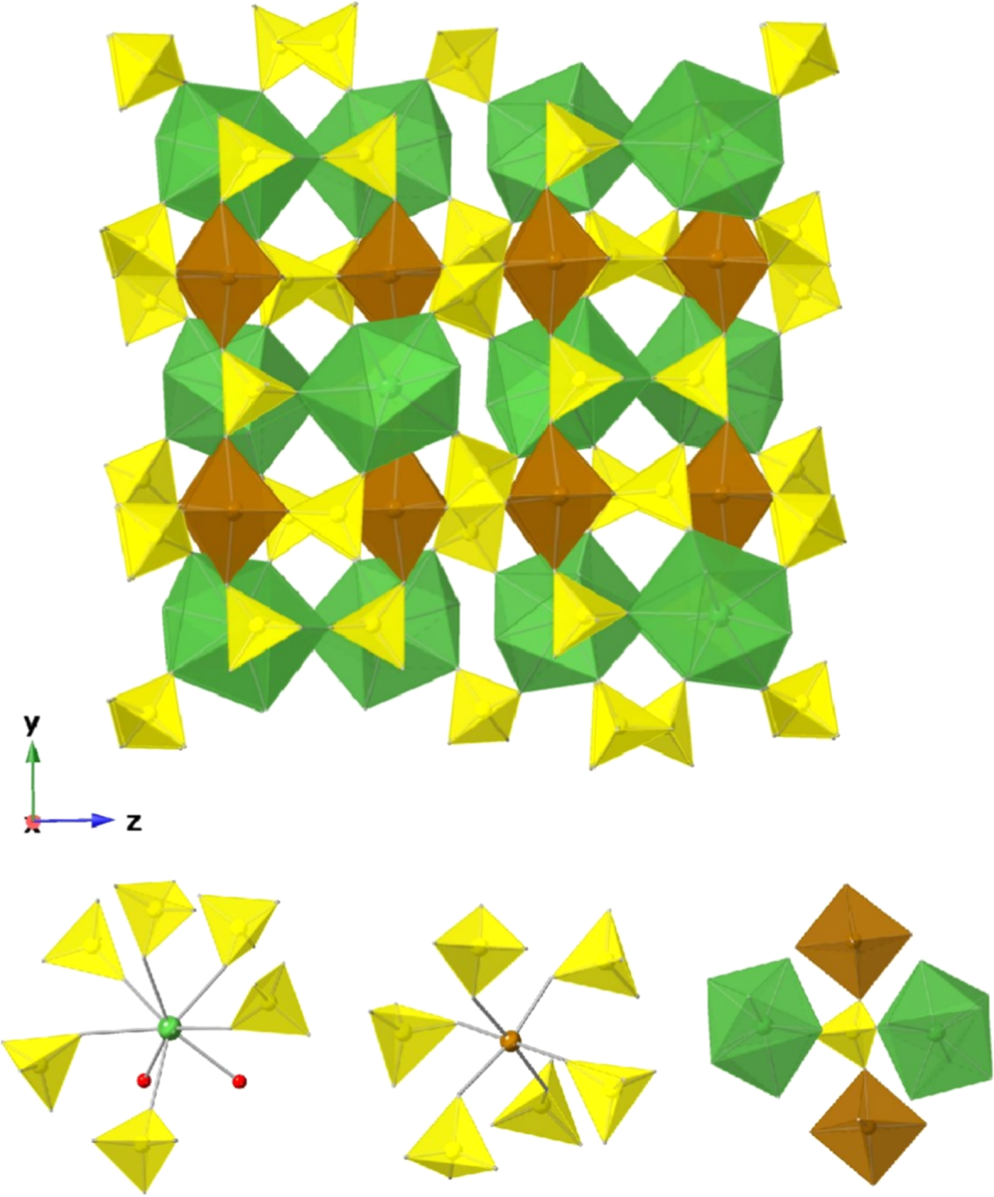
Three-dimensional framework structures of compounds **1–11** viewed along the *x* direction (top), composed by LnO_8_ (green), FeO_6_ (brown), and SO_4_ (yellow) polyhedra. The coordination environments of the lanthanide (left), iron (middle), and sulfate (right) units are shown at the bottom, where green, brown, and red spheres represent Ln, Fe, and O (water) atoms, respectively.

**Table 1:** Shortest Ln^3+^···Ln^3+^, Ln^3+^···Fe^3+^, and Fe^3+^···Fe^3+^ Distances in LnFe­(SO_4_)_3_(H_2_O)_2_ and LnFe­(SO_4_)_3_(H_2_O)

compound (Ln)	Ln^3+^···Ln^3+^ (Å)	Ln^3+^···Fe^3+^ (Å)	Fe^3+^···Fe^3+^ (Å)
**1** (La)	5.894(3)	5.060(4)	6.371(3)
**2** (Ce)	5.882(4)	5.032(3)	6.355(3)
**3** (Pr)	5.875(4)	5.016(4)	6.344(4)
**4** (Nd)	5.845(3)	4.970(3)	6.305(3)
**5** (Sm)	5.856(3)	4.986(3)	6.321(3)
**6** (Eu)	5.852(3)	4.972(3)	6.316(3)
**7** (Gd)	5.841(3)	4.958(3)	6.304(3)
**8** (Dy)	5.824(3)	4.939(3)	6.288(3)
**9** (Ho)	5.824(4)	4.931(4)	6.286(4)
**10** (Er)	5.813(4)	4.940(4)	6.283(4)
**11** (Tm)	5.805(3)	4.923(3)	6.270(3)
**12** (Tm)	6.241(3)	4.830(3)	6.241(3)
**13** (Yb)	6.240(3)	4.828(3)	6.240(3)
**14** (Lu)	6.243(4)	4.827(4)	6.243(3)

Compounds **12**–**14** also form a 3D framework (Figure 3) but differ from compounds **1–11** by crystallizing in the noncentrosymmetric trigonal space group *R*3*c* (no. 161). The asymmetric unit contains one Ln, one Fe, one S, and five O atoms. A key structural distinction lies in the coordination environment of the smaller Ln^3+^ ions, which are seven-coordinated by oxygen atoms, forming LnO_7_ polyhedra with a geometry best described as a capped octahedron. In their structures, each Ln^3+^ (Ln = Tm, Yb, or Lu) ion is coordinated by six oxygen atoms from six sulfate ligands and one from a water molecule (Figure 3), with average Ln–O bond distances of 2.253, 2.248, and 2.242 Å for Tm^3+^, Yb^3+^, and Lu^3+^, respectively. Notably, Ln–O­(water) bonds in the LnO_7_ polyhedra are all oriented at the same direction along the *z*-axis (red arrows, Figure 3), resulting in an overall noncentosymmetric structure. Fe^3+^ adopts an octahedral geometry (FeO_6_) with by corner-sharing a O atom with six sulfate groups (Figure 3). The Fe–O bond lengths range from 1.960(7) to 1.969(7) Å and O–Fe–O bond angles vary from 86.1(4)° to 91.9(3)° and 175.7(3)° to 176.1(3)° for cis- or trans-arrangements, respectively. As in compounds **1–11**, the LnO_7_ and FeO_6_ polyhedra are linked through sulfate groups, forming Ln–O–S–O–Ln, Ln–O–S–O–Fe, and Fe–O–S–O–Fe connections. Each sulfate tetrahedron bridges two LnO_7_ and two FeO_6_ polyhedra in a corner-sharing arrangement (Figure 3). The shortest Ln^3+^···Ln^3+^, Ln^3+^···Fe^3+^, and Fe^3+^···Fe^3+^ distances are listed in Table 1. The S–O bond lengths within sulfate groups fall in the range of 1.425(10)–1.480(8) Å. The overall chemical formula for compounds **12–14** is LnFe­(SO_4_)_3_(H_2_O), and BVS calculations confirm the expected oxidation states of Ln^3+^, Fe^3+^, S^6+^, and O^2–^ (Table S31).

**Figure 3:**
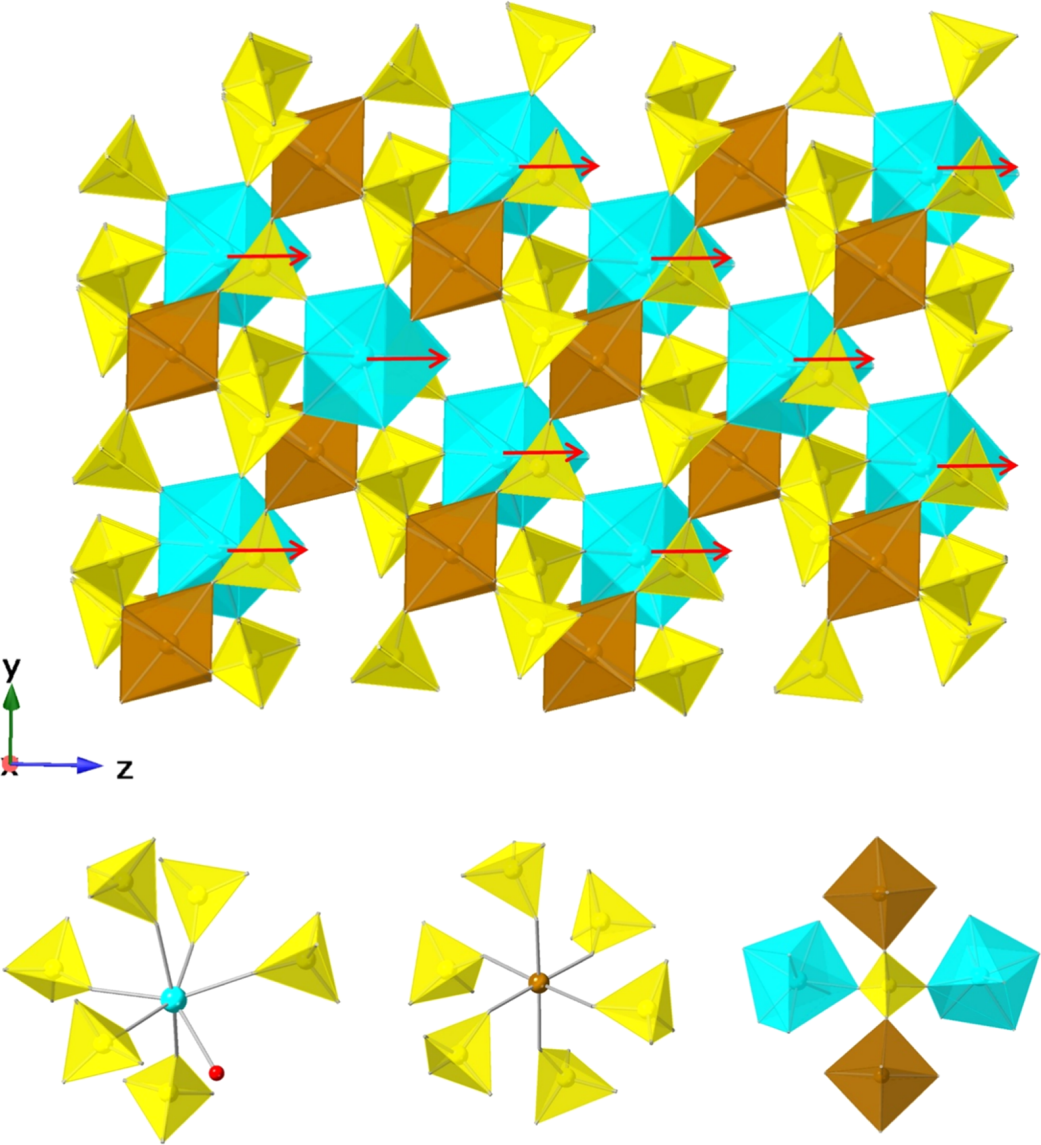
Three-dimensional framework structures of compounds **12–14** (top) viewed along the *x* direction (top), composed by LnO_7_ (cyan), FeO_6_ (brown), and SO_4_ (yellow) polyhedra. Red arrows indicate the polarity of the Ln–Ow bonds along *z* axis, and their constructive alignment gives rise to an overall noncentrosymmetric structure. The coordination environments of the lanthanide (left), iron (middle), and sulfate (right) units are shown at the bottom, where cyan, brown, and red spheres represent Ln, Fe, and O (water) atoms, respectively.

The transition from the dihydrate (*Pbca*) to the monohydrate (*R*3*c*) phase occurs at Tm, the only lanthanide capable of adopting both structures. This change is directly linked to the progressive lanthanide contraction and its impact on the local coordination environment (Figure 4).^29,30^ Across the La → Tm (*Pbca*) series, both the unit-cell volume and the average Ln–O bond length decrease smoothly (e.g., V: 2077.9 → 1981.5 Å^3^; Ln–O: 2.484 → 2.333 Å). By the time Tm is reached, the Ln–O coordination sphere has contracted to the point where incorporating two coordinated water molecules within the *Pbca* framework introduces substantial steric strain and distortion. This interpretation is supported by the bond-length trends: while Ln–O distances decrease steadily with increasing f-electron count, the Fe–O bond lengths vary only slightly (2.016 → 1.986 Å), indicating that the FeO_6_ octahedra remain comparatively rigid and cannot accommodate the contraction of the Ln-centered polyhedron. Upon transitioning to the monohydrate phases (Tm–Lu, *R*3*c*), a pronounced decrease in both unit-cell volume (e.g., Tm: 1981.5 → 1457.7 Å^3^) and Ln–O bond length (2.333 → 2.252 Å) is observed. This discontinuity demonstrates that removal of one coordinated water molecule relieves lattice strain and stabilizes a more compact, higher-symmetry rhombohedral framework. The coexistence of two hydration states at Tm therefore reflects a balance between lanthanide contraction, coordination-sphere energetics, and framework strain, placing Tm at a structural boundary where both phases are energetically accessible.

**Figure 4:**
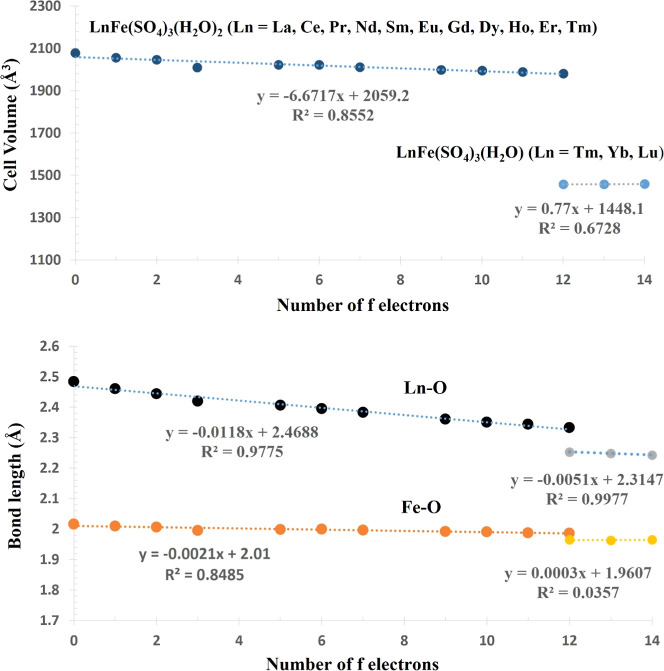
Unit-cell volumes (Å^3^) (top) and average Ln–O (black and gray) and Fe–O (orange and yellow) bond lengths (Å) (bottom) plotted as a function of the number of 4f electrons for compounds **1–14**. Dotted lines represent linear least-squares fits applied separately for LnFe­(SO_4_)_3_(H_2_O)_2_ (Ln = La, Ce, Pr, Nd, Sm, Eu, Gd, Dy, Ho, Er, Tm) (0–12 f electrons) and LnFe­(SO_4_)_3_(H_2_O) (Ln = Tm, Yb, Lu) (12–14 f electrons) series.

These trends are consistent with the shortest Ln^3+^···Ln^3+^, Ln^3+^···Fe^3+^, and Fe^3+^···Fe^3+^ distances observed across the series (Table 1). The Ln^3+^···Ln^3+^ and Ln^3+^···Fe^3+^ separations decrease steadily from compounds **1** to **11**, directly reflecting the contraction in ionic radius. Minor deviations near Sm and Eu—commonly encountered in the middle of the lanthanide series due to subtle electronic effects—do not interrupt the overall pattern of continuous structural compression around the Ln^3+^ centers. The separations increase in compounds **12–14** because these phases adopt a different hydration state and crystal structure; however, within each structure type, the characteristic lanthanide contraction remains clearly preserved. In contrast, the Fe^3+^···Fe^3+^ distances exhibit only slight variations, underscoring the rigidity of the FeO_6_ framework and its limited ability to respond to changes in lanthanide size.

### Vibrational Spectroscopy

Vibrational spectroscopy was employed to further confirm the presence of ligands in the compounds. In the IR spectra (Figure S15–S25), a broad absorption band around 3470 cm^–1^ corresponds to the O–H stretching vibrations of water molecules, while the band near 1600 cm^–1^ is attributed to the H–O–H bending mode.^31,32^ Strong absorption bands observed between 1000–1200 cm^–1^ and 600–700 cm^–1^ are assigned to the stretching and bending vibrations of S–O bonds in the sulfate groups, respectively.^31–33^ Complementary Raman spectra (Figure S37–S47) show two prominent peaks at approximately 1150 and 1075 cm^–1^, confirming the presence of sulfate through characteristic S–O stretching modes.

### UV–Vis–NIR Spectroscopy

Diffuse reflectance spectroscopy was used to investigate the optical absorption behavior of the compounds from the UV to the near-IR region. As shown in Figures S26–S37, all samples exhibit a pronounced drop in reflectance of approximately 90% near 420 nm, corresponding to a band gap of ∼3.0 eV as determined from Tauc analyses (inset figures). This feature is attributed to ligand-to-metal charge-transfer (LMCT) transitions involving Fe^3+^. Two weaker bands at around 520 and 750 nm are assigned to spin-forbidden Fe^3+^ d–d transitions, while broad features at approximately 1500, 2000, and 2500 nm arise from O–H stretching, bending, and combination modes associated with coordinated water molecules.^34,35^ The spectra of LaFe­(SO_4_)_3_(H_2_O)_2_ (1), GdFe­(SO_4_)_3_(H_2_O)_2_ (7), and LuFe­(SO_4_)_3_(H_2_O) (14) show similar overall profiles, consistent with their 4f electron configurations: empty (f^0^), half-filled (f^7^), and fully filled (f^14^), respectively. For compounds containing partially filled 4f shells, additional sharp absorption peaks appear. These arise from parity-forbidden but Laporte-allowed intraconfigurational f–f transitions of Ln^3+^ ions and are significantly sharper than the Fe^3+^ d–d features.^36,37^

### Thermal Analysis

The thermal behavior of the compounds was investigated using TGA, and the results are shown in Figures S48–S58. All samples display three distinct weight-loss steps between ambient temperature and 900 °C. The first step, occurring below ∼500 °C, corresponds to the loss of coordinated water (eq 1). The subsequent two steps between 500 to 900 °C are associated with the release of SO_3_ (eq 2), leaving LnFeO_3_ as the final solid residue, as confirmed by PXRD:

1 $$LnFe({\text{SO}}_{4}{)}_{3}(H_{2}{O)}_{x}\left(s\right)\to LnFe{\left({\text{SO}}_{4}\right)}_{3}\left(s\right)+{xH}_{2}O\left(g\right)\left(x=2\;\text{or}\;1\right)$$

2 $$LnFe{\left({\text{SO}}_{4}\right)}_{3}\left(s\right)\to {LnFeO}_{3}\left(s\right)+{3\text{SO}}_{3}\left(g\right)$$

The experimental weight-loss values agree closely with theoretical predictions. For instance, CeFe­(SO_4_)_3_(H_2_O)_2_ exhibits measured losses of 7.3% (H_2_O) and 45.7% (SO_3_), in excellent agreement with the calculated values of 6.9% and 46.1%, respectively.

A notable trend in the TGA data is the increasing thermal stability of the dehydrated LnFe­(SO_4_)_3_ compounds across the lanthanide series. The decomposition onset temperature rises from ∼550 °C for LaFe­(SO_4_)_3_ to ∼650 °C for LuFe­(SO_4_)_3_. This enhancement is attributed to the lanthanide contraction, where decreasing ionic radii result in shorter Ln–O bond distances and a more compact, thermally robust framework.

### Nonlinear Optical Properties

The nonlinear optical activities of compounds **13** (Yb) and **14** (Lu), both of which crystallize in noncentrosymmetric (NCS) structures, were evaluated by measuring their SHG responses. Relative to potassium dihydrogen phosphate (KDP), compound **13** exhibits an SHG intensity of 0.3×, whereas compound **14** shows a significantly stronger response of 1.6× (Figure 5). For context, other NCS lanthanide­(III) sulfate materials—such as CsLa­(SO_4_)_2_ and LiRE­(SO_4_)_2_ (RE = Y, Gd, Eu)—have reported SHG efficiencies of approximately 0.6× that of KDP and roughly 13× that of α-SiO_2_.^38,39^ Under comparable measurement conditions, α-SiO_2_ typically displays an SHG response of ∼0.2–0.3× that of KDP. Although compound **12** adopts the same structure as compounds **13** and **14**, its SHG activity could not be assessed due to the inability to obtain a phase-pure sample.

**Figure 5:**
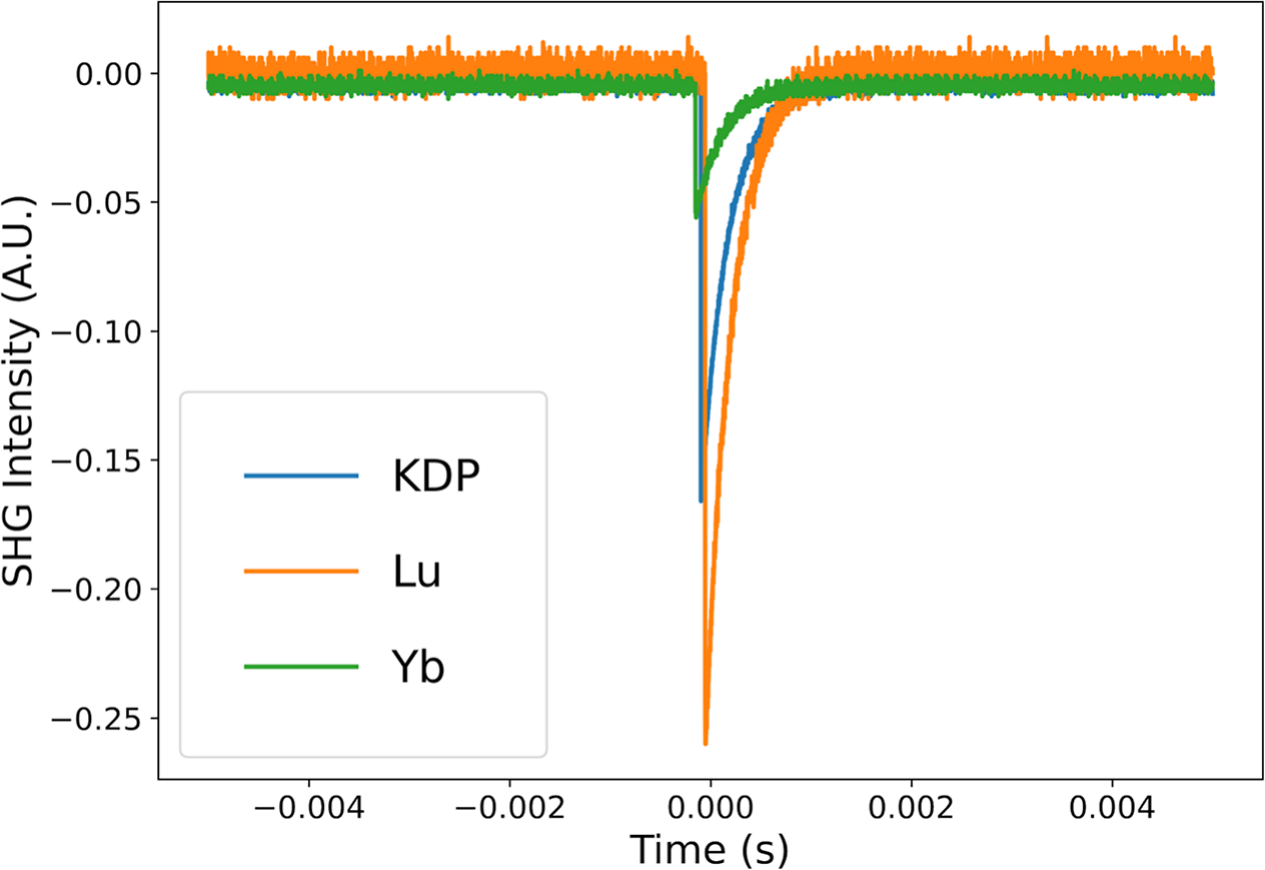
SHG intensity for compound **13** YbFe­(SO_4_)_3_(H_2_O) (green) and compound **14** LuFe­(SO_4_)_3_(H_2_O) (orange) compared to the standard KDP (blue).

To further clarify the origin of the SHG response and the differences between compounds **13** and **14**, the directions and magnitudes of the dipole moments of the LnO_7_, FeO_6_, and SO_4_ units were calculated (Table S32). In both compounds, the LnO_7_ polyhedron exhibits the largest dipole moment (1.80 D), oriented primarily along the *z*-axis, consistent with the alignment of the Ln–O­(water) bonds. The SO_4_ tetrahedra contribute comparably (1.75 D); however, their more random orientation reduces their effective contribution to the overall SHG response. While the FeO_6_ octahedra provide only minor contributions (0.41 D).

The difference in SHG intensity between the two compounds is further supported by UV–vis–NIR measurements. Compound **13** shows absorption near the laser excitation wavelength (1054 nm) due to intra4f (f–f) transitions of Yb^3+^ (Figure S35), which reduces the observed SHG signal. In contrast, compound **14** exhibits no absorption near ∼1000 nm, as Lu^3+^ has a fully filled 4f^14^ configuration that forbids f–f transitions (Figure S36). This explains the significantly stronger SHG response of compound **14** relative to compound **13**.

### Density of State and Electron Localization Function Map

The density of states (DOS) of compound **14** (Figure 6 top) reveals the electronic contributions from the constituent atoms and provides insight into the bonding interactions within the structure. At deep valence energies (around −20 eV), the DOS is dominated by O-2s states, consistent with strongly bound core-like oxygen orbitals. In the intermediate region (−12 to −6 eV), S-3s/3p and O-2p states contribute significantly, indicating strong S–O covalent bonding within the sulfate groups. Near the upper valence region (−5 to 0 eV), Fe-3d and O-2p states show substantial overlap, reflecting Fe–O hybridization and suggesting that Fe–O interactions play a key role in determining the material’s electronic behavior. The Lu-4f states appear as sharp, localized peaks slightly below the Fermi level, characteristic of the highly localized f-electron nature of Lu^3+^. Overall, the DOS profile highlights the dominant O-2p and Fe-3d interactions near the valence band edge, with sulfate and lutetium contributions mainly at lower energies.

**Figure 6:**
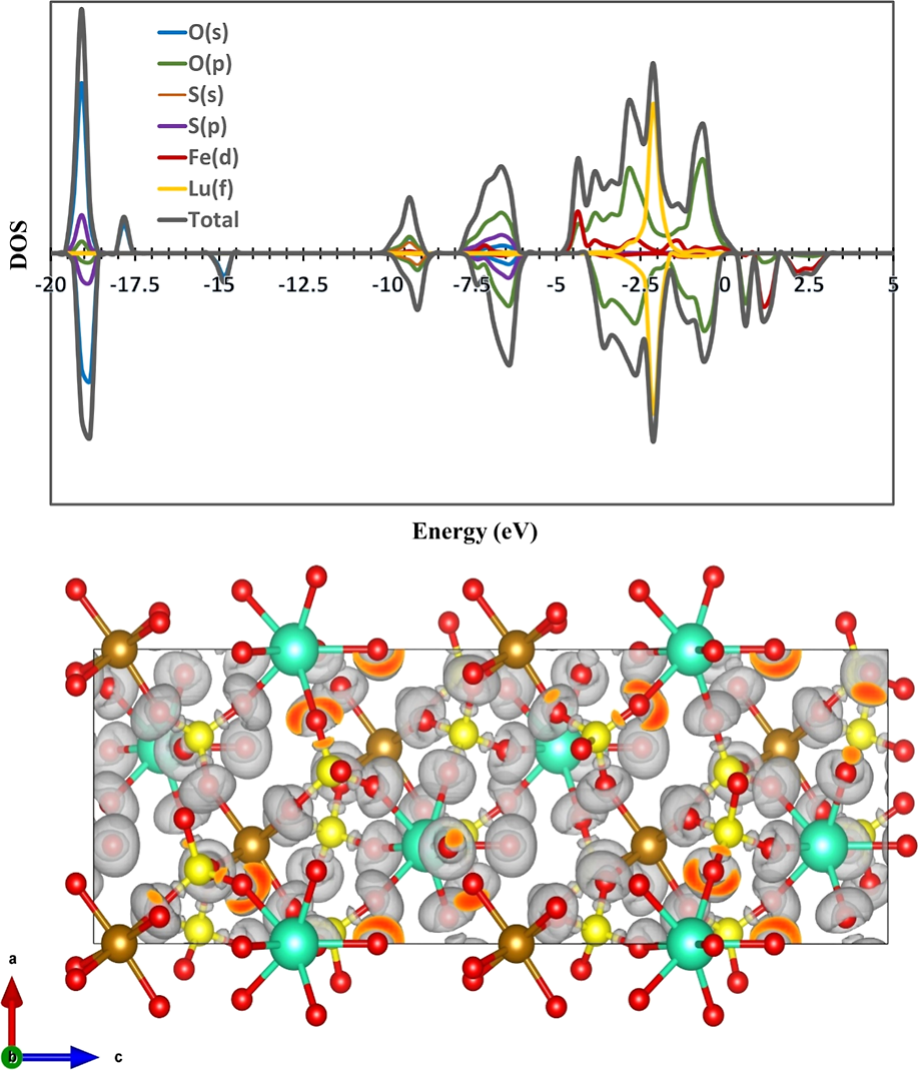
(Top) total and projected density of states (DOS) for LuFe­(SO_4_)_3_(H_2_O) (compound **14**), showing contributions from O (s, p), S (s, p), Fe (d), and Lu (f) orbitals. The Fermi level is set to 0 eV. (Bottom) real-space charge density distribution corresponding to states near the Fermi level, illustrating the spatial localization and orbital contributions within the crystal structure. Cyan, brown, yellow, and red spheres represent Lu, Fe, S, and O atoms, respectively.

The electron localization function (ELF) map of compound **14** provides a visual representation of electron localization with the crystal structure (Figure 6 bottom). The isosurfaces, shown in gradients of gray and orange-red, identify regions of high electron localization. These high ELF regions are primarily observed around O atoms, indicating strong localization of electron density associated with lone pairs and bonding interactions, particularly in Fe–O and S–O environments, suggesting covalent character in these bonds. In contrast, the ELF densities near Lu atoms are minimal, consistent with their role as electropositive, closed-shell cations that do not participate in significant covalent bonding. In addition, the ELF distribution exhibits asymmetric, noncentrosymmetric features. The electron density lobes around O atoms in both the sulfate groups and the coordinated water molecules are distinctly asymmetric and are not mirrored across the unit cell center. The LuO_7_ coordination environment shows a nonuniform ELF distribution, with Lu–O­(water) bonds are clearly aligned along the *c* axis. Visual inspection of the ELF map reveals a lack of inversion symmetry in the electron distribution, further supporting the noncentrosymmetric nature of the crystal structure of compound **14**.

### Magnetic Properties

The magnetic susceptibility (χ) for compounds **1–14** (except 3, 11, 12) was measured under a 1 T DC field in the temperature range of 2–400 K. The temperature dependence of χ and 1/χ were shown in Figure 7 and in Figures S59–S69, respectively. For all samples, the susceptibilities obtained under zero-field-cooled (ZFC) and field-cooled (FC) conditions are identical, indicating the absence of magnetic hysteresis. In general, χ increases monotonically as the temperature decreases. Among these materials, compound **8** exhibits the highest susceptibility over the entire temperature range, consistent with the large magnetic moment of Dy^3+^. Compounds **9** and **10** show similar magnitudes of χ, both slightly smaller than that of compound **8**.

**Figure 7:**
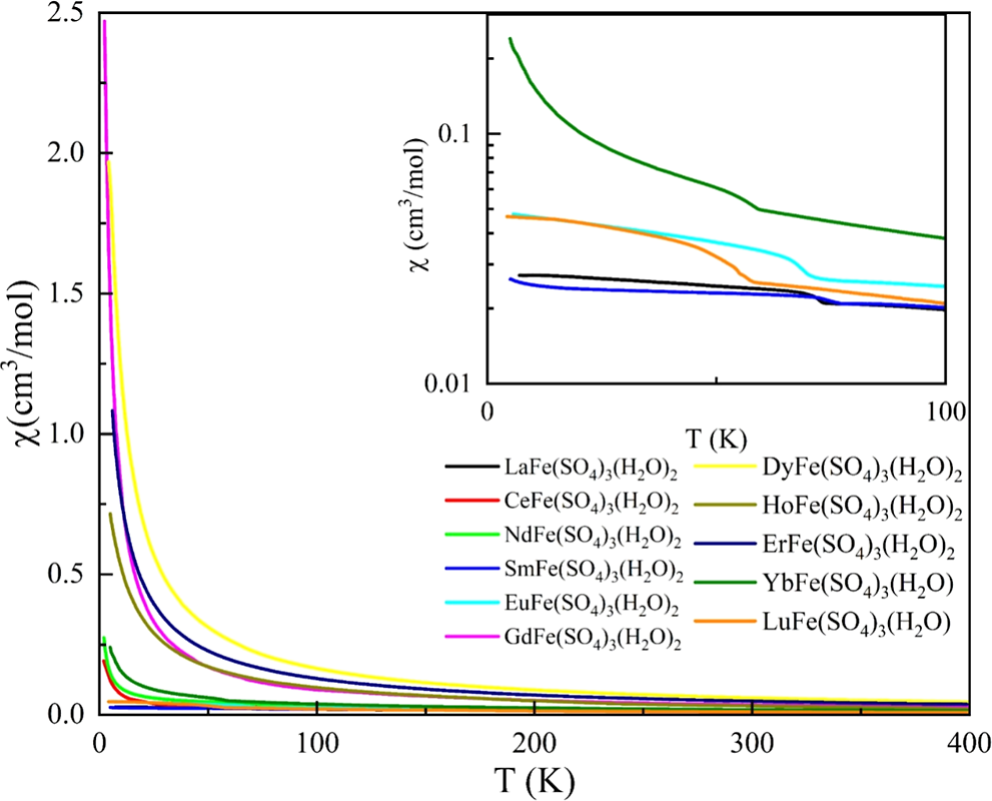
Temperature-dependent magnetic susceptibility, χ­(*T*) of compounds **1–14** (excluding **3**, **11**, and **12**) measured under an applied magnetic field of 1 T. For improved clarity, the χ­(*T*) curves of compounds **1**, **5**, **6**, **13**, and **14** are shown in the inset.

In the inset of Figure 7, we show χ­(*T*) between 2 and 100 K for compounds **1**, **5**, **6**, **13**, and **14**. Note that χ­(*T*) reveals a sudden jump at *T*_N_ ∼ 72 K for compound **1**, 76 K for compound **5**, 70 K for compound **6**, 58 K for compound **13**, and 56 K for compound **14**. To understand the nature of such a jump, we fit the magnetic susceptibility data using the Curie–Weiss (CW) formula χ = χ_0_ + C/(*T* – θ_CW_), where χ_0_ is a constant, C is the Curie constant, and θ_CW_ is the CW temperature. We find that θ_CW_ is −121 K for compound **1**, −164 K for compound **5**, −169 K for compound **6**, −76 K for compound **13**, and −93 K for compound **14**. The negative θ_CW_ implies the dominant antiferromagnetic interaction in these compounds. However, as χ_zfc_ = χ_fc_ at all temperatures below *T*_N_, there are only a portion of magnetic moments aligned antiferromagnetically. Through the relationship C = μ_eff_^2^/3*k*_B_ (*k*_B_ is the Boltzmann constant, we extract the effective moment μ_eff_ for each compound, which are listed in Table 2. Compared to the calculated values that also listed in Table 2, our experimentally obtained μ_eff_ values are very close. As listed in Table 2, the |θ_CW_| is much greater than *T*_N_ for all compounds, which may occur in either low-dimensional or frustrated magnetic systems. Through the structural analysis, all compounds are built up in a 3D framework, we suggest magnetic frustration in all studied compounds. This is especially true for compounds **7–10**, which are not ordered down to 2 K but have high effective magnetic moments (see Table 2).

**Table 2:** Magnetic Properties of LnFe­(SO_4_)_3_(H_2_O)_2_ and LnFe­(SO_4_)_3_(H_2_O)

compound	*T*_N_ (K)	*C* (cm^3^ K/mol)	θ_CW_ (K)	observed effective moment (μ_b_/F.U.)	calculated effective moment (μ_b_/F.U.)
**1** (La)	72	4.57	–121	6.05	5.92
**2** (Ce)	<2	5.22	–110	6.46	6.44
**4** (Nd)	<2	6.39	–102	7.15	6.94
**5** (Sm)	76	4.82	–164	6.21	5.98
**6** (Eu)	70	6.18	–169	7.03	6.83
**7** (Gd)	<2	11.9	–18	9.76	9.90
**8** (Dy)	<2	18.8	–14	12.27	12.20
**9** (Ho)	<2	17.6	–17	11.88	12.17
**10** (Er)	<2	15.7	–23	11.20	11.23
**13** (Yb)	58	6.78	–77	7.36	7.45
**14** (Lu)	56	4.03	–93	5.68	5.92

As shown in Figures 2 and 3, magnetic interactions can only go through superexchange type between Ln and Ln, Ln and Fe, and Fe and Fe, as LnO_8_ or LnO_7_ and FeO_6_ are separated by SO_4_. As listed in Table 1, the nearest distances between Ln–O-···-O-Ln, Ln–O-···–O–Fe, and Fe–O-···O–Fe decrease with increasing compound numbers, except for compounds **13–14** for Ln–O-···-O-Ln. The shortest distance is via Ln–O-···–O–Fe. Given that both *T*_N_ and |θ_CW_| do not follow the same trend as the distance of Ln–O-···–O–Fe, the bonding angle in the Ln–O-···–O–Fe pathway likely plays an important role. It is necessary to perform further experiments for quantifying the magnetic interaction such as neutron scattering to determine the exact spin configuration.

## Conclusion

Fourteen new lanthanide iron sulfate compounds have been synthesized under hydrothermal conditions. Because of lanthanide contraction, the early lanthanide members (Ln = La–Tm) crystallize in a centrosymmetric structure, LnFe­(SO_4_)_3_(H_2_O)_2_, while the late lanthanides (Ln = Tm–Lu) adopt a noncentrosymmetric structure, LnFe­(SO_4_)_3_(H_2_O). In these two structure types, the Ln^3+^ ions are coordinated by either eight or seven oxygen atoms—six from sulfate groups and two or one from coordinated water molecules for early and late lanthanides, respectively. Notably, Tm is capable of forming both structural types, acting as a transitional element within the series. In both structural families, the Ln–O bond lengths decrease progressively with increasing atomic number, consistent with the lanthanide contraction and correlating with enhanced thermal stability of the anhydrous phases observed in thermogravimetric (TG) analysis. Magnetic measurements using VSM revealed that compounds **2**, **3**, and **6–11** exhibit paramagnetic behavior below 400 K, with compound **7** showing the highest magnetic susceptibility. In contrast, compounds **1**, **4**, **5**, **12**, and **13** display sharp increases in susceptibility at Néel temperatures (*T*_N_) of approximately 72, 76, 70, 58, and 56 K, respectively, indicating the onset of antiferromagnetic ordering. High-temperature susceptibility data further support these antiferromagnetic transitions. Additionally, SHG measurements confirmed that the NCS Yb and Lu compounds exhibit SHG responses of 0.3× and 1.6× that of KDP, respectively, highlighting their potential for nonlinear optical applications.

## Supplementary Material



## Notes

The detailed crystallographic data, calculated bond-valence sums, powder XRD, FTIR, Raman, UV–vis–NIR, TGA, and magnetic results are included in the Supporting Information. Raw data that support the findings of this study are available from the corresponding author, upon reasonable request (PDF)
