# Supplementary material for: Crystal Structures, Optical Behavior, and Magnetic Properties in Hydrated Lanthanide Iron Sulfates
Source: Inorg Chem. 2026 Jan 22;65(4):2173–83. doi: 10.1021/acs.inorgchem.5c04526 (PMC12869489; doi:10.1021/acs.inorgchem.5c04526)
Supplement: Supplementary file 1 [file ic5c04526_si_001.pdf]

Supporting Information

Crystal Structures, Optical Behavior, and Magnetic Properties in Hydrated Lanthanide Iron Sulfates

Chloe Jones, [1] Silu Huang,[2] Tyler L. Spano,[3] Eric A. Gabilondo,[4] Mohammed Al-Fahdi,[5] Kara Trim,[1] Mary Douglas,[1] Rongying Jin,[2] Andrew Miskowiec,[3] P. Shiv Halasyamani,[4] Ming Hu,[5] Jie Ling[1*]

1. Department of Chemistry, University of Alabama in Huntsville, Huntsville, Alabama 35899, USA
2. SmartState Center for Experimental Nanoscale Physics, Department of Physics and Astronomy, University of South Carolina, Columbia, South Carolina 29208, USA
3. Nuclear Nonproliferation Division, Oak Ridge National Laboratory, Oak Ridge, Tennessee 37820, USA
4. Department of Chemistry, University of Houston, Houston, Texas 77204, USA
5. Department of Mechanical Engineering, University of South Carolina, Columbia, South Carolina, 29201, USA

Corresponding author and email: Jie Ling (jl0243@uah.edu)

Table of Contents

Table S1. Selected crystallographic data.

Table S2. Atomic coordinates and equivalent isotopic displacement parameters for compound **1**.

Table S3. Atomic coordinates and equivalent isotopic displacement parameters for compound **2**.

Table S4. Atomic coordinates and equivalent isotopic displacement parameters for compound **3**

Table S5. Atomic coordinates and equivalent isotopic displacement parameters for compound **4**

Table S6. Atomic coordinates and equivalent isotopic displacement parameters for compound **5**

Table S7. Atomic coordinates and equivalent isotopic displacement parameters for compound **6**.

Table S8. Atomic coordinates and equivalent isotopic displacement parameters for compound **7**.

Table S9. Atomic coordinates and equivalent isotopic displacement parameters for compound **8**.

Table S10. Atomic coordinates and equivalent isotopic displacement parameters for compound **9**.

Table S11. Atomic coordinates and equivalent isotopic displacement parameters for compound **10**.

Table S12. Atomic coordinates and equivalent isotopic displacement parameters for compound **11**.

Table S13. Atomic coordinates and equivalent isotopic displacement parameters for compound **12**.

Table S14. Atomic coordinates and equivalent isotopic displacement parameters for compound **13**.

Table S15. Atomic coordinates and equivalent isotopic displacement parameters for compound **14**.

Table S16. Selected bond distances (Å) and bond angles (deg) in compound **1.**

Table S17. Selected bond distances (Å) and bond angles (deg) in compound **2.**

Table S18. Selected bond distances (Å) and bond angles (deg) in compound **3.**

Table S19. Selected bond distances (Å) and bond angles (deg) in compound **4.**

Table S20. Selected bond distances (Å) and bond angles (deg) in compound **5.**

Table S21. Selected bond distances (Å) and bond angles (deg) in compound **6.**

Table S22. Selected bond distances (Å) and bond angles (deg) in compound **7.**

Table S23. Selected bond distances (Å) and bond angles (deg) in compound **8.**

Table S24. Selected bond distances (Å) and bond angles (deg) in compound **9.**

Table S25. Selected bond distances (Å) and bond angles (deg) in compound **10.**

Table S26. Selected bond distances (Å) and bond angles (deg) in compound **11.**

Table S27. Selected bond distances (Å) and bond angles (deg) in compound **12.**

Table S28. Selected bond distances (Å) and bond angles (deg) in compound **13.**

Table S29. Selected bond distances (Å) and bond angles (deg) in compound **14.**

Table S30. The calculated bond valence sums for La, Ce, Pr, Nd, Sm, Eu, Gd, Dy, Ho, Er, Tm, Fe, S, and O in compounds **1-11**.

Table S31. The calculated bond valence sums for Tm, Yb, Lu, Fe, S, and O in compounds **12**-**14**.

Table S32. Direction and magnitude of the dipole moments in compounds **13** and **14**.

Figure S1. Powder XRD result of compound **1.**

Figure S2. Powder XRD result of compound **2.**

Figure S3. Powder XRD result of compound **3.**

Figure S4. Powder XRD result of compound **4.**

Figure S5. Powder XRD result of compound **5.**

Figure S6. Powder XRD result of compound **6.**

Figure S7. Powder XRD result of compound **7.**

Figure S8. Powder XRD result of compound **8.**

Figure S9. Powder XRD result of compound **9.**

Figure S10. Powder XRD result of compound **10.**

Figure S11. Powder XRD result of compound **11.**

Figure S12. Powder XRD result of compound **12.**

Figure S13. Powder XRD result of compound **13.**

Figure S14. Powder XRD result of compound **14.**

Figure S15. Infrared spectra of compound **1.**

Figure S16. Infrared spectra of compound **2.**

Figure S17. Infrared spectra of compound **4.**

Figure S18. Infrared spectra of compound **5.**

Figure S19. Infrared spectra of compound **6.**

Figure S20. Infrared spectra of compound **7.**

Figure S21. Infrared spectra of compound **8.**

Figure S22. Infrared spectra of compound **9.**

Figure S23. Infrared spectra of compound **10.**

Figure S24. Infrared spectra of compound **13.**

Figure S25. Infrared spectra of compound **14.**

Figure S26. UV-Vis NIR spectra of compound **1.**

Figure S27. UV-Vis NIR spectra of compound **2.**

Figure S28. UV-Vis NIR spectra of compound **4.**

Figure S29. UV-Vis NIR spectra of compound **5.**

Figure S30. UV-Vis NIR spectra of compound **6.**

Figure S31. UV-Vis NIR spectra of compound **7.**

Figure S32. UV-Vis NIR spectra of compound **8.**

Figure S33. UV-Vis NIR spectra of compound **9.**

Figure S34. UV-Vis NIR spectra of compound **10.**

Figure S35. UV-Vis NIR spectra of compound **13.**

Figure S36. UV-Vis NIR spectra of compound **14.**

Figure S37. Raman spectra of compound **1.**

Figure S38. Raman spectra of compound **2.**

Figure S39. Raman spectra of compound **4.**

Figure S40. Raman spectra of compound **5.**

Figure S41. Raman spectra of compound **6.**

Figure S42. Raman spectra of compound **7.**

Figure S43. Raman spectra of compound **8.**

Figure S44. Raman spectra of compound **9.**

Figure S45. Raman spectra of compound **10.**

Figure S46. Raman spectra of compound **13.**

Figure S47. Raman spectra of compound **14.**

Figure S48. TGA curve of compound **1.**

Figure S49. TGA curve of compound **2.**

Figure S50. TGA curve of compound **4.**

Figure S51. TGA curve of compound **5.**

Figure S52. TGA curve of compound **6.**

Figure S53. TGA curve of compound **7.**

Figure S54. TGA curve of compound **8.**

Figure S55. TGA curve of compound **9.**

Figure S56. TGA curve of compound **10.**

Figure S57. TGA curve of compound **13.**

Figure S58. TGA curve of compound **14.**

Figure S59: Temperature dependence of inverse magnetic susceptibility ($1/\chi$) for compound **1.**

Figure S60: Temperature dependence of inverse magnetic susceptibility ($1/\chi$) for compound **2.**

Figure S61: Temperature dependence of inverse magnetic susceptibility ($1/\chi$) for compound **4.**

Figure S62: Temperature dependence of inverse magnetic susceptibility ($1/\chi$) for compound **5.**

Figure S63: Temperature dependence of inverse magnetic susceptibility ($1/\chi$) for compound **6.**

Figure S64: Temperature dependence of inverse magnetic susceptibility ($1/\chi$) for compound **7.**

Figure S65: Temperature dependence of inverse magnetic susceptibility ($1/\chi$) for compound **8.**

Figure S66: Temperature dependence of inverse magnetic susceptibility ($1/\chi$) for compound **9.**

Figure S67: Temperature dependence of inverse magnetic susceptibility ($1/\chi$) for compound **10.**

Figure S68: Temperature dependence of inverse magnetic susceptibility ($1/\chi$) for compound **13.**

Figure S69: Temperature dependence of inverse magnetic susceptibility ($1/\chi$) for compound **14.**

Table S1. Selected crystallographic data.

Rare Earth	La (1)	Ce (2)	Pr (3)	Nd (4)	Sm (5)
Empirical formula	$LaFeS_3O_{14}$	$CeFeS_3O_{14}$	$PrFeS_3O_{14}$	$NdFeS_3O_{14}$	$SmFeS_3O_{14}$
Formula weight	514.94	516.15	516.94	520.27	526.38
Crystal system	Orthorhombic	Orthorhombic	Orthorhombic	Orthorhombic	Orthorhombic
Space group	*Pbca*	*Pbca*	*Pbca*	*Pbca*	*Pbca*
a/Å	9.1464(4)	9.1048(2)	9.0921(2)	9.0278(10)	9.0549(2)
b/Å	12.7940(5)	12.7405(4)	12.7174(3)	12.6281(13)	12.664(3)
c/Å	17.7567(6)	17.7254(6)	17.6951(4)	17.6257(19)	17.6348(5)
α/°	90	90	90	90	90
β/°	90	90	90	90	90
γ/°	90	90	90	90	90

Volume/Å^3	2077.87(14)	2056.14(11)	2046.05(8)	2009.4(4)	2022.22(9)
Z	8	8	8	8	8
ρ $_{calc}$g/cm^3	3.292	3.335	3.356	3.440	3.458
μ/mm^{-1}	6.137	6.475	6.819	7.262	7.889
F(000)	1944	1952	1960	1968	1984
Radiation	Mo Kα (λ = 0.71073)	Mo Kα (λ = 0.71073)	Mo Kα (λ = 0.71073)	Mo Kα (λ = 0.71073)	Mo Kα (λ = 0.71073)
2Θ range for data collection/°	2.2930 to 30.4450	2.2820 to 30.5060	5.97 to 61.124	2.3040 to 30.6660	2.3100 to 30.5790
Index ranges	-11 ≤ h ≤ 10, -16 ≤ k ≤ 15, -22 ≤ l ≤ 21	-10 ≤ h ≤ 11, -16 ≤ k ≤ 15, -23 ≤ l ≤ 22	-12 ≤ h ≤ 12, -18 ≤ k ≤ 9, -20 ≤ l ≤ 25	-11 ≤ h ≤ 11, -14 ≤ k ≤ 16, -22 ≤ l ≤ 20	-11 ≤ h ≤ 11, -16 ≤ k ≤ 15, -21 ≤ l ≤ 22
Reflections collected	11667	10091	11462	11109	9689
Independent reflections	[R_{int} =, R_{sigma} =	[R_{int} =, R_{sigma} =	3062 [R_{int} = 0.0212, R_{sigma} = 0.0224]	[R_{int} =, R_{sigma} =	[R_{int} =, R_{sigma} =
Data/restraints/parameters	2350/0/172	2355/0/173	3062/0/173	2293/0/172	2319/0/172
Goodness-of-fit on F^2	1.061	1.047	1.059	1.060	1.038
Final R indexes [I>=2σ (I)]	R_1 =0.0209, wR_2 = 0.0530	R_1 =0.0276, wR_2 = 0.0599	R_1 = 0.0229, wR_2 = 0.0554	R_1 =0.0242, wR_2 = 0.0563	R_1 =0.0236, wR_2 = 0.0595
Final R indexes [all data]	R_1 =0.0289, wR_2 = 0.0507	R_1 =0.0413, wR_2 = 0.0562	R_1 = 0.0294, wR_2 = 0.0575	R_1 =0.0368, wR_2 = 0.0527	R_1 =0.0321, wR_2 = 0.0566

Largest diff. peak/hole /eÅ^{-3}	0.612/ -0.592	0.892/ -0.625	0.82/-0.80	0.959/ -0.706	0.729/ -0.744

Table 1 cont. Selected crystallographic data.

Rare Earth	Eu (6)	Gd (7)	Dy (8)	Ho (9)	Er (10)
Empirical formula	$EuFeS_3O_{14}$	$GdFeS_3O_{14}$	$DyFeS_3O_{14}$	$HoFeS_3O_{14}$	$ErFeS_3O_{14}$
Formula weight	527.99	533.28	538.53	540.96	543.29
Crystal system	Orthorhombic	Orthorhombic	Orthorhombic	Orthorhombic	Orthorhombic
Space group	*Pbca*	*Pbca*	*Pbca*	*Pbca*	*Pbca*
a/Å	9.0496(2)	9.0299(2)	9.0122(2)	9.0074(4)	9.0043(3)
b/Å	12.6765(3)	12.6485(4)	12.6216(3)	12.6101(5)	12.6129(5)
c/Å	17.6349(4)	17.6080(5)	17.5619(4)	17.5704(8)	17.5176(8)
α/°	90	90	90	90	90
β/°	90	90	90	90	90
γ/°	90	90	90	90	90
Volume/Å^3	2023.03(8)	2011.09(10)	1997.64(8)	1995.72(15)	1989.48(14)
Z	8	8	8	8	8
ρ_{calc}g/cm^3	3.467	3.523	3.581	3.601	3.628
μ/mm^{-1}	8.281	8.688	9.588	10.038	10.552
F(000)	1992.0	2000	2016	2024	2032
Radiation	Mo Kα (λ = 0.71073)	Mo Kα (λ = 0.71073)	Mo Kα (λ = 0.71073)	Mo Kα (λ = 0.71073)	Mo Kα (λ = 0.71073)
2Θ range for data collection/°	2.3060 to 30.4430	2.3080 to 30.5130	2.3200 to 30.4680	2.3020 to 30.4950	2.3250 to 30.3620

Index ranges	-12 ≤ h ≤ 12, -18≤ k ≤ 18 ,-25 ≤ l ≤ 25	-12 ≤ h ≤ 12, -18 ≤ k ≤ 18 , -24 ≤ l ≤ 24	-11 ≤ h ≤ 10, -16 ≤ k ≤ 16, -22 ≤ l ≤ 22	-9 ≤ h ≤ 12, -12 ≤ k ≤ 17, -24 ≤ l ≤ 23	-11 ≤ h ≤ 11, -16≤ k ≤ 13 , -22 ≤ l ≤ 22
Reflections collected		12519	11317	12513	9392
Independent reflections	3042[R_{int} =, R_{sigma} =	[R_{int} =, R_{sigma} =	[R_{int} =, R_{sigma} =	[R_{int} =, R_{sigma} =	[R_{int} = , R_{sigma} =
Data/restraints/parameters	3042/0/172	3005/0/102	2295/0/173	2968/0/107	2284/0/173
Goodness-of-fit on F^2	1.033	1.047	1.055	1.085	1.027
Final R indexes [I>=2σ (I)]	R_1 =0.0225, wR_2 = 0.0534	R_1 =0.0262, wR_2 = 0.0612	R_1 =0.0204, wR_2 = 0.0473	R_1 =0.0312, wR_2 = 0.0696	R_1 = 0.0223, wR_2 = 0.0465
Final R indexes [all data]	R_1 =0.0349, wR_2 = 0.0495	R_1 =0.0346, wR_2 = 0.0581	R_1 =0.0305, wR_2 = 0.0447	R_1 =0.0474, wR_2 = 0.0648	R_1 =0.0324, wR_2 = 0.0493
Largest diff. peak/hole /eÅ^{-3}	Eu (6)	1.420/ -1.020	0.859/ -0.560	1.280/ -1.110	0.672/ -0.834

Table 1 cont. Selected crystallographic data.

Rare Earth	Tm (11)	Tm (12)	Yb (13)	Lu (14)
Empirical formula	$TmFeS_3O_{14}$	$TmFeS_3O_{13}$	$YbFeS_3O_{13}$	$LuFeS_3O_{13}$
Formula weight	544.96	528.96	533.07	535.00
Crystal system	Orthorhombic	Trigonal	Trigonal	Trigonal
Space group	*Pbca*	*R*3*c*	*R*3*c*	*R*3*c*

a/Å	8.9875(3)	8.7542(7)	8.7565(4)	8.7636(2)
b/Å	12.5924(4)	8.7542(7)	8.7565(4)	8.7636(2)
c/Å	17.5087(6)	21.9634(19)	21.9497(9)	21.9397(5)
α/°	90	90	90	90
β/°	90	90	90	90
γ/°	90	120	120	120
Volume/$Å^3$	1981.53(11)	1457.7(3)	1457.54(15)	1459.24(7)
Z	8	6	6	6
$\rho_{calc}g/cm^3$	3.653	3.615	3.644	3.653
μ/mm^{-1}	11.079	11.282	11.777	12.298
F(000)	2040	1482	1488	1494
Radiation	Mo Kα (λ = 0.71073)	Mo Kα (λ = 0.71073)	Mo Kα (λ = 0.71073)	Mo Kα (λ = 0.71073)
2Θ range for data collection/°	2.3240 to 30.4760	2.7950 to 30.4950	3.2500 to 30.6390	3.2500 to 30.4880
Index ranges	-12 ≤ h ≤ 12, -17≤ k ≤ 18 , -24 ≤ l ≤ 23	-12 ≤ h ≤ 12, -12 ≤ k ≤ 12, -27 ≤ l ≤ 30	-12 ≤ h ≤ 12, -12 ≤ k ≤ 11, -31 ≤ l ≤ 31	-12 ≤ h ≤ 12, -12 ≤ k ≤ 12 , -31 ≤ l ≤ 29
Reflections collected	9652	6708	5045	6108
Independent reflections	[R_{int} =, R_{sigma} =	[R_{int} =, R_{sigma} =	[R_{int} =, R_{sigma} =	[R_{int} =, R_{sigma} =
Data/restraints /parameters	2932/0/173	962/1/35	959/1/34	974/1/35
Goodness-of-fit on F^2	1.049	1.062	1.099	1.066
Final R indexes [I>=2σ (I)]	R_1 =0.0269, wR_2 = 0.0736	R_1 =0.0218, wR_2 = 0.0515	R_1 =0.0264, wR_2 = 0.0626	R_1 = 0.0238, wR_2 = 0.0584

Final R indexes [all data]	R_1 =0.0421, wR_2 = 0.0690	R_1 =0.0220, wR_2 = 0.0514	R_1 =0.0290, wR_2 = 0.0611	R_1 = 0.0250, wR_2= 0.057 6
Largest diff. peak/hole \/eÅ^{-3}	1.590/-1.840	1.110/-1.120	1.216/ -1.179	1.341/ -1.250

Table S2. Atomic coordinates and equivalent isotopic displacement parameters for compound **1**.

Atom (site)	*x*	*y*	*z*	U_{eq}(Å^2)
La (1)	0.69391(2)	0.99798(2)	0.36746(2)	0.00877(7)
Fe (1)	0.27542(5)	0.73949(3)	0.37504(2)	0.00764(10)
S (1)	0.58587(9)	0.72991(6)	0.28716(4)	0.00833(16)
S (2)	0.21387(9)	0.49493(6)	0.33948(5)	0.00793(16)
S (3)	0.00637(9)	0.68862(6)	0.49207(4)	0.00829(16)
O (1)	0.6182(2)	0.73726(18)	0.20484(12)	0.0140(5)
O (2)	0.2651(3)	0.58284(17)	0.38755(13)	0.0133(5)
O (3)	-0.0662(3)	0.76015(17)	0.54674(13)	0.0133(5)
O (4)	0.4227(2)	0.73683(19)	0.29222(13)	0.0146(5)
O (5)	0.2348(3)	0.39842(17)	0.38470(13)	0.0136(5)
O (6)	0.3060(3)	0.49003(17)	0.27244(14)	0.0159(5)
O (7)	0.0603(3)	0.50684(17)	0.32076(14)	0.0149(5)
O (8)	0.1214(2)	0.75346(17)	0.45525(13)	0.0143(5)
O (9)	0.0734(3)	0.0734(3)	0.52993(13)	0.0181(6)
O (10)	0.6356(3)	0.63087(18)	0.31751(13)	0.0174(5)
O (11)	-0.0990(3)	0.65404(18)	0.43563(14)	0.0188(6)
O (12)	0.6529(3)	0.81651(18)	0.32691(13)	0.0186(6)
O (13)	0.9316(3)	0.9076(2)	0.34503(16)	0.0264(6)
O (14)	0.8652(3)	1.0395(2)	0.47521(15)	0.0249(6)

Table S3. Atomic coordinates and equivalent isotopic displacement parameters for compound **2**.

Atom (site)	*x*	*y*	*z*	U_{eq}(Å^2)
Ce (1)	0.30583(3)	0.99774(2)	0.63259(2)	0.00866(9)
Fe (1)	0.72405(6)	0.73971(5)	0.62491(4)	0.00753(14)
S (1)	0.41263(12)	0.73020(9)	0.71232(6)	0.0085(2)
S (2)	0.49310(13)	0.81247(8)	0.49209(6)	0.0088(2)
S (3)	0.78706(12)	0.49510(8)	0.66089(6)	0.0084(2)
O (1)	0.7661(4)	0.3982(2)	0.61569(18)	0.0133(7)
O (2)	0.3804(3)	0.7376(2)	0.79461(18)	0.0130(7)

O (3)	0.7346(4)	0.5827(2)	0.61272(17)	0.0129(7)
O (4)	0.5663(3)	0.7401(2)	0.54633(18)	0.0119(7)
O (5)	0.5760(3)	0.7369(3)	0.70744(18)	0.0146(7)
O (6)	0.3786(3)	0.7469(2)	0.45505(18)	0.0138(7)
O (7)	0.9418(3)	0.5069(2)	0.67940(18)	0.0143(7)
O (8)	0.6949(3)	0.4900(2)	0.72821(18)	0.0161(7)
O (9)	0.5980(4)	0.8482(3)	0.43540(19)	0.0182(8)
O (10)	0.4258(4)	0.9008(2)	0.53053(19)	0.0184(8)
O (11)	0.3624(4)	0.6309(2)	0.68159(18)	0.0167(8)
O (12)	0.3459(4)	0.8171(3)	0.67245(19)	0.0195(8)
O (13)	0.1360(4)	1.0381(3)	0.5253(2)	0.0236(8)
O (14)	0.0692(4)	0.9087(3)	0.6551(2)	0.0251(9)

Table S4. Atomic coordinates and equivalent isotopic displacement parameters for compound **3**.

Atom (site)	*x*	*y*	*z*	U_{eq}(Å^2)
Pr1	3059.3(2)	9976.2(2)	6327.4(2)	9.08(6)
Fe1	7234.1(5)	7399.5(3)	6249.7(2)	6.67(9)
S1	4107.3(8)	7305.2(6)	7118.2(4)	8.03(13)
S2	4926.8(8)	8133.7(6)	4919.3(4)	7.96(13)
S3	7877.6(8)	4953.1(5)	6612.2(4)	7.40(13)
O1	5663(2)	7409.7(17)	5459.7(12)	13.1(4)
O2	6965(2)	4896.9(17)	7288.5(12)	14.2(5)
O3	5746(2)	7371(2)	7069.3(12)	15.5(5)
O4	9426(2)	5076.5(17)	6792.3(13)	14.9(5)
O5	5982(3)	8498.3(18)	4356.3(13)	18.0(5)
O6	4235(3)	9014.7(17)	5306.4(13)	17.3(5)
O7	3601(3)	6314.9(18)	6808.0(13)	18.4(5)
O8	3439(3)	8176.7(18)	6720.6(13)	18.6(5)
O9	695(3)	9102(2)	6554.7(16)	26.5(6)
O10	1378(3)	10375(2)	5253.2(14)	23.9(6)
O11	3792(2)	7378.0(18)	7941.1(12)	13.6(4)
O12	7657(3)	3984.7(17)	6161.9(12)	13.2(4)
O13	3792(2)	7472.6(16)	4546.4(12)	13.2(4)
O14	7343(3)	5829.1(16)	6132.6(12)	12.7(4)

Table S5. Atomic coordinates and equivalent isotopic displacement parameters for compound **4**.

Atom (site)	*x*	*y*	*z*	U_{eq}(Å^2)
Nd (1)	0.69382(2)	0.99730(2)	0.63280(2)	0.00811(7)
Fe (1)	0.27766(6)	0.73992(4)	0.62511(3)	0.00679(12)
S (1)	0.59071(11)	0.73052(8)	0.71145(6)	0.0083(2)

S (2)	0.21144(11)	0.49534(8)	0.66161(6)	0.0076(2)
S (3)	0.50785(12)	0.81405(8)	0.49191(5)	0.0082(2)
O (1)	0.6226(3)	0.7380(2)	0.79350(16)	0.0131(6)
O (2)	0.2338(3)	0.3984(2)	0.61637(17)	0.0136(7)
O (3)	0.6211(3)	0.7476(2)	0.45453(16)	0.0136(6)
O (4)	0.4341(3)	0.7416(2)	0.54575(16)	0.0134(6)
O (5)	0.4270(3)	0.7361(2)	0.70667(16)	0.0157(6)
O (6)	0.2655(3)	0.5829(2)	0.61339(16)	0.0126(6)
O (7)	0.3021(3)	0.4893(2)	0.72924(17)	0.0146(7)
O (8)	0.0566(3)	0.5080(2)	0.67930(17)	0.0148(6)
O (9)	0.4032(3)	0.8516(2)	0.43547(17)	0.0164(7)
O (10)	0.5772(3)	0.9020(2)	0.53091(17)	0.0164(7)
O (11)	0.6424(4)	0.6315(2)	0.68047(17)	0.0178(7)
O (12)	0.9304(4)	0.9105(3)	0.6556(2)	0.0237(8)
O (13)	0.8606(4)	1.0363(3)	0.52613(19)	0.0219(7)
O (14)	0.6564(4)	0.8179(2)	0.67160(17)	0.0191(7)

Table S6. Atomic coordinates and equivalent isotopic displacement parameters for compound **5**.

Atom (site)	*x*	*y*	*z*	U_{eq}(Å^2)
Sm (1)	0.69383(2)	0.99709(2)	0.36711(2)	0.00738(7)
Fe (1)	0.27744(6)	0.73970(4)	0.37516(3)	0.00597(12)
S (1)	0.59251(10)	0.73087(8)	0.28939(5)	0.00720(19)
S (2)	0.21068(10)	0.49523(7)	0.33789(6)	0.0066(2)
S (3)	0.50838(11)	0.81534(8)	0.50826(5)	0.00713(19)
O (1)	0.2327(3)	0.3980(2)	0.38339(15)	0.0126(6)
O (2)	0.6244(3)	0.7376(2)	0.20702(15)	0.0125(6)
O (3)	0.6216(3)	0.7481(2)	0.54584(16)	0.0132(6)
O (4)	0.4285(3)	0.7353(2)	0.29355(15)	0.0145(6)
O (5)	0.4336(3)	0.7425(2)	0.45457(15)	0.0114(6)
O (6)	0.3010(3)	0.4899(2)	0.26997(16)	0.0135(6)
O (7)	0.0545(3)	0.5078(2)	0.32057(16)	0.0131(6)
O (8)	0.2646(3)	0.5826(2)	0.38673(15)	0.0111(6)
O (9)	0.5781(3)	0.9030(2)	0.46873(16)	0.0146(6)
O (10)	0.4039(3)	0.8532(2)	0.56488(17)	0.0163(6)
O (11)	0.9294(3)	0.9124(2)	0.34438(18)	0.0226(7)
O (12)	0.6569(3)	0.8194(2)	0.32921(17)	0.0172(7)
O (13)	0.8583(4)	1.0351(3)	0.47331(17)	0.0207(7)
O (14)	0.6459(3)	0.6322(2)	0.32082(16)	0.0171(7)

Table S7. Atomic coordinates and equivalent isotopic displacement parameters for compound **6**.

Atom (site)	*x*	*y*	*z*	U_{eq}(Å^2)
Eu (1)	0.30643(2)	0.00316(2)	0.63289(2)	0.00902(5)
Fe (1)	0.72090(5)	0.26038(3)	0.62507(2)	0.00773(9)
S (1)	0.40573(9)	0.26919(6)	0.71016(4)	0.00935(15)
S (2)	0.79023(8)	0.50474(6)	0.66255(4)	0.00846(14)
S (3)	0.49133(9)	0.18404(6)	0.49152(4)	0.00913(15)
O (1)	0.3789(3)	0.25167(18)	0.45382(14)	0.0159(5)
O (2)	0.5703(3)	0.2649(2)	0.70582(14)	0.0180(5)
O (3)	0.3740(3)	0.26183(19)	0.79262(13)	0.0151(5)
O (4)	0.7674(3)	0.60164(18)	0.61720(13)	0.0150(5)
O (5)	0.5666(3)	0.25734(18)	0.54476(13)	0.0146(5)
O (6)	0.7349(3)	0.41761(18)	0.61387(13)	0.0140(5)
O (7)	0.9467(3)	0.49189(19)	0.67956(14)	0.0157(5)
O (8)	0.7002(3)	0.51004(18)	0.73086(13)	0.0152(5)
O (9)	0.4204(3)	0.09694(19)	0.53145(14)	0.0174(5)
O (10)	0.5953(3)	0.14474(19)	0.43537(15)	0.0190(5)
O (11)	0.0715(3)	0.0871(2)	0.65560(17)	0.0270(6)
O (12)	0.1429(3)	-0.0343(2)	0.52671(15)	0.0240(6)
O (13)	0.3419(3)	0.18037(19)	0.67026(15)	0.0211(6)
O (14)	0.3521(3)	0.36733(19)	0.67833(15)	0.0206(6)

Table S8. Atomic coordinates and equivalent isotopic displacement parameters for compound **7**.

Atom (site)	*x*	*y*	*z*	U_{eq}(Å^2)
Gd (1)	0.30649(2)	0.49667(2)	0.36712(2)	0.00769(6)
Fe (1)	0.72056(6)	0.23953(4)	0.37492(3)	0.00663(10)
S (1)	0.40468(9)	0.23084(7)	0.29023(5)	0.00835(16)
S (2)	0.70936(9)	0.49515(7)	0.33727(5)	0.00737(15)
S (3)	0.49098(10)	0.31643(7)	0.50847(5)	0.00792(16)
O (1)	0.3785(3)	0.2487(2)	0.54625(15)	0.0139(5)
O (2)	0.5693(3)	0.2344(2)	0.29446(16)	0.0158(5)
O (3)	0.3732(3)	0.2378(2)	0.20785(15)	0.0135(5)
O (4)	0.5525(3)	0.5081(2)	0.32016(16)	0.0144(5)
O (5)	0.5661(3)	0.2430(2)	0.45542(15)	0.0135(5)
O (6)	0.7321(3)	0.3980(2)	0.38280(15)	0.0139(5)
O (7)	0.7649(3)	0.5822(2)	0.38604(15)	0.0123(5)
O (8)	0.4198(3)	0.4031(2)	0.46865(16)	0.0161(6)
O (9)	0.7993(3)	0.4900(2)	0.26870(16)	0.0146(6)
O (10)	0.3412(3)	0.3201(2)	0.33030(17)	0.0185(6)
O (11)	0.5954(3)	0.3559(2)	0.56494(16)	0.0178(6)
O (12)	0.0721(4)	0.4135(3)	0.34449(18)	0.0246(7)
O (13)	0.3505(3)	0.1325(2)	0.32216(17)	0.0188(6)
O (14)	0.1436(3)	0.5333(3)	0.47309(18)	0.0218(6)

Table S9. Atomic coordinates and equivalent isotopic displacement parameters for compound **8**.

Atom (site)	*x*	*y*	*z*	U_{eq}(Å^2)
Dy (1)	0.69331(2)	0.99628(2)	0.63285(2)	0.00810(7)
Fe (1)	0.28041(6)	0.73922(4)	0.62515(3)	0.00598(11)
S (1)	0.59777(11)	0.73092(8)	0.70881(5)	0.00851(19)
S (2)	0.20815(10)	0.49503(7)	0.66337(5)	0.00759(18)
S (3)	0.50908(11)	0.81757(8)	0.49117(5)	0.00815(19)
O (1)	0.6296(3)	0.7381(2)	0.79127(15)	0.0143(6)
O (2)	0.2311(3)	0.3981(2)	0.61780(15)	0.0143(6)
O (3)	0.6207(3)	0.7492(2)	0.45315(15)	0.0137(6)
O (4)	0.2648(3)	0.5820(2)	0.61454(14)	0.0122(6)
O (5)	0.4329(3)	0.7435(2)	0.54401(15)	0.0132(6)
O (6)	0.4329(3)	0.7337(2)	0.70497(15)	0.0174(6)
O (7)	0.4056(3)	0.8583(2)	0.43464(16)	0.0171(6)
O (8)	0.0510(3)	0.5081(2)	0.67985(15)	0.0145(6)
O (9)	0.8545(3)	1.0319(3)	0.52736(17)	0.0217(7)
O (10)	0.2972(3)	0.4896(2)	0.73229(15)	0.0147(6)
O (11)	0.5811(3)	0.9039(2)	0.53196(15)	0.0165(6)
O (12)	0.9279(4)	0.9146(3)	0.65511(18)	0.0249(7)
O (13)	0.6599(4)	0.8207(2)	0.66888(16)	0.0194(7)
O (14)	0.6529(4)	0.6330(2)	0.67650(16)	0.0202(7)

Table S10. Atomic coordinates and equivalent isotopic displacement parameters for compound **9**.

Atom (site)	*x*	*y*	*z*	U_{eq}(Å^2)
Ho (1)	0.30671(2)	0.99600(2)	0.36717(2)	0.00921(7)
Fe (1)	0.71911(7)	0.73901(5)	0.37480(4)	0.00740(13)
S (1)	0.40117(13)	0.73077(10)	0.29165(7)	0.0099(2)
S (2)	0.79242(13)	0.49474(9)	0.33641(7)	0.0088(2)
S (3)	0.49076(14)	0.81791(9)	0.50886(7)	0.0096(2)
O (1)	0.5658(4)	0.7339(3)	0.2953(2)	0.0189(8)
O (2)	0.3788(4)	0.7500(3)	0.5471(2)	0.0164(8)
O (3)	0.3694(4)	0.7379(3)	0.2089(2)	0.0156(7)
O (4)	0.5672(4)	0.7438(3)	0.4562(2)	0.0157(7)
O (5)	0.9504(4)	0.5083(3)	0.3204(2)	0.0156(7)
O (6)	0.7352(4)	0.5818(3)	0.38525(19)	0.0141(7)
O (7)	0.7688(4)	0.3979(3)	0.3822(2)	0.0154(8)
O (8)	0.7038(4)	0.4897(3)	0.2674(2)	0.0161(8)
O (9)	0.4188(4)	0.9044(3)	0.4681(2)	0.0176(8)
O (10)	0.5943(4)	0.8593(3)	0.5653(2)	0.0187(8)

O (11)	0.1465(5)	1.0310(3)	0.4722(2)	0.0231(9)
O (12)	0.3395(4)	0.8210(3)	0.3315(2)	0.0208(8)
O (13)	0.3455(4)	0.6333(3)	0.3242(2)	0.0197(8)
O (14)	0.0731(5)	0.9156(3)	0.3446(2)	0.0260(9)

Table S11. Atomic coordinates and equivalent isotopic displacement parameters for compound **10**.

Atom (site)	*x*	*y*	*z*	U_{eq}(Å^2)
Er (1)	0.30665(2)	0.49592(2)	0.36719(2)	0.00769(7)
Fe (1)	0.72057(6)	0.23911(4)	0.37516(3)	0.00622(12)
S (1)	0.40075(11)	0.23096(8)	0.29190(6)	0.0077(2)
S (2)	0.70700(11)	0.49474(8)	0.33626(6)	0.0067(2)
S (3)	0.99106(12)	0.18176(8)	0.49110(6)	0.0076(2)
O (1)	0.5660(3)	0.2325(3)	0.29560(17)	0.0173(7)
O (2)	0.3688(3)	0.2380(2)	0.20939(16)	0.0132(7)
O (3)	0.7303(4)	0.3973(2)	0.38173(17)	0.0133(7)
O (4)	0.7639(3)	0.5816(2)	0.38558(16)	0.0106(6)
O (5)	0.8789(3)	0.2506(2)	0.45304(17)	0.0144(7)
O (6)	1.0678(3)	0.2562(2)	0.54383(17)	0.0127(6)
O (7)	1.0942(3)	0.1403(2)	0.43438(18)	0.0163(7)
O (8)	0.9189(4)	0.0952(2)	0.53215(18)	0.0154(7)
O (9)	0.5493(3)	0.5081(2)	0.31998(18)	0.0138(7)
O (10)	0.0734(4)	0.4158(3)	0.3450(2)	0.0233(8)
O (11)	0.7963(3)	0.4896(2)	0.26730(17)	0.0138(7)
O (12)	0.1475(4)	0.5303(3)	0.4726(2)	0.0205(8)
O (13)	0.3442(4)	0.1331(2)	0.32443(18)	0.0178(7)
O (14)	0.3395(4)	0.3213(2)	0.33147(18)	0.0174(7)

Table S12. Atomic coordinates and equivalent isotopic displacement parameters for compound **11**.

Atom (site)	*x*	*y*	*z*	U_{eq}(Å^2)
Tm (1)	0.30682(2)	0.99566(2)	0.63280(2)	0.00846(7)
Fe (1)	0.71935(7)	0.73872(5)	0.62512(3)	0.00657(13)
S (1)	0.39959(12)	0.73088(8)	0.70766(6)	0.0089(2)
S (2)	0.79324(12)	0.49463(7)	0.66405(7)	0.0076(2)
S (3)	0.99113(11)	0.68128(8)	0.50902(6)	0.0085(2)
O (1)	0.5642(3)	0.7324(3)	0.70415(19)	0.0186(7)
O (2)	0.7359(4)	0.5811(2)	0.61509(17)	0.0131(7)
O (3)	0.8793(3)	0.7500(2)	0.54752(19)	0.0145(7)
O (4)	0.7693(4)	0.3976(3)	0.61796(18)	0.0154(7)
O (5)	0.9190(4)	0.5948(2)	0.46751(18)	0.0164(7)
O (6)	0.3668(3)	0.7378(2)	0.79045(17)	0.0143(7)

O (7)	1.0942(4)	0.6393(2)	0.56571(19)	0.0183(7)
O (8)	1.0683(3)	0.7554(2)	0.45691(18)	0.0140(7)
O (9)	0.7053(4)	0.4893(2)	0.7333(2)	0.0163(7)
O (10)	0.9521(4)	0.5075(2)	0.6799(2)	0.0151(7)
O (11)	0.0721(4)	0.9166(3)	0.6553(2)	0.0256(8)
O (12)	0.3390(4)	0.8218(2)	0.66795(19)	0.0195(8)
O (13)	0.1485(4)	1.0304(3)	0.5280(2)	0.0217(8)
O (14)	0.3416(4)	0.6337(3)	0.6750(2)	0.0208(8)

Table S13. Atomic coordinates and equivalent isotopic displacement parameters for compound **12**.

Atom (site)	*x*	*y*	*z*	U_{eq}(Å^2)
Tm (1)	0.333333	0.666667	0.38986(2)	0.00833(16)
Fe (1)	0.333333	0.666667	0.60977(7)	0.0065(3)
S (1)	0.6323(2)	0.71529(19)	0.51043(10)	0.0098(3)
O (1)	0.7966(9)	0.8817(8)	0.4985(3)	0.0242(12)
O (2)	0.5472(9)	0.7597(8)	0.5599(3)	0.0249(13)
O (3)	0.5236(9)	0.6574(8)	0.4565(3)	0.0258(12)
O (4)	0.6728(10)	0.5832(10)	0.5296(3)	0.0363(16)
O (5)	0.333333	0.666667	0.2851(9)	0.060(6)

Table S14. Atomic coordinates and equivalent isotopic displacement parameters for compound **13**.

Atom (site)	*x*	*y*	*z*	U_{eq}(Å^2)
Yb (1)	0.333333	0.666667	0.41438(2)	0.00816(14)
Fe (1)	1	1	0.46766(9)	0.0058(4)
S (1)	0.6318(3)	0.7138(3)	0.53473(12)	0.0098(3)
O (1)	0.7971(11)	0.8798(11)	0.5227(4)	0.0264(17)
O (2)	0.5474(12)	0.7588(10)	0.5845(4)	0.0260(16)
O (3)	0.5228(11)	0.6565(11)	0.4806(4)	0.0259(16)
O (4)	0.6719(12)	0.5824(13)	0.5539(4)	0.037(2)
O (5)	0.333333	0.666667	0.3096(10)	0.056(6)

Table S15. Atomic coordinates and equivalent isotopic displacement parameters for compound **14**.

Atom (site)	*x*	*y*	*z*	U_{eq}(Å^2)
Lu (1)	0.666667	0.333333	0.58575(2)	0.00836(13)
Fe (1)	1	1	0.53240(8)	0.0049(3)
S (1)	0.7123(2)	0.6311(2)	0.46579(11)	0.0105(3)
O (1)	0.8782(10)	0.7967(11)	0.4777(4)	0.0286(16)

O (2)	0.7582(10)	0.5474(11)	0.4160(4)	0.0282(16)
O (3)	0.6558(10)	0.5224(10)	0.5200(3)	0.0270(15)
O (4)	0.5803(13)	0.6714(13)	0.4471(4)	0.041(2)
O (5)	0.666667	0.333333	0.6904(10)	0.055(6)

Table S16. Selected bond distances (Å) and bond angles (deg) in compound **1**.

Bond distance (Å)			
La1-O6	2.486(2)	Fe1-O8	2.011(2)
La1-O7	2.471(2)	S1-O1	1.494(2)
La1-O9	2.468(2)	S1-O4	1.498(2)
La1-O10	2.471(2)	S1-O10	1.450(2)
La1-O11	2.491(2)	S1-O12	1.450(2)
La1-O12	2.460(2)	S2-O2	1.488(2)
La1-O13	2.495(3)	S2-O5	1.485(2)
La1-O14	2.529(3)	S2-O6	1.460(2)
Fe1-O1	2.020(2)	S2-O7	1.451(2)
Fe1-O2	2.019(2)	S3-O3	1.490(2)
Fe1-O3	2.007(2)	S3-O8	1.491(2)
Fe1-O4	1.995(2)	S3-O9	1.458(2)
Fe1-O5	2.043(2)	S3-O11	1.459(2)
Bond angle (deg)			
O6-La1-O11	121.22(8)	O4-Fe1-O5	96.31(10)
O6-La1-O13	79.71(9)	O4-Fe1-O8	175.33(10)
O6-La1-O14	139.84(9)	O8-Fe1-O1	89.98(10)
O7-La1-O6	70.53(8)	O8-Fe1-O2	88.72(9)
O7-La1-O10	116.21(8)	O8-Fe1-O5	79.62(9)
O7-La1-O11	78.36(8)	O1-S1-O4	104.59(13)
O7-La1-O13	141.97(8)	O10-S1-O1	110.88(14)
O7-La1-O14	145.80(8)	O10-S1-O4	110.00(15)
O9-La1-O6	135.81(8)	O12-S1-O1	110.15(14)
O9-La1-O7	81.42(8)	O12-S1-O4	110.31(15)
O9-La1-O10	153.44(8)	O12-S1-O10	110.74(15)
O9-La1-O11	83.73(8)	O5-S2-O2	106.12(13)
O9-La1-O13	105.83(9)	O6-S2-O2	108.56(14)
O9-La1-O14	79.89(9)	O6-S2-O5	109.30(14)
O10-La1-O6	70.70(8)	O7-S2-O2	110.92(14)
O10-La1-O11	80.97(8)	O7-S2-O5	109.64(14)
O10-La1-O13	73.25(8)	O7-S2-O6	112.12(15)
O10-La1-O14	74.70(9)	O3-S3-O8	104.96(13)
O11-La1-O13	138.85(9)	O9-S3-O3	111.53(14)
O11-La1-O14	71.34(8)	O9-S3-O8	109.90(14)
O12-La1-O6	70.66(8)	O9-S3-O11	110.94(14)
O12-La1-O7	78.55(8)	O11-S3-O3	109.87(14)

O12-La1-O9	70.78(8)	O11-S3-O8	109.47(15)
O12-La1-O10	129.82(8)	S1-O1-Fe1	145.94(15)
O12-La1-O11	147.74(8)	S2-O2-Fe1	134.60(14)
O12-La1-O13	69.42(9)	S3-O3-Fe1	140.68(14)
O12-La1-O14	120.95(9)	S1-O4-Fe1	135.90(15)
O13-La1-O14	71.23(9)	S2-O5-Fe1	140.91(14)
O1-Fe1-O5	92.31(10)	S2-O6-La1	144.39(15)
O2-Fe1-O1	91.72(10)	S2-O7-La1	145.98(15)
O2-Fe1-O5	167.66(9)	S3-O8-Fe1	139.06(14)
O3-Fe1-O1	178.94(10)	S3-O9-La1	157.39(15)
O3-Fe1-O2	87.70(9)	S1-O10-La1	158.81(16)
O3-Fe1-O5	88.44(9)	S3-O11-La1	143.81(15)
O3-Fe1-O8	90.89(10)	S1-O12-La1	158.19(16)
O4-Fe1-O1	87.87(10)		
O4-Fe1-O2	95.49(10)		

Table S17. Selected bond distances (Å) and bond angles (deg) in compound **2**.

Bond distance (Å)			
Ce1-O7	2.446(3)	Fe1-O6	2.005(3)
Ce1-O8	2.469(3)	S1-O2	1.491(3)
Ce1-O9	2.465(3)	S1-O5	1.492(3)
Ce1-O10	2.447(3)	S1-O11	1.451(3)
Ce1-O11	2.445(3)	S1-O12	1.447(3)
Ce1-O12	2.435(3)	S2-O4	1.489(3)
Ce1-O13	2.505(4)	S2-O6	1.488(3)
Ce1-O14	2.467(3)	S2-O9	1.459(3)
Fe1-O1	2.028(3)	S2-O10	1.452(3)
Fe1-O2	2.016(3)	S3-O1	1.483(3)
Fe1-O3	2.014(3)	S3-O3	1.485(3)
Fe1-O4	2.001(3)	S3-O7	1.455(3)
Fe1-O5	1.990(3)	S3-O8	1.460(3)
Bond angle (deg)			
O7-Ce1-O8	70.46(11)	O5-Fe1-O4	91.46(13)
O7-Ce1-O9	78.11(11)	O5-Fe1-O6	175.60(13)
O7-Ce1-O10	81.70(11)	O6-Fe1-O1	80.06(13)
O7-Ce1-O13	145.87(11)	O6-Fe1-O2	90.31(13)
O7-Ce1-O14	141.93(11)	O6-Fe1-O3	88.58(13)
O8-Ce1-O13	139.95(11)	O2-S1-O5	104.42(18)
O9-Ce1-O8	121.44(11)	O11-S1-O2	111.1(2)
O9-Ce1-O13	71.60(11)	O11-S1-O5	110.0(2)
O9-Ce1-O14	139.09(11)	O12-S1-O2	110.3(2)
O10-Ce1-O8	136.01(11)	O12-S1-O5	110.3(2)
O10-Ce1-O9	83.22(11)	O12-S1-O11	110.6(2)

O10-Ce1-O13	79.49(12)	O6-S2-O4	104.51(18)
O10-Ce1-O14	106.11(12)	O9-S2-O4	110.17(19)
O11-Ce1-O7	115.77(11)	O9-S2-O6	109.2(2)
O11-Ce1-O8	70.81(11)	O10-S2-O4	111.5(2)
O11-Ce1-O9	81.00(11)	O10-S2-O6	110.3(2)
O11-Ce1-O10	153.14(11)	O10-S2-O9	111.0(2)
O11-Ce1-O13	74.97(12)	O1-S3-O3	105.89(18)
O11-Ce1-O14	73.41(12)	O7-S3-O1	109.41(19)
O12-Ce1-O7	78.81(11)	O7-S3-O3	111.31(19)
O12-Ce1-O8	70.89(11)	O7-S3-O8	112.1(2)
O12-Ce1-O9	147.30(11)	O8-S3-O1	109.28(19)
O12-Ce1-O10	70.77(11)	O8-S3-O3	108.60(19)
O12-Ce1-O11	130.29(11)	S3-O1-Fe1	141.2(2)
O12-Ce1-O13	120.46(12)	S1-O2-Fe1	146.3(2)
O12-Ce1-O14	69.47(12)	S3-O3-Fe1	134.5(2)
O14-Ce1-O8	79.47(12)	S2-O4-Fe1	140.56(19)
O14-Ce1-O13	71.25(12)	S1-O5-Fe1	136.0(2)
O2-Fe1-O1	92.25(13)	S2-O6-Fe1	139.07(19)
O3-Fe1-O1	168.00(13)	S3-O7-Ce1	146.0(2)
O3-Fe1-O2	91.65(13)	S3-O8-Ce1	144.7(2)
O4-Fe1-O1	88.44(13)	S2-O9-Ce1	144.9(2)
O4-Fe1-O2	178.88(13)	S2-O10-Ce1	157.9(2)
O4-Fe1-O3	87.84(13)	S1-O11-Ce1	159.3(2)
O4-Fe1-O6	90.67(13)	S1-O12-Ce1	158.1(2)
O5-Fe1-O1	96.15(13)		
O5-Fe1-O2	87.59(13)		

Table S18. Selected bond distances (Å) and bond angles (deg) in compound **3**.

Bond distance (Å)			
Pr1-O2	2.451(2)	Fe1-O14	2.010(2)
Pr1-O4	2.433(2)	S1-O3	1.495(2)
Pr1-O5	2.447(2)	S1-O7	1.449(2)
Pr1-O6	2.430(2)	S1-O8	1.446(2)
Pr1-O7	2.429(2)	S1-O11	1.487(2)
Pr1-O8	2.417(2)	S2-O1	1.487(2)
Pr1-O9	2.453(3)	S2-O5	1.459(2)
Pr1-O10	2.492(2)	S2-O6	1.456(2)
Fe1-O1	1.999(2)	S2-O13	1.485(2)
Fe1-O3	1.984(2)	S3-O2	1.458(2)
Fe1-O11	2.014(2)	S3-O4	1.452(2)
Fe1-O12	2.024(2)	S3-O12	1.480(2)
Fe1-O13	2.005(2)	S3-O14	1.482(2)
Bond angle (deg)			

O2-Pr1-O9	79.03(8)	O11-Fe1-O12	91.92(10)
O2-Pr1-O10	139.91(8)	O13-Fe1-O11	90.21(9)
O4-Pr1-O2	70.90(8)	O13-Fe1-O12	80.24(9)
O4-Pr1-O5	77.93(8)	O13-Fe1-O14	88.48(9)
O4-Pr1-O9	142.34(8)	O14-Fe1-O11	91.45(9)
O4-Pr1-O10	145.47(8)	O14-Fe1-O12	168.23(9)
O5-Pr1-O2	121.99(8)	O7-S1-O3	110.06(15)
O5-Pr1-O9	138.81(9)	O7-S1-O11	111.34(14)
O5-Pr1-O10	71.33(8)	O8-S1-O3	110.36(15)
O6-Pr1-O2	136.57(7)	O8-S1-O7	110.39(14)
O6-Pr1-O4	82.20(8)	O8-S1-O11	110.34(14)
O6-Pr1-O5	82.80(8)	O11-S1-O3	104.21(12)
O6-Pr1-O9	106.23(9)	O5-S2-O1	109.88(14)
O6-Pr1-O10	78.78(9)	O5-S2-O13	109.47(14)
O7-Pr1-O2	70.94(8)	O6-S2-O1	111.61(13)
O7-Pr1-O4	115.39(8)	O6-S2-O5	111.14(14)
O7-Pr1-O5	80.73(8)	O6-S2-O13	110.16(14)
O7-Pr1-O6	152.46(8)	O13-S2-O1	104.34(13)
O7-Pr1-O9	73.48(9)	O2-S3-O12	108.89(13)
O7-Pr1-O10	75.11(9)	O2-S3-O14	108.66(13)
O8-Pr1-O2	71.02(8)	O4-S3-O2	112.15(14)
O8-Pr1-O4	79.51(8)	O4-S3-O12	109.85(14)
O8-Pr1-O5	147.38(8)	O4-S3-O14	111.25(14)
O8-Pr1-O6	71.01(8)	O12-S3-O14	105.81(13)
O8-Pr1-O7	130.67(8)	S2-O1-Fe1	140.86(14)
O8-Pr1-O9	69.44(9)	S3-O2-Pr1	145.40(14)
O8-Pr1-O10	120.02(9)	S1-O3-Fe1	136.32(14)
O9-Pr1-O10	71.33(9)	S3-O4-Pr1	146.22(14)
O1-Fe1-O11	178.98(9)	S2-O5-Pr1	145.49(15)
O1-Fe1-O12	88.56(9)	S2-O6-Pr1	158.01(14)
O1-Fe1-O13	90.75(9)	S1-O7-Pr1	159.92(16)
O1-Fe1-O14	88.25(9)	S1-O8-Pr1	157.76(16)
O3-Fe1-O1	91.37(9)	S1-O11-Fe1	146.36(14)
O3-Fe1-O11	87.69(9)	S3-O12-Fe1	141.30(14)
O3-Fe1-O12	96.21(10)	S2-O13-Fe1	139.20(14)
O3-Fe1-O13	175.82(10)	S3-O14-Fe1	134.72(13)
O3-Fe1-O14	95.19(10)	œ	

Table S19. Selected bond distances (Å) and bond angles (deg) in compound **4**.

Bond distance (Å)			
Nd1-O7	2.434(3)	Fe1-O6	1.996(3)
Nd1-O8	2.408(3)	S1-O1	1.478(3)
Nd1-O9	2.420(3)	S1-O5	1.482(3)

Nd1-O10	2.404(3)	S1-O11	1.442(3)
Nd1-O11	2.401(3)	S1-O14	1.436(3)
Nd1-O12	2.434(3)	S2-O2	1.475(3)
Nd1-O13	2.458(3)	S2-O6	1.478(3)
Nd1-O14	2.390(3)	S2-O7	1.448(3)
Fe1-O1	2.004(3)	S2-O8	1.441(3)
Fe1-O2	2.010(3)	S3-O3	1.478(3)
Fe1-O3	1.998(3)	S3-O4	1.477(3)
Fe1-O4	1.988(3)	S3-O9	1.452(3)
Fe1-O5	1.971(3)	S3-O10	1.449(3)
Bond angle (deg)			
O7-Nd1-O13	139.74(11)	O5-Fe1-O4	91.58(12)
O8-Nd1-O7	71.12(10)	O5-Fe1-O6	95.08(13)
O8-Nd1-O9	77.60(10)	O6-Fe1-O1	91.37(12)
O8-Nd1-O12	142.45(11)	O6-Fe1-O2	167.99(11)
O8-Nd1-O13	145.47(10)	O6-Fe1-O3	88.09(12)
O9-Nd1-O7	122.33(10)	O1-S1-O5	104.31(17)
O9-Nd1-O12	138.99(11)	O11-S1-O1	111.30(18)
O9-Nd1-O13	71.54(11)	O11-S1-O5	109.99(19)
O10-Nd1-O7	137.07(10)	O14-S1-O1	110.40(18)
O10-Nd1-O8	82.61(10)	O14-S1-O5	110.35(19)
O10-Nd1-O9	82.22(10)	O14-S1-O11	110.34(19)
O10-Nd1-O12	106.37(11)	O2-S2-O6	105.38(17)
O10-Nd1-O13	78.28(11)	O7-S2-O2	108.93(17)
O11-Nd1-O7	70.75(10)	O7-S2-O6	109.01(17)
O11-Nd1-O8	114.80(10)	O8-S2-O2	110.00(18)
O11-Nd1-O9	80.82(10)	O8-S2-O6	111.19(18)
O11-Nd1-O10	152.15(10)	O8-S2-O7	112.09(18)
O11-Nd1-O12	73.72(11)	O4-S3-O3	104.28(16)
O11-Nd1-O13	75.47(11)	O9-S3-O3	109.30(18)
O12-Nd1-O7	78.67(11)	O9-S3-O4	110.43(18)
O12-Nd1-O13	71.27(11)	O10-S3-O3	110.35(18)
O14-Nd1-O7	71.17(10)	O10-S3-O4	111.43(18)
O14-Nd1-O8	79.83(11)	O10-S3-O9	110.86(17)
O14-Nd1-O9	146.98(11)	S1-O1-Fe1	146.88(18)
O14-Nd1-O10	71.19(10)	S2-O2-Fe1	141.08(19)
O14-Nd1-O11	130.98(11)	S3-O3-Fe1	139.28(18)
O14-Nd1-O12	69.51(11)	S3-O4-Fe1	141.16(18)
O14-Nd1-O13	119.67(11)	S1-O5-Fe1	136.44(18)
O1-Fe1-O2	91.76(12)	S2-O6-Fe1	134.59(18)
O3-Fe1-O1	90.57(12)	S2-O7-Nd1	145.96(18)
O3-Fe1-O2	80.29(12)	S2-O8-Nd1	146.15(19)
O4-Fe1-O1	179.02(12)	S3-O9-Nd1	146.49(18)
O4-Fe1-O2	88.40(12)	S3-O10-Nd1	158.02(19)
O4-Fe1-O3	90.41(13)	S1-O11-Nd1	160.7(2)

O4-Fe1-O6	88.66(12)	S1-O14-Nd1	157.9(2)
O5-Fe1-O1	87.44(12)		
O5-Fe1-O2	96.64(13)		

Table S20. Selected bond distances (Å) and bond angles (deg) in compound **5**.

Bond distance (Å)			
Sm1-O6	2.420(3)	Fe1-O8	2.004(3)
Sm1-O7	2.398(3)	S1-O2	1.483(3)
Sm1-O9	2.394(3)	S1-O4	1.488(3)
Sm1-O10	2.412(3)	S1-O12	1.445(3)
Sm1-O11	2.421(3)	S1-O14	1.450(3)
Sm1-O12	2.371(3)	S2-O1	1.483(3)
Sm1-O13	2.441(3)	S2-O6	1.452(3)
Sm1-O14	2.387(3)	S2-O7	1.455(3)
Fe1-O1	2.013(3)	S2-O8	1.484(3)
Fe1-O2	2.005(3)	S3-O3	1.488(3)
Fe1-O3	1.989(3)	S3-O5	1.485(3)
Fe1-O4	1.986(3)	S3-O9	1.455(3)
Fe1-O5	1.990(3)	S3-O10	1.457(3)
Bond angle (deg)			
O6-Sm1-O11	78.52(10)	O5-Fe1-O1	87.91(12)
O6-Sm1-O13	139.66(10)	O5-Fe1-O2	178.42(12)
O7-Sm1-O6	71.24(10)	O5-Fe1-O8	89.29(11)
O7-Sm1-O10	77.40(10)	O8-Fe1-O1	168.37(11)
O7-Sm1-O11	142.62(10)	O8-Fe1-O2	91.17(12)
O7-Sm1-O13	145.46(10)	O2-S1-O4	103.90(16)
O9-Sm1-O6	137.55(9)	O12-S1-O2	110.64(18)
O9-Sm1-O7	82.74(10)	O12-S1-O4	110.42(18)
O9-Sm1-O10	81.85(9)	O12-S1-O14	110.40(18)
O9-Sm1-O11	106.81(11)	O14-S1-O2	111.05(17)
O9-Sm1-O13	77.93(11)	O14-S1-O4	110.27(18)
O10-Sm1-O6	122.24(10)	O1-S2-O8	105.09(16)
O10-Sm1-O11	138.93(10)	O6-S2-O1	109.42(16)
O10-Sm1-O13	71.75(10)	O6-S2-O7	112.29(17)
O11-Sm1-O13	71.16(11)	O6-S2-O8	109.14(16)
O12-Sm1-O6	71.68(10)	O7-S2-O1	109.54(17)
O12-Sm1-O7	79.90(10)	O7-S2-O8	111.10(17)
O12-Sm1-O9	71.15(10)	O5-S3-O3	104.08(16)
O12-Sm1-O10	146.56(10)	O9-S3-O3	110.55(17)
O12-Sm1-O11	69.95(11)	O9-S3-O5	111.48(16)
O12-Sm1-O13	119.33(11)	O9-S3-O10	111.03(17)
O12-Sm1-O14	132.03(10)	O10-S3-O3	109.30(17)
O14-Sm1-O6	70.94(10)	O10-S3-O5	110.19(17)

O14-Sm1-O7	114.39(10)	S2-O1-Fe1	141.44(18)
O14-Sm1-O9	151.50(10)	S1-O2-Fe1	147.44(18)
O14-Sm1-O10	80.20(10)	S3-O3-Fe1	139.16(17)
O14-Sm1-O11	74.05(10)	S1-O4-Fe1	136.38(17)
O14-Sm1-O13	75.52(10)	S3-O5-Fe1	141.61(18)
O2-Fe1-O1	91.93(12)	S2-O6-Sm1	146.45(17)
O3-Fe1-O1	80.80(12)	S2-O7-Sm1	146.45(18)
O3-Fe1-O2	90.97(12)	S2-O8-Fe1	134.43(17)
O3-Fe1-O5	90.56(12)	S3-O9-Sm1	158.18(18)
O3-Fe1-O8	87.95(11)	S3-O10-Sm1	146.92(18)
O4-Fe1-O1	96.39(12)	S1-O12-Sm1	158.4(2)
O4-Fe1-O2	87.24(11)	S1-O14-Sm1	162.0(2)
O4-Fe1-O3	176.62(12)		
O4-Fe1-O5	91.21(11)		

Table S21. Selected bond distances (Å) and bond angles (deg) in compound **6.**

Bond distance (Å)			
Eu1-O7	2.386(2)	Fe1-O6	2.007(2)
Eu1-O8	2.405(2)	S1-O2	1.492(2)
Eu1-O9	2.383(2)	S1-O3	1.485(2)
Eu1-O10	2.399(2)	S1-O13	1.448(3)
Eu1-O11	2.411(3)	S1-O14	1.449(3)
Eu1-O12	2.433(3)	S2-O4	1.480(2)
Eu1-O13	2.363(2)	S2-O6	1.486(2)
Eu1-O14	2.380(2)	S2-O7	1.456(2)
Fe1-O1	2.001(2)	S2-O8	1.456(2)
Fe1-O2	1.972(2)	S3-O1	1.488(2)
Fe1-O3	2.007(2)	S3-O5	1.486(2)
Fe1-O4	2.020(2)	S3-O9	1.459(2)
Fe1-O5	1.989(2)	S3-O10	1.454(2)
Bond angle (deg)			
O7-Eu1-O8	71.39(8)	O5-Fe1-O1	90.28(10)
O7-Eu1-O10	77.23(9)	O5-Fe1-O3	178.92(10)
O7-Eu1-O11	142.67(9)	O5-Fe1-O4	88.21(10)
O7-Eu1-O12	145.42(9)	O5-Fe1-O6	89.63(10)
O8-Eu1-O11	78.23(9)	O6-Fe1-O4	168.33(9)
O8-Eu1-O12	139.53(9)	O3-S1-O2	103.92(14)
O9-Eu1-O7	83.31(9)	O13-S1-O2	110.18(16)
O9-Eu1-O8	137.97(8)	O13-S1-O3	110.49(15)
O9-Eu1-O10	81.53(8)	O13-S1-O14	110.23(15)
O9-Eu1-O11	106.63(10)	O14-S1-O2	110.23(16)
O9-Eu1-O12	77.46(9)	O14-S1-O3	111.63(15)
O10-Eu1-O8	122.61(8)	O4-S2-O6	104.93(14)

O10-Eu1-O11	139.04(9)	O7-S2-O4	109.87(15)
O10-Eu1-O12	71.74(9)	O7-S2-O6	111.31(14)
O11-Eu1-O12	71.20(9)	O8-S2-O4	109.30(14)
O13-Eu1-O7	80.41(9)	O8-S2-O6	108.90(14)
O13-Eu1-O8	71.96(9)	O8-S2-O7	112.26(14)
O13-Eu1-O9	71.11(9)	O5-S3-O1	103.63(14)
O13-Eu1-O10	146.36(9)	O9-S3-O1	110.54(15)
O13-Eu1-O11	69.76(10)	O9-S3-O5	111.72(14)
O13-Eu1-O12	118.87(10)	O10-S3-O1	109.62(15)
O13-Eu1-O14	132.51(9)	O10-S3-O5	110.35(15)
O14-Eu1-O7	113.90(9)	O10-S3-O9	110.76(14)
O14-Eu1-O8	71.03(8)	S3-O1-Fe1	139.09(15)
O14-Eu1-O9	151.00(9)	S1-O2-Fe1	136.70(15)
O14-Eu1-O10	80.04(9)	S1-O3-Fe1	147.36(15)
O14-Eu1-O11	74.45(9)	S2-O4-Fe1	141.47(15)
O14-Eu1-O12	75.60(9)	S3-O5-Fe1	141.59(15)
O1-Fe1-O3	90.58(10)	S2-O6-Fe1	134.65(15)
O1-Fe1-O4	80.72(10)	S2-O7-Eu1	146.39(15)
O1-Fe1-O6	87.83(10)	S2-O8-Eu1	147.02(15)
O2-Fe1-O1	176.61(10)	S3-O9-Eu1	158.56(16)
O2-Fe1-O3	87.40(10)	S3-O10-Eu1	148.15(15)
O2-Fe1-O4	96.60(11)	S1-O13-Eu1	158.27(17)
O2-Fe1-O5	91.71(10)	S1-O14-Eu1	162.51(18)
O2-Fe1-O6	94.92(11)		
O3-Fe1-O4	91.30(10)		

Table S22. Selected bond distances (Å) and bond angles (deg) in compound **7**.

Bond distance (Å)			
Gd1-O4	2.375(3)	Fe1-O6	2.012(3)
Gd1-O8	2.375(3)	Fe1-O7	2.004(3)
Gd1-O9	2.394(3)	S1-O2	1.489(3)
Gd1-O10	2.346(3)	S1-O3	1.481(3)
Gd1-O11	2.386(3)	S1-O10	1.450(3)
Gd1-O12	2.397(3)	S1-O13	1.451(3)
Gd1-O13	2.363(3)	S2-O4	1.457(3)
Gd1-O14	2.421(3)	S2-O6	1.481(3)
Fe1-O1	1.996(3)	S2-O7	1.484(3)
Fe1-O2	1.969(3)	S2-O9	1.457(3)
Fe1-O3	2.006(3)	S3-O1	1.486(3)
Fe1-O5	1.989(3)	S3-O5	1.482(3)
Gd1-O4	2.375(3)	S3-O8	1.452(3)
Bond angle (deg)			
O4-Gd1-O8	83.66(10)	O5-Fe1-O3	178.70(11)

O4-Gd1-O9	71.31(9)	O5-Fe1-O6	87.98(11)
O4-Gd1-O11	77.27(10)	O5-Fe1-O7	89.91(11)
O4-Gd1-O12	142.65(10)	O7-Fe1-O3	90.87(11)
O4-Gd1-O14	145.60(10)	O7-Fe1-O6	168.30(11)
O8-Gd1-O9	138.25(10)	O3-S1-O2	103.82(16)
O8-Gd1-O11	81.48(10)	O10-S1-O2	110.27(17)
O8-Gd1-O12	106.67(10)	O10-S1-O3	110.77(17)
O8-Gd1-O14	77.11(10)	O10-S1-O13	110.25(17)
O9-Gd1-O12	78.15(10)	O13-S1-O2	110.11(17)
O9-Gd1-O14	139.49(10)	O13-S1-O3	111.46(17)
O10-Gd1-O4	80.62(10)	O4-S2-O6	109.87(16)
O10-Gd1-O8	71.12(10)	O4-S2-O7	111.37(16)
O10-Gd1-O9	72.19(10)	O6-S2-O7	104.80(16)
O10-Gd1-O11	146.38(10)	O9-S2-O4	112.07(16)
O10-Gd1-O12	69.78(11)	O9-S2-O6	109.53(16)
O10-Gd1-O13	132.78(10)	O9-S2-O7	108.93(16)
O10-Gd1-O14	118.45(11)	O5-S3-O1	103.54(16)
O11-Gd1-O9	122.58(10)	O8-S3-O1	110.44(17)
O11-Gd1-O12	138.95(11)	O8-S3-O5	111.82(16)
O11-Gd1-O14	71.90(10)	O8-S3-O11	110.90(17)
O12-Gd1-O14	71.07(11)	O11-S3-O1	109.53(16)
O13-Gd1-O4	113.58(10)	O11-S3-O5	110.37(16)
O13-Gd1-O8	150.73(10)	S3-O1-Fe1	139.17(18)
O13-Gd1-O9	71.02(10)	S1-O2-Fe1	136.84(18)
O13-Gd1-O11	79.79(10)	S1-O3-Fe1	147.59(17)
O13-Gd1-O12	74.55(11)	S2-O4-Gd1	146.13(17)
O13-Gd1-O14	75.80(10)	S3-O5-Fe1	141.68(18)
O1-Fe1-O3	90.86(11)	S2-O6-Fe1	141.43(18)
O1-Fe1-O6	80.83(11)	S2-O7-Fe1	134.63(17)
O1-Fe1-O7	87.67(11)	S3-O8-Gd1	158.50(18)
O2-Fe1-O1	176.97(12)	S2-O9-Gd1	147.30(17)
O2-Fe1-O3	87.33(11)	S1-O10-Gd1	158.23(19)
O2-Fe1-O5	91.56(11)	S3-O11-Gd1	148.16(18)
O2-Fe1-O6	96.77(12)	S1-O13-Gd1	162.87(19)
O2-Fe1-O7	94.78(12)		
O3-Fe1-O6	91.46(11)		

Table S23. Selected bond distances (Å) and bond angles (deg) in compound **8**.

Bond distance (Å)			
Dy1-O7	2.360(3)	Fe1-O6	1.964(3)
Dy1-O8	2.356(3)	S1-O1	1.479(3)
Dy1-O9	2.396(3)	S1-O6	1.488(3)
Dy1-O10	2.371(3)	S1-O13	1.445(3)

Dy1-O11	2.350(3)	S1-O14	1.447(3)
Dy1-O12	2.385(3)	S2-O2	1.477(3)
Dy1-O13	2.324(3)	S2-O4	1.484(3)
Dy1-O14	2.343(3)	S2-O8	1.455(3)
Fe1-O1	2.001(3)	S2-O10	1.454(3)
Fe1-O2	2.012(3)	S3-O3	1.484(3)
Fe1-O3	1.996(3)	S3-O5	1.485(3)
Fe1-O4	1.998(3)	S3-O7	1.456(3)
Fe1-O5	1.981(3)	S3-O11	1.457(3)
Bond angle (deg)			
O7-Dy1-O9	72.23(10)	O6-Fe1-O1	87.23(11)
O7-Dy1-O10	122.87(10)	O6-Fe1-O2	96.71(12)
O7-Dy1-O12	138.90(11)	O6-Fe1-O3	177.12(12)
O8-Dy1-O7	76.91(10)	O6-Fe1-O4	94.63(12)
O8-Dy1-O9	145.61(10)	O6-Fe1-O5	91.65(12)
O8-Dy1-O10	71.70(9)	O1-S1-O6	103.69(16)
O8-Dy1-O12	143.00(10)	O13-S1-O1	110.61(17)
O10-Dy1-O9	139.18(10)	O13-S1-O6	110.27(18)
O10-Dy1-O12	77.82(10)	O13-S1-O14	110.23(17)
O11-Dy1-O7	81.06(10)	O14-S1-O1	111.69(18)
O11-Dy1-O8	83.89(10)	O14-S1-O6	110.20(19)
O11-Dy1-O9	76.76(11)	O2-S2-O4	104.57(16)
O11-Dy1-O10	138.69(10)	O8-S2-O2	109.76(17)
O11-Dy1-O12	106.91(11)	O8-S2-O4	111.48(17)
O12-Dy1-O9	70.76(11)	O10-S2-O2	109.59(16)
O13-Dy1-O7	146.04(10)	O10-S2-O4	109.01(16)
O13-Dy1-O8	81.04(10)	O10-S2-O8	112.15(16)
O13-Dy1-O9	117.88(11)	O3-S3-O5	103.19(16)
O13-Dy1-O10	72.49(10)	O7-S3-O3	109.43(17)
O13-Dy1-O11	71.13(10)	O7-S3-O5	110.64(17)
O13-Dy1-O12	70.00(11)	O7-S3-O11	110.89(17)
O13-Dy1-O14	133.63(11)	O11-S3-O3	110.75(17)
O14-Dy1-O7	79.33(10)	O11-S3-O5	111.68(16)
O14-Dy1-O8	113.05(10)	S1-O1-Fe1	148.27(18)
O14-Dy1-O9	75.89(11)	S2-O2-Fe1	141.61(18)
O14-Dy1-O10	71.21(10)	S3-O3-Fe1	139.12(17)
O14-Dy1-O11	150.10(10)	S2-O4-Fe1	134.83(17)
O14-Dy1-O12	74.93(11)	S3-O5-Fe1	141.94(18)
O1-Fe1-O2	91.10(12)	S1-O6-Fe1	137.06(18)
O3-Fe1-O1	90.92(11)	S3-O7-Dy1	149.23(18)
O3-Fe1-O2	81.12(11)	S2-O8-Dy1	146.38(17)
O3-Fe1-O4	87.59(11)	S2-O10-Dy1	148.18(17)
O4-Fe1-O1	90.78(12)	S3-O11-Dy1	159.03(18)
O4-Fe1-O2	168.58(10)	S1-O13-Dy1	158.4(2)
O5-Fe1-O1	178.36(12)	S1-O14-Dy1	163.8(2)

O5-Fe1-O2	87.84(12)		
O5-Fe1-O3	90.16(12)		

Table S24. Selected bond distances (Å) and bond angles (deg) in compound **9**.

Bond distance (Å)			
Ho1-O5	2.342(4)	Fe1-O7	2.010(4)
Ho1-O8	2.367(4)	S1-O1	1.484(4)
Ho1-O9	2.345(4)	S1-O3	1.484(4)
Ho1-O10	2.352(4)	S1-O12	1.447(4)
Ho1-O11	2.383(4)	S1-O13	1.445(4)
Ho1-O12	2.313(4)	S2-O5	1.461(4)
Ho1-O13	2.334(4)	S2-O6	1.486(4)
Ho1-O14	2.369(4)	S2-O7	1.478(4)
Fe1-O1	1.965(4)	S2-O8	1.454(4)
Fe1-O2	1.993(4)	S3-O2	1.484(4)
Fe1-O3	1.999(4)	S3-O4	1.484(4)
Fe1-O4	1.980(4)	S3-O9	1.457(4)
Fe1-O6	1.996(4)	S3-O10	1.458(4)
Bond angle (deg)			
O5-Ho1-O8	71.88(12)	O4-Fe1-O3	178.22(15)
O5-Ho1-O9	84.05(13)	O4-Fe1-O6	90.82(15)
O5-Ho1-O10	76.81(13)	O4-Fe1-O7	87.74(15)
O5-Ho1-O11	145.59(13)	O6-Fe1-O3	90.68(15)
O5-Ho1-O14	143.03(13)	O6-Fe1-O7	168.45(14)
O8-Ho1-O11	139.02(13)	O3-S1-O1	103.5(2)
O8-Ho1-O14	77.46(13)	O12-S1-O1	110.0(2)
O9-Ho1-O8	139.13(13)	O12-S1-O3	110.6(2)
O9-Ho1-O10	80.61(13)	O13-S1-O1	110.6(2)
O9-Ho1-O11	76.49(14)	O13-S1-O3	111.9(2)
O9-Ho1-O14	107.35(14)	O13-S1-O12	110.1(2)
O10-Ho1-O8	123.04(13)	O5-S2-O6	111.3(2)
O10-Ho1-O11	72.24(13)	O5-S2-O7	110.0(2)
O10-Ho1-O14	138.86(14)	O7-S2-O6	104.3(2)
O12-Ho1-O5	81.31(13)	O8-S2-O5	112.3(2)
O12-Ho1-O8	72.72(13)	O8-S2-O6	108.9(2)
O12-Ho1-O9	71.34(13)	O8-S2-O7	109.8(2)
O12-Ho1-O10	145.92(14)	O2-S3-O4	103.5(2)
O12-Ho1-O11	117.62(14)	O9-S3-O2	110.6(2)
O12-Ho1-O13	134.04(14)	O9-S3-O4	111.8(2)
O12-Ho1-O14	70.10(14)	O9-S3-O10	110.5(2)
O13-Ho1-O5	112.70(13)	O10-S3-O2	109.5(2)
O13-Ho1-O8	71.23(13)	O10-S3-O4	110.7(2)
O13-Ho1-O9	149.63(13)	S1-O1-Fe1	137.2(2)

O13-Ho1-O10	79.08(14)	S3-O2-Fe1	139.6(2)
O13-Ho1-O11	75.95(14)	S1-O3-Fe1	148.4(2)
O13-Ho1-O14	75.02(14)	S3-O4-Fe1	142.2(2)
O14-Ho1-O11	70.81(14)	S2-O5-Ho1	146.5(2)
O1-Fe1-O2	177.31(16)	S2-O6-Fe1	134.9(2)
O1-Fe1-O3	87.30(15)	S2-O7-Fe1	141.4(2)
O1-Fe1-O4	91.62(15)	S2-O8-Ho1	148.7(2)
O1-Fe1-O6	94.80(15)	S3-O9-Ho1	158.7(2)
O1-Fe1-O7	96.70(15)	S3-O10-Ho1	149.7(2)
O2-Fe1-O3	91.05(15)	S1-O12-Ho1	158.5(3)
O2-Fe1-O6	87.34(15)	S1-O13-Ho1	164.3(3)
O2-Fe1-O7	81.21(15)		
O3-Fe1-O7	90.99(15)		

Table S25. Selected bond distances (Å) and bond angles (deg) in compound **10.**

Bond distance (Å)			
Er1-O7	2.344(3)	Fe1-O6	1.977(3)
Er1-O8	2.335(3)	S1-O1	1.489(3)
Er1-O9	2.341(3)	S1-O2	1.476(3)
Er1-O10	2.363(3)	S1-O13	1.452(3)
Er1-O11	2.359(3)	S1-O14	1.443(3)
Er1-O12	2.377(3)	S2-O3	1.479(3)
Er1-O13	2.324(3)	S2-O4	1.487(3)
Er1-O14	2.309(3)	S2-O9	1.458(3)
Fe1-O1	1.972(3)	S2-O11	1.452(3)
Fe1-O2	1.994(3)	S3-O5	1.489(3)
Fe1-O3	2.001(3)	S3-O6	1.487(3)
Fe1-O4	2.000(3)	S3-O7	1.457(3)
Fe1-O5	1.979(3)	S3-O8	1.460(3)
Bond angle (deg)			
O7-Er1-O10	138.88(11)	O5-Fe1-O4	87.67(12)
O7-Er1-O11	122.86(11)	O6-Fe1-O2	177.54(12)
O7-Er1-O12	72.42(11)	O6-Fe1-O3	87.68(12)
O8-Er1-O7	80.70(10)	O6-Fe1-O4	90.73(12)
O8-Er1-O9	83.98(11)	O6-Fe1-O5	90.25(13)
O8-Er1-O10	107.36(12)	O2-S1-O1	103.69(17)
O8-Er1-O11	139.00(10)	O13-S1-O1	110.2(2)
O8-Er1-O12	76.37(12)	O13-S1-O2	111.51(19)
O9-Er1-O7	76.76(11)	O14-S1-O1	110.51(19)
O9-Er1-O10	143.06(11)	O14-S1-O2	110.40(18)
O9-Er1-O11	71.73(10)	O14-S1-O13	110.39(19)
O9-Er1-O12	145.59(11)	O3-S2-O4	104.50(17)
O10-Er1-O12	70.75(12)	O9-S2-O3	109.84(18)

O11-Er1-O10	77.64(11)	O9-S2-O4	111.36(18)
O11-Er1-O12	139.27(11)	O11-S2-O3	109.43(18)
O13-Er1-O7	78.82(11)	O11-S2-O4	108.99(17)
O13-Er1-O8	149.66(11)	O11-S2-O9	112.42(18)
O13-Er1-O9	112.49(11)	O6-S3-O5	103.00(17)
O13-Er1-O10	75.30(12)	O7-S3-O5	109.65(19)
O13-Er1-O11	71.33(11)	O7-S3-O6	110.75(18)
O13-Er1-O12	76.24(12)	O7-S3-O8	110.55(18)
O14-Er1-O7	145.89(11)	O8-S3-O5	110.77(18)
O14-Er1-O8	71.30(11)	O8-S3-O6	111.89(18)
O14-Er1-O9	81.21(11)	S1-O1-Fe1	137.4(2)
O14-Er1-O10	70.19(12)	S1-O2-Fe1	149.13(19)
O14-Er1-O11	72.68(11)	S2-O3-Fe1	142.3(2)
O14-Er1-O12	117.52(12)	S2-O4-Fe1	134.69(18)
O14-Er1-O13	134.27(11)	S3-O5-Fe1	139.00(18)
O1-Fe1-O2	86.97(13)	S3-O6-Fe1	141.96(18)
O1-Fe1-O3	96.53(13)	S3-O7-Er1	149.76(19)
O1-Fe1-O4	94.12(13)	S3-O8-Er1	158.9(2)
O1-Fe1-O5	177.81(13)	S2-O9-Er1	146.32(19)
O1-Fe1-O6	90.99(13)	S2-O11-Er1	148.33(19)
O2-Fe1-O3	91.18(13)	S1-O13-Er1	164.7(2)
O2-Fe1-O4	90.79(12)	S1-O14-Er1	158.8(2)
O4-Fe1-O3	169.26(12)		
O5-Fe1-O2	91.75(13)		

Table S26. Selected bond distances (Å) and bond angles (deg) in compound **11**.

Bond distance (Å)			
Tm1-O5	2.324(3)	Fe1-O8	1.978(3)
Tm1-O7	2.333(3)	S1-O1	1.481(3)
Tm1-O9	2.348(3)	S1-O6	1.482(3)
Tm1-O10	2.324(3)	S1-O12	1.446(3)
Tm1-O11	2.366(3)	S1-O14	1.448(3)
Tm1-O12	2.292(3)	S2-O2	1.479(3)
Tm1-O13	2.363(3)	S2-O4	1.480(3)
Tm1-O14	2.312(3)	S2-O9	1.449(3)
Fe1-O1	1.966(3)	S2-O10	1.463(3)
Fe1-O2	1.998(3)	S3-O3	1.487(3)
Fe1-O3	1.983(3)	S3-O5	1.460(3)
Fe1-O4	2.007(3)	S3-O7	1.457(3)
Fe1-O6	1.986(3)	S3-O8	1.478(3)
Bond angle (deg)			
O5-Tm1-O7	80.45(11)	O6-Fe1-O4	91.07(13)
O5-Tm1-O9	139.29(11)	O8-Fe1-O2	91.41(13)

O5-Tm1-O11	107.85(12)	O8-Fe1-O3	89.86(14)
O5-Tm1-O13	76.41(12)	O8-Fe1-O4	87.25(13)
O7-Tm1-O9	123.13(11)	O8-Fe1-O6	177.64(13)
O7-Tm1-O11	138.63(12)	O1-S1-O6	103.79(19)
O7-Tm1-O13	72.25(12)	O12-S1-O1	110.2(2)
O9-Tm1-O11	77.17(12)	O12-S1-O6	110.44(19)
O9-Tm1-O13	138.93(12)	O12-S1-O14	110.1(2)
O10-Tm1-O5	83.98(11)	O14-S1-O1	110.8(2)
O10-Tm1-O7	76.88(11)	O14-S1-O6	111.4(2)
O10-Tm1-O9	72.00(12)	O2-S2-O4	104.0(2)
O10-Tm1-O11	143.09(12)	O9-S2-O2	109.19(19)
O10-Tm1-O13	145.61(12)	O9-S2-O4	109.82(19)
O12-Tm1-O5	71.33(11)	O9-S2-O10	112.3(2)
O12-Tm1-O7	145.84(11)	O10-S2-O2	111.60(19)
O12-Tm1-O9	72.87(11)	O10-S2-O4	109.67(19)
O12-Tm1-O10	81.30(11)	O5-S3-O3	111.06(18)
O12-Tm1-O11	70.47(12)	O5-S3-O8	111.81(19)
O12-Tm1-O13	117.46(12)	O7-S3-O3	109.38(19)
O12-Tm1-O14	134.82(12)	O7-S3-O5	110.52(18)
O13-Tm1-O11	70.77(12)	O7-S3-O8	110.58(19)
O14-Tm1-O5	149.36(11)	O8-S3-O3	103.28(18)
O14-Tm1-O7	78.35(12)	S1-O1-Fe1	137.6(2)
O14-Tm1-O9	71.33(11)	S2-O2-Fe1	134.9(2)
O14-Tm1-O10	112.10(12)	S3-O3-Fe1	139.35(19)
O14-Tm1-O11	75.45(12)	S2-O4-Fe1	141.4(2)
O14-Tm1-O13	76.22(12)	S3-O5-Tm1	159.1(2)
O1-Fe1-O2	94.28(13)	S1-O6-Fe1	149.5(2)
O1-Fe1-O3	177.72(14)	S3-O7-Tm1	150.10(19)
O1-Fe1-O4	96.89(13)	S3-O8-Fe1	142.6(2)
O1-Fe1-O6	87.08(13)	S2-O9-Tm1	149.2(2)
O1-Fe1-O8	91.48(14)	S2-O10-Tm1	146.7(2)
O2-Fe1-O4	168.78(13)	S1-O12-Tm1	158.9(2)
O3-Fe1-O2	87.53(13)	S1-O14-Tm1	165.8(2)
O3-Fe1-O4	81.33(13)		
O3-Fe1-O6	91.52(14)		

Table S27. Selected bond distances (Å) and bond angles (deg) in compound **12**.

Bond distance (Å)			
Tm1-O3	2.249(6)	Fe1-O1	1.969(7)
Tm1-O3	2.249(6)	Fe1-O2	1.960(7)
Tm1-O3	2.249(6)	Fe1-O2	1.960(7)
Tm1-O4	2.241(8)	Fe1-O2	1.960(7)
Tm1-O4	2.241(8)	S1-O1	1.471(7)

Tm1-O4	2.241(8)	S1-O2	1.476(7)
Tm1-O5	2.30(2)	S1-O3	1.443(6)
Fe1-O1	1.969(7)	S1-O4	1.432(8)
Fe1-O1	1.969(7)		
Bond angle (deg)			
O3-Tm1-O3	82.2(2)	O1-Fe1-O1	85.9(3)
O3-Tm1-O3	82.2(2)	O2-Fe1-O1	90.4(3)
O3-Tm1-O3	82.2(2)	O2-Fe1-O1	90.4(3)
O3-Tm1-O5	130.61(16)	O2-Fe1-O1	175.7(3)
O3-Tm1-O5	130.61(16)	O2-Fe1-O1	175.7(3)
O3-Tm1-O5	130.61(17)	O2-Fe1-O1	175.7(3)
O4-Tm1-O3	76.5(3)	O2-Fe1-O1	91.7(3)
O4-Tm1-O3	76.5(3)	O2-Fe1-O1	91.7(3)
O4-Tm1-O3	154.5(2)	O2-Fe1-O1	90.4(3)
O4-Tm1-O3	154.5(2)	O2-Fe1-O1	91.7(3)
O4-Tm1-O3	154.5(2)	O2-Fe1-O2	91.9(3)
O4-Tm1-O3	81.1(3)	O2-Fe1-O2	91.8(3)
O4-Tm1-O3	76.5(3)	O2-Fe1-O2	91.9(3)
O4-Tm1-O3	81.1(3)	O1-S1-O2	104.1(4)
O4-Tm1-O3	81.1(3)	O3-S1-O1	110.3(4)
O4-Tm1-O4	113.30(15)	O3-S1-O2	111.5(4)
O4-Tm1-O4	113.31(15)	O4-S1-O1	109.7(4)
O4-Tm1-O4	113.31(15)	O4-S1-O2	110.9(4)
O4-Tm1-O5	74.71(18)	O4-S1-O3	110.1(4)
O4-Tm1-O5	74.71(18)	S1-O1-Fe1	144.1(4)
O4-Tm1-O5	74.70(18)	S1-O2-Fe1	143.6(4)
O1-Fe1-O1	85.9(3)	S1-O3-Tm1	153.1(4)
O1-Fe1-O1	85.9(3)	S1-O4-Tm1	166.2(5)

Table S28. Selected bond distances (Å) and bond angles (deg) in compound **13**.

Bond distance (Å)			
Yb1-O3	2.240(8)	Fe1-O1	1.963(8)
Yb1-O3	2.240(8)	Fe1-O2	1.962(9)
Yb1-O3	2.240(8)	Fe1-O2	1.962(9)
Yb1-O4	2.239(10)	Fe1-O2	1.962(8)
Yb1-O4	2.239(10)	S1-O1	1.474(9)
Yb1-O4	2.239(10)	S1-O2	1.478(8)
Yb1-O5	2.30(2)	S1-O3	1.448(8)
Fe1-O1	1.963(9)	S1-O4	1.425(10)
Fe1-O1	1.963(8)		
Bond angle (deg)			
O3-Yb1-O3	82.5(3)	O1-Fe1-O1	86.1(4)
O3-Yb1-O3	82.5(3)	O2-Fe1-O1	90.1(3)

O3-Yb1-O3	82.5(3)	O2-Fe1-O1	175.7(3)
O3-Yb1-O5	130.4(2)	O2-Fe1-O1	175.7(3)
O3-Yb1-O5	130.4(2)	O2-Fe1-O1	91.7(3)
O3-Yb1-O5	130.4(2)	O2-Fe1-O1	90.1(3)
O4-Yb1-O3	76.5(3)	O2-Fe1-O1	175.7(3)
O4-Yb1-O3	154.8(3)	O2-Fe1-O1	91.7(3)
O4-Yb1-O3	76.5(3)	O2-Fe1-O1	91.7(3)
O4-Yb1-O3	154.8(3)	O2-Fe1-O1	90.1(3)
O4-Yb1-O3	81.2(3)	O2-Fe1-O2	91.9(3)
O4-Yb1-O3	154.8(3)	O2-Fe1-O2	91.9(3)
O4-Yb1-O3	81.2(3)	O2-Fe1-O2	91.9(3)
O4-Yb1-O3	76.5(3)	O1-S1-O2	104.2(5)
O4-Yb1-O3	81.2(3)	O3-S1-O1	110.3(5)
O4-Yb1-O4	113.19(18)	O3-S1-O2	111.6(5)
O4-Yb1-O4	113.19(19)	O4-S1-O1	109.4(5)
O4-Yb1-O4	113.20(18)	O4-S1-O2	110.8(5)
O4-Yb1-O5	74.6(2)	O4-S1-O3	110.4(5)
O4-Yb1-O5	74.6(2)	S1-O1-Fe1	144.9(5)
O4-Yb1-O5	74.6(2)	S1-O2-Fe1	143.6(5)
O1-Fe1-O1	86.1(4)	S1-O3-Yb1	153.0(5)
O1-Fe1-O1	86.1(4)	S1-O4-Yb1	166.5(6)

Table S29. Selected bond distances (Å) and bond angles (deg) in compound **14**.

Bond distance (Å)			
Lu1-O3	2.236(8)	Fe1-O1	1.963(8)
Lu1-O3	2.236(8)	Fe1-O2	1.967(8)
Lu1-O3	2.236(8)	Fe1-O2	1.967(8)
Lu1-O4	2.230(10)	Fe1-O2	1.967(8)
Lu1-O4	2.230(10)	S1-O1	1.475(9)
Lu1-O4	2.230(10)	S1-O2	1.480(8)
Lu1-O5	2.30(2)	S1-O3	1.446(8)
Fe1-O1	1.963(8)	S1-O4	1.429(10)
Fe1-O1	1.963(8)		
Bond angle (deg)			
O3-Lu1-O3	82.8(3)	O1-Fe1-O1	86.5(3)
O3-Lu1-O3	82.8(3)	O1-Fe1-O2	89.9(3)
O3-Lu1-O3	82.8(3)	O1-Fe1-O2	89.9(3)
O3-Lu1-O5	130.22(19)	O1-Fe1-O2	91.9(3)
O3-Lu1-O5	130.2(2)	O1-Fe1-O2	91.9(3)
O3-Lu1-O5	130.22(19)	O1-Fe1-O2	91.9(3)
O4-Lu1-O3	76.7(3)	O1-Fe1-O2	176.1(3)
O4-Lu1-O3	155.5(3)	O1-Fe1-O2	176.1(3)
O4-Lu1-O3	76.7(3)	O1-Fe1-O2	89.9(3)

O4-Lu1-O3	155.5(3)	O1-Fe1-O2	176.1(3)
O4-Lu1-O3	81.5(3)	O2-Fe1-O2	91.7(3)
O4-Lu1-O3	155.5(3)	O2-Fe1-O2	91.7(3)
O4-Lu1-O3	81.5(3)	O2-Fe1-O2	91.7(3)
O4-Lu1-O3	76.7(3)	O1-S1-O2	103.9(5)
O4-Lu1-O3	81.5(3)	O3-S1-O1	110.3(5)
O4-Lu1-O4	112.73(19)	O3-S1-O2	111.7(4)
O4-Lu1-O4	112.73(19)	O4-S1-O1	109.2(5)
O4-Lu1-O4	112.73(19)	O4-S1-O2	111.4(5)
O4-Lu1-O5	74.0(2)	O4-S1-O3	110.2(5)
O4-Lu1-O5	74.0(2)	S1-O1-Fe1	145.3(5)
O4-Lu1-O5	74.0(2)	S1-O2-Fe1	143.5(5)
O1-Fe1-O1	86.5(3)	S1-O3-Lu1	153.0(5)
O1-Fe1-O1	86.5(3)	S1-O4-Lu1	166.7(6)

Table S30: The calculated bond valence sums for Ln, Fe, S, and O in compounds **1-11**.

Atom	1	2	3	4	5	6	7	8	9	10	11
Ln(1)	2.63	3.18	3.15	3.26	3.39	3.36	3.10	3.06	3.32	3.25	2.99
Fe(1)	2.99	3.05	3.08	3.17	3.15	3.14	3.16	3.20	3.21	3.24	3.25
S(1)	6.03	6.07	6.09	6.25	6.13	6.10	6.11	6.16	6.16	6.15	6.17
S(2)	6.05	6.04	6.04	6.23	6.09	6.08	6.07	6.11	6.07	6.09	6.11
S(3)	5.99	6.06	6.10	6.17	6.05	6.04	6.07	6.06	6.06	6.02	6.06
O(1)	1.91	1.95	1.97	1.99	1.97	1.96	1.98	1.99	2.03	2.00	2.04
O(2)	1.94	1.93	1.95	2.00	1.98	1.99	2.01	1.99	1.99	2.02	2.00
O(3)	1.95	1.96	1.96	2.01	1.98	1.97	1.98	1.99	1.98	1.99	1.99
O(4)	1.93	1.96	2.00	2.03	1.99	1.97	1.97	1.98	2.01	1.97	1.99
O(5)	1.92	1.96	1.95	2.05	1.99	1.99	2.00	2.00	1.98	1.99	1.94
O(6)	1.88	1.96	1.98	2.01	1.99	1.96	1.98	2.02	1.98	2.00	2.01
O(7)	1.94	1.99	2.01	1.99	2.01	2.00	1.98	1.96	1.99	1.98	1.94
O(8)	1.94	1.95	2.04	2.06	1.98	1.98	1.99	1.97	1.86	1.97	2.04
O(9)	1.91	1.95	0.38	1.99	2.01	1.99	1.95	0.35	1.99	1.97	1.96
O(10)	1.94	2.00	0.34	2.03	1.99	1.99	2.03	1.96	1.98	0.39	1.93
O(11)	1.88	2.01	1.95	2.06	0.41	0.40	1.95	1.96	0.38	1.98	0.34
O(12)	1.95	2.03	1.96	0.39	2.09	0.38	0.37	0.36	2.07	0.37	2.03
O(13)	0.351	0.35	1.97	0.37	0.39	2.07	2.00	2.04	2.06	2.02	0.34
O(14)	0.291	0.39	1.98	2.10	2.05	2.04	0.35	2.01	0.39	2.08	2.00

Table S31: The calculated bond valence sums for Ln, Fe, S, and O in compounds **12-14**.

Atom	12	13	14
Ln(1)	3.25	3.26	3.11
Fe(1)	3.44	3.46	3.44

S(1)	6.32	6.31	6.28
O(1)	2.08	2.08	2.07
O(2)	2.07	2.06	2.05
O(3)	2.09	2.08	2.11
O(4)	2.16	2.19	2.10
O(5)	0.41	0.40	0.41

Table S32. Direction and magnitude of the dipole moments in compounds **13** and **14**.

	Dipole Moments (Debye)			
Compound 14	X	Y	Z	Total
LuO_7	0	0	1.80	1.80
FeO_6	-0.31	0.15	0.22	0.41
SO_4	-1.38	-0.95	0.50	1.75
Compound 13	X	Y	Z	Total
YbO_7	0	0	1.83	1.83
FeO_6	-0.33	0.14	0.21	0.40
SO_4	-1.29	-1.02	0.52	1.73

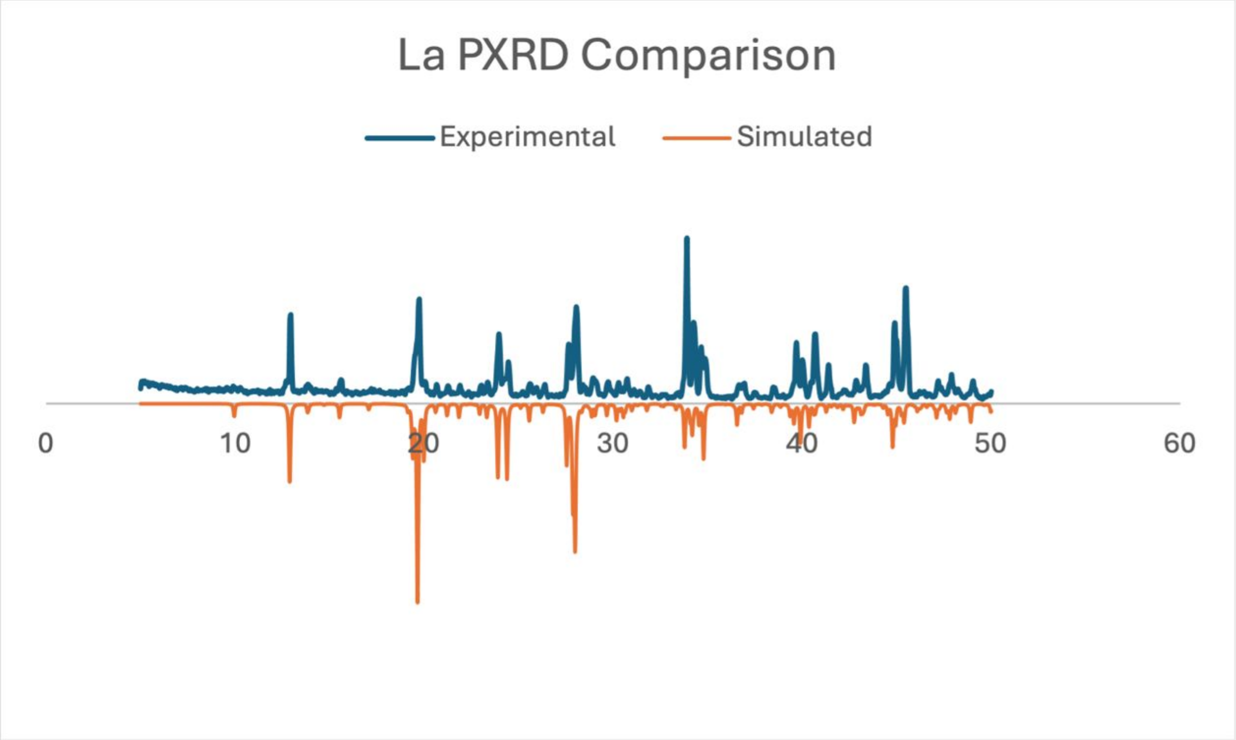

Figure S1: The powder pattern for compound **1.**

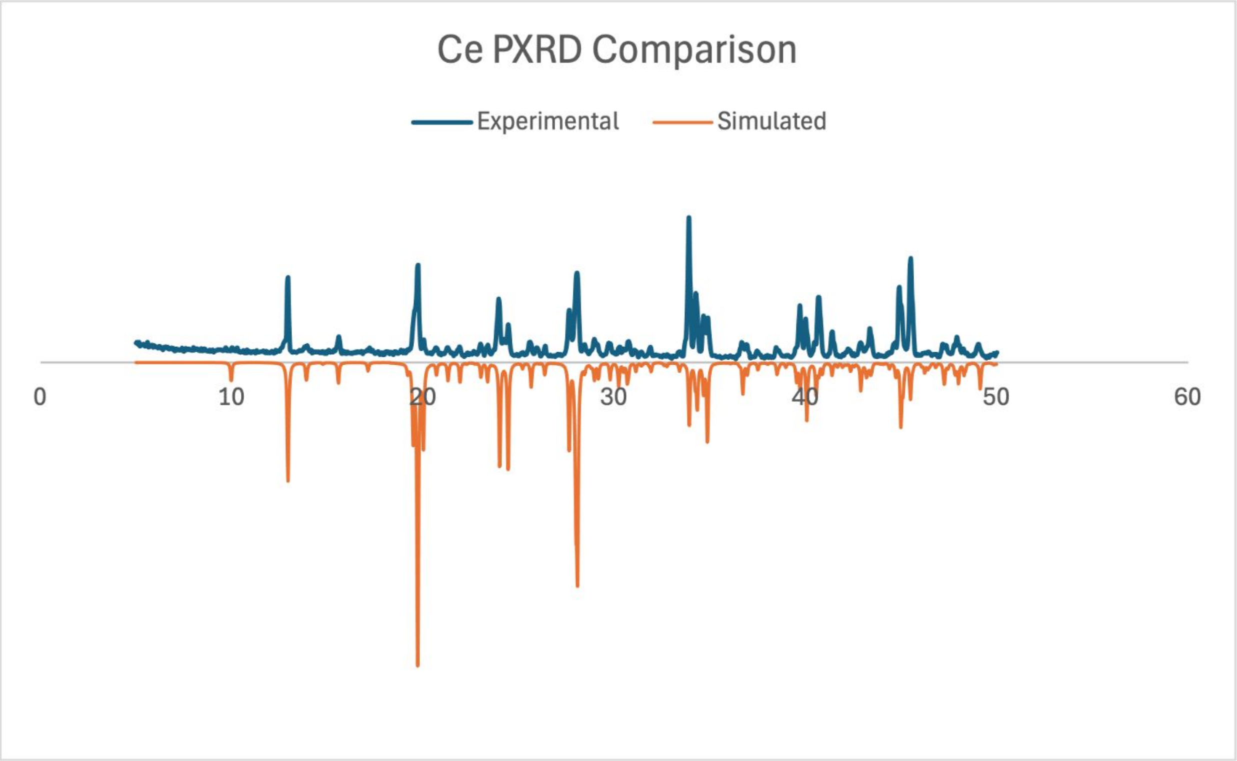

Figure S2: The powder pattern for compound **2.**

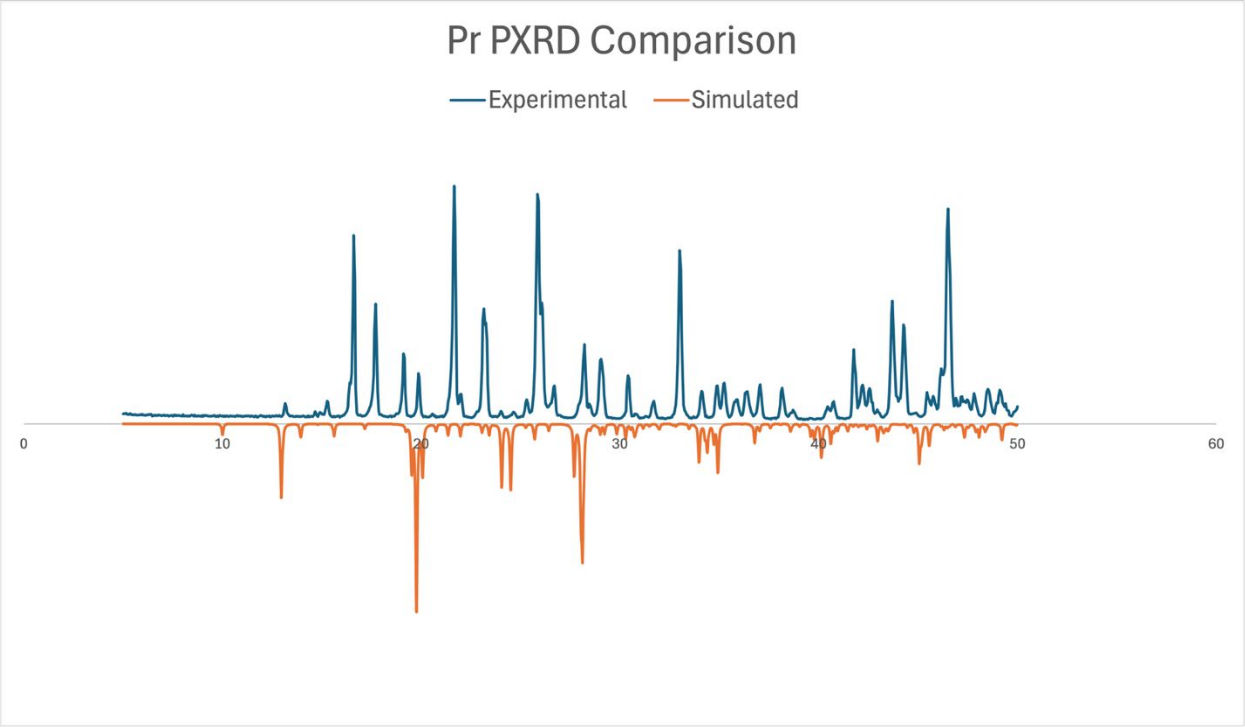

Figure S3: The powder pattern for compound **3.**

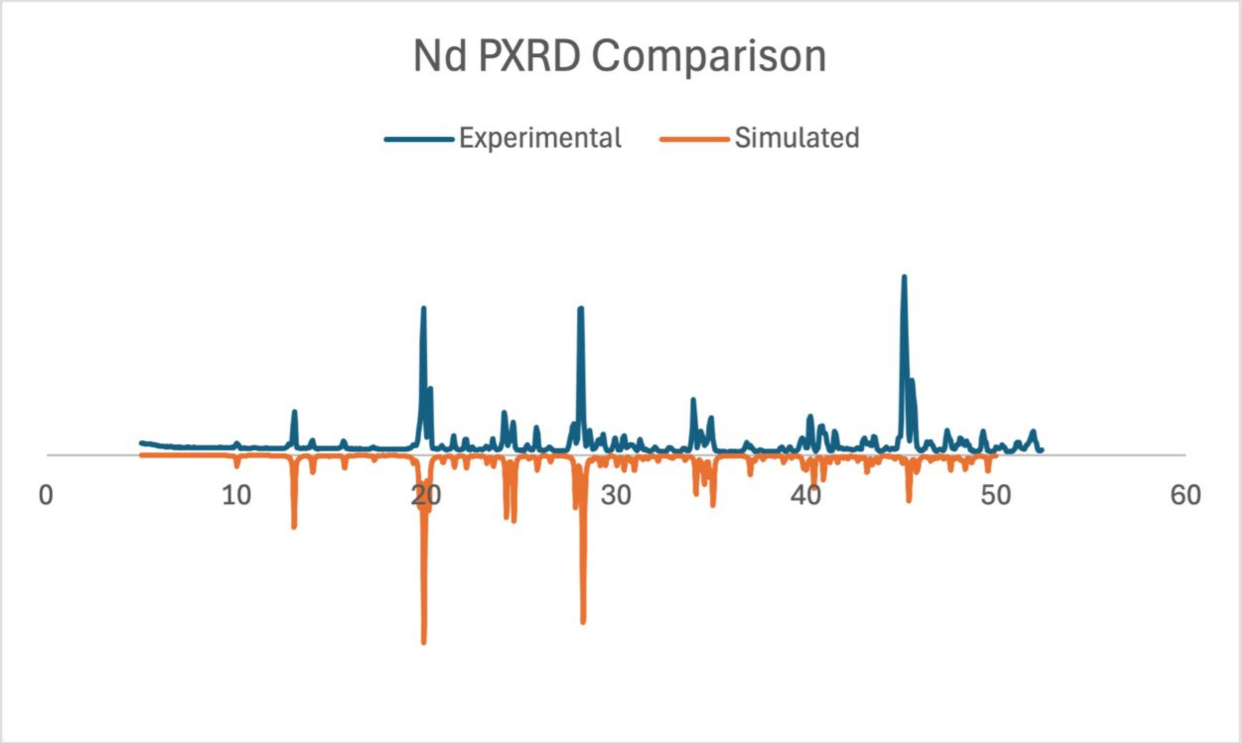

Figure S4: The powder pattern for compound **4.**

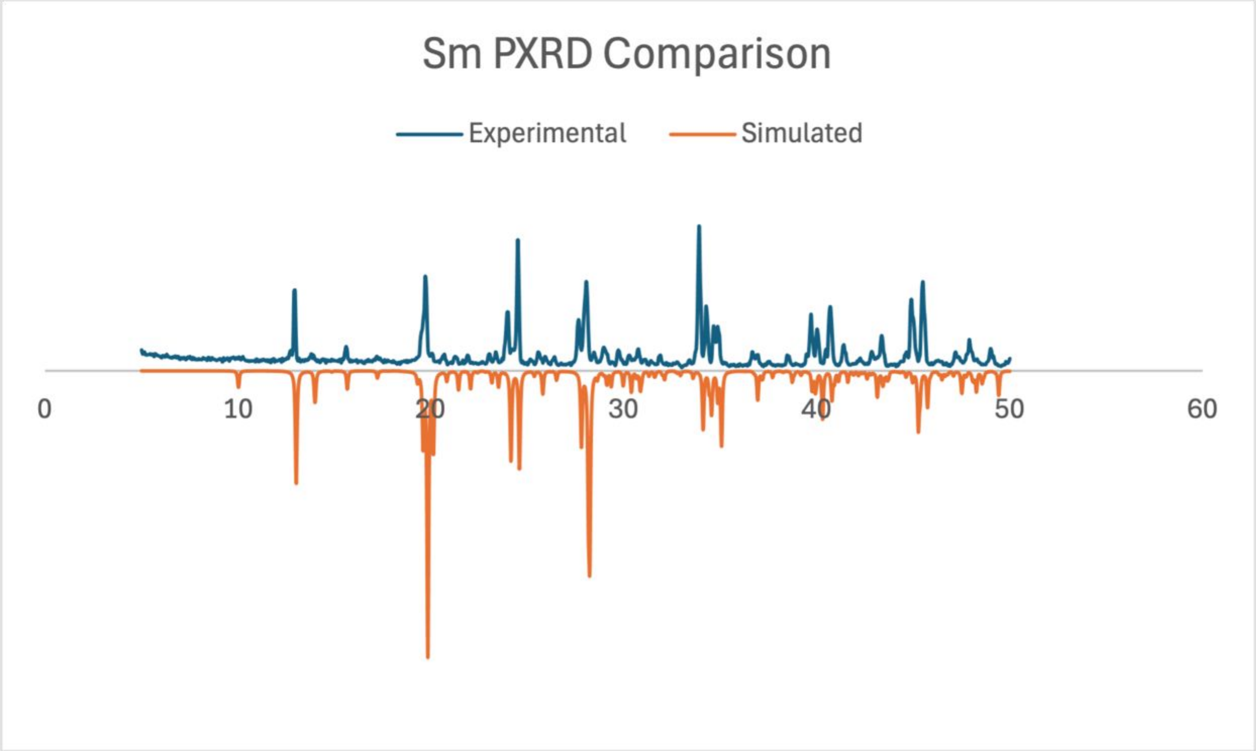

Figure S5: The powder pattern for compound **5.**

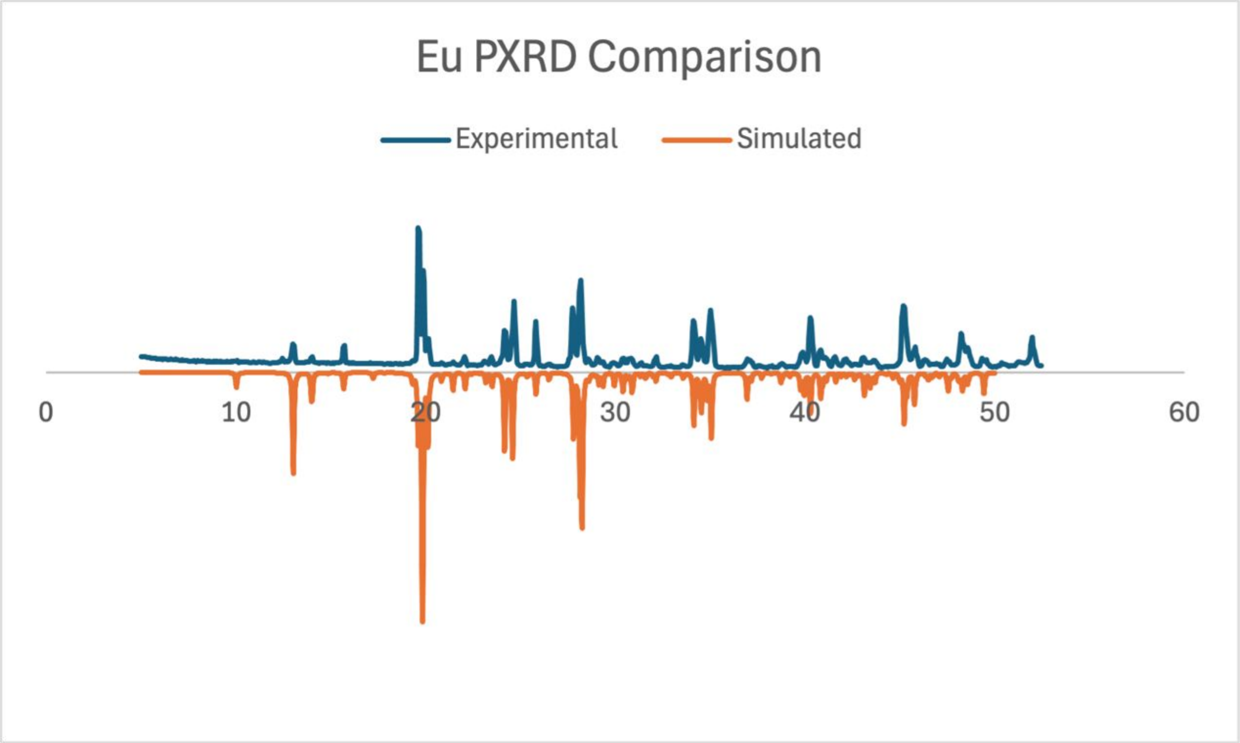

Figure S6: The powder pattern for compound **6.**

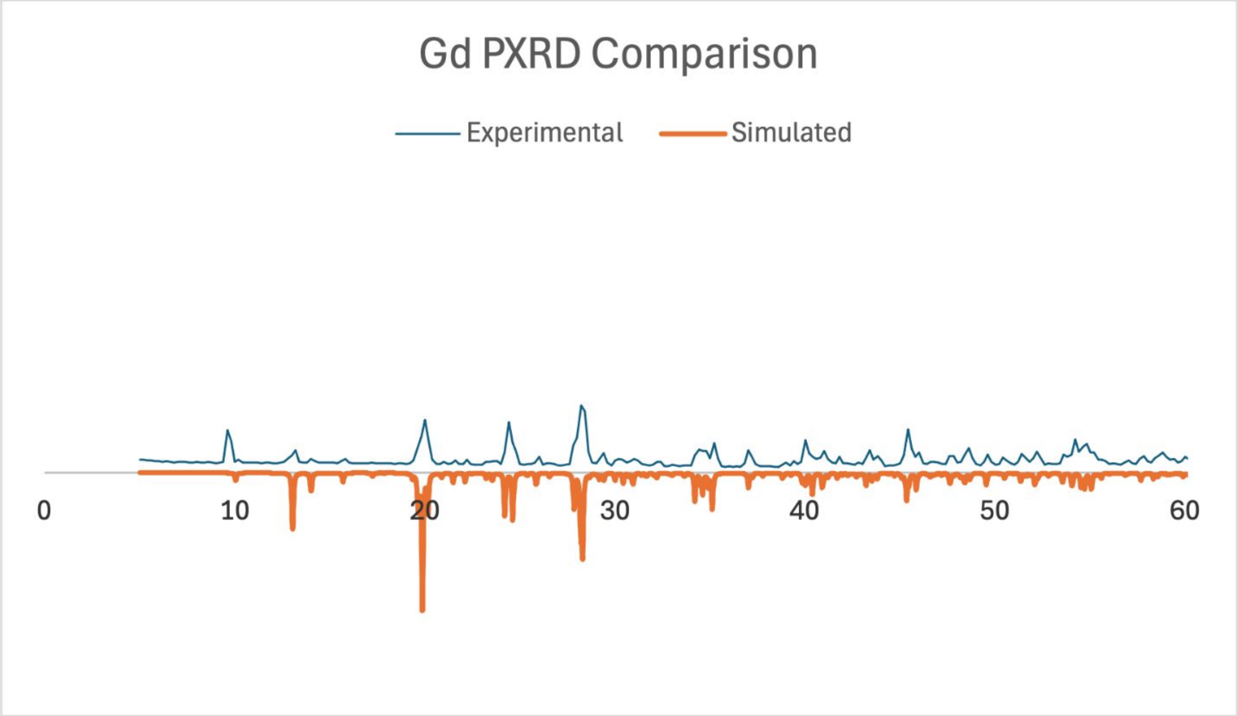

Figure S7: The powder pattern for compound **7.**

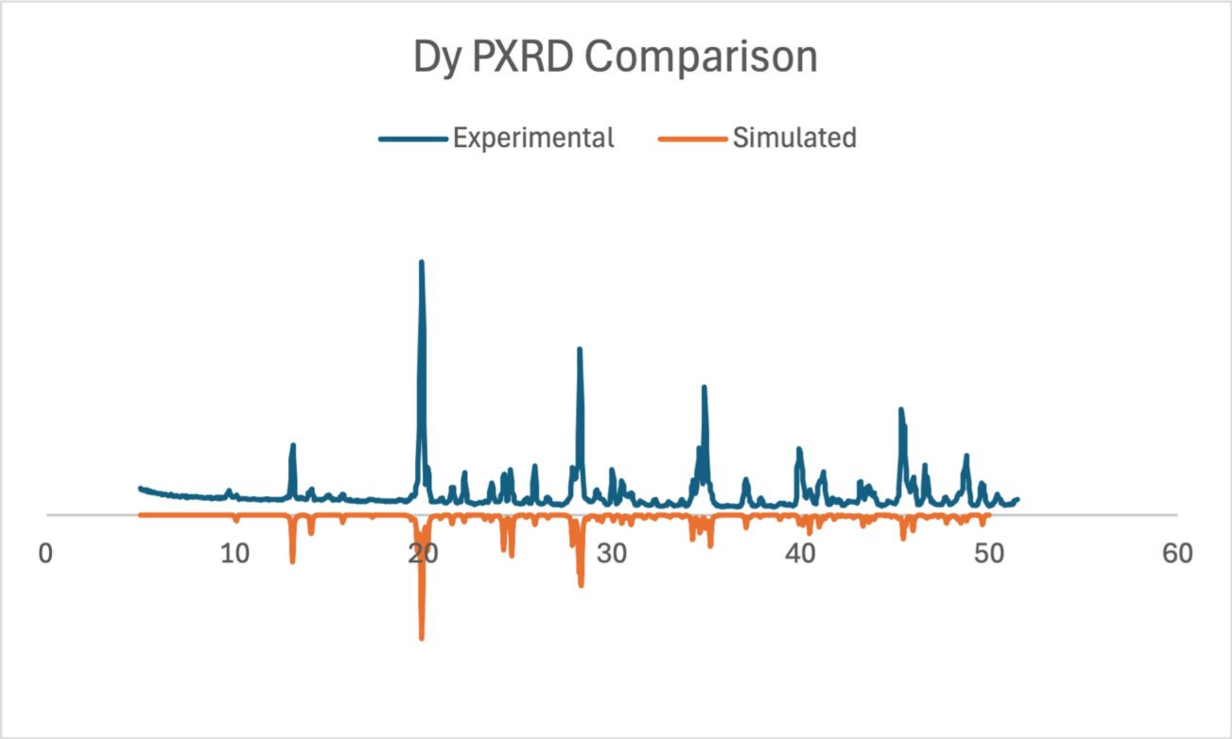

Figure S8: The powder pattern for compound **8.**

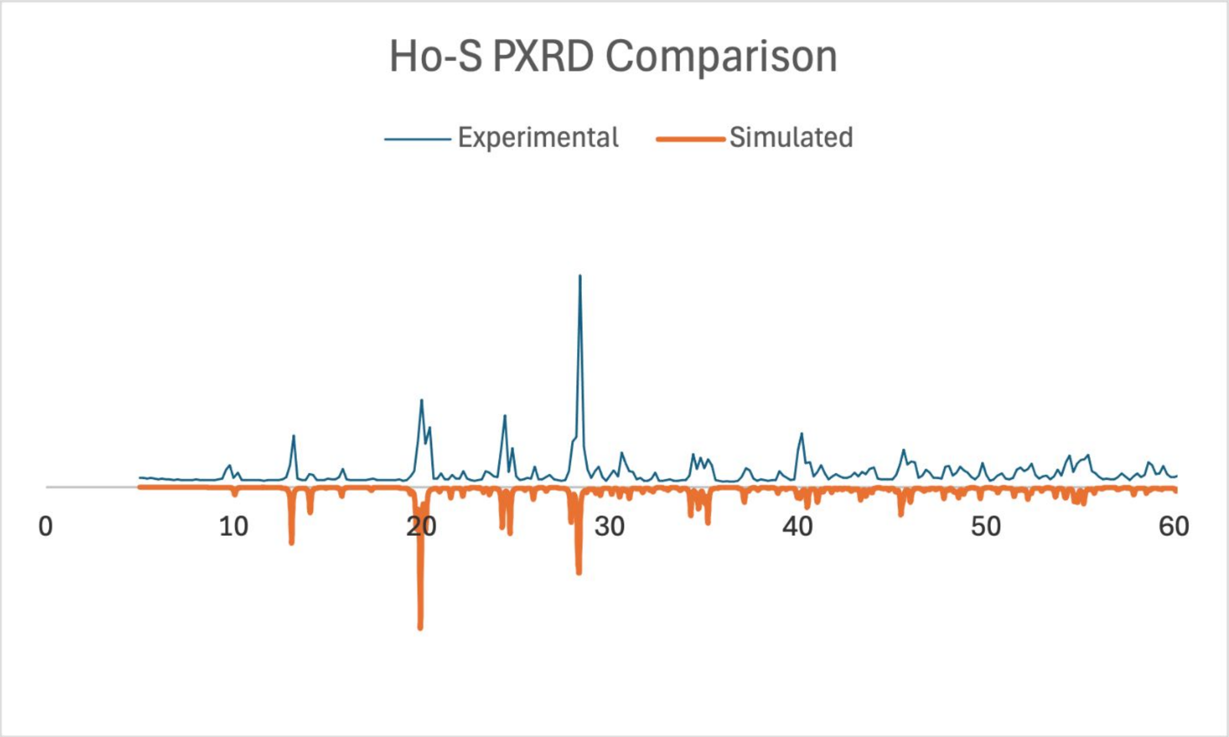

Figure S9: The powder pattern for compound **9.**

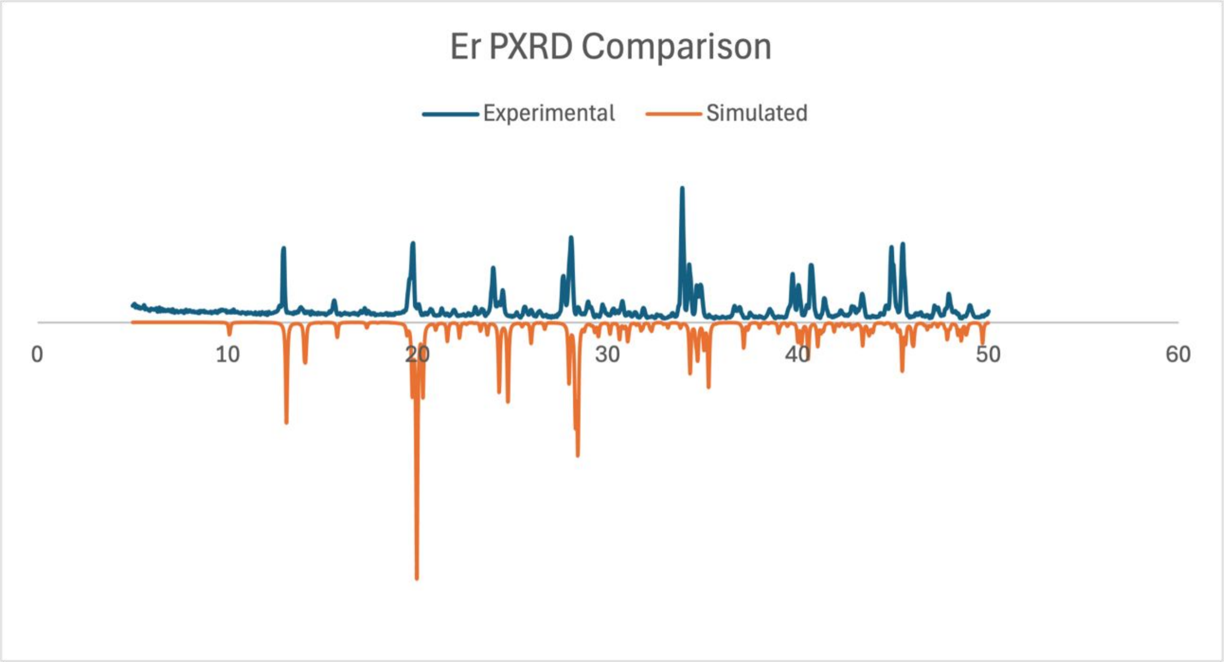

Figure S10: The powder pattern for compound **10.**

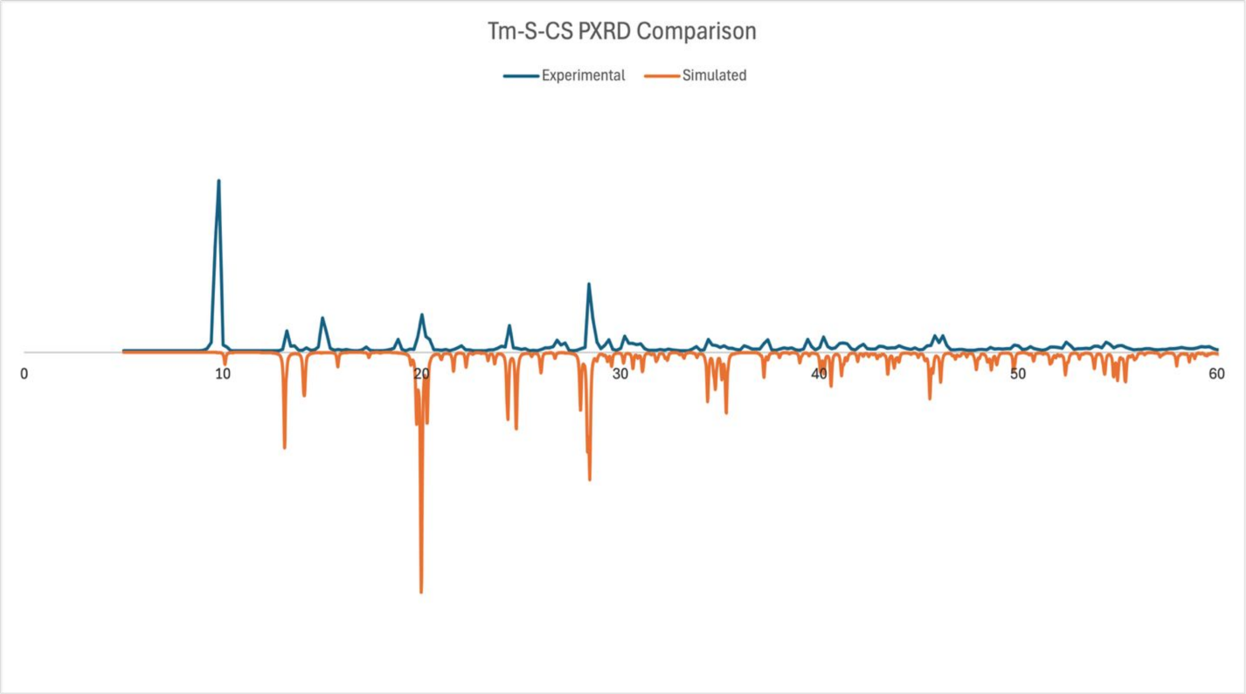

Figure S11: The powder pattern for compound **11.**

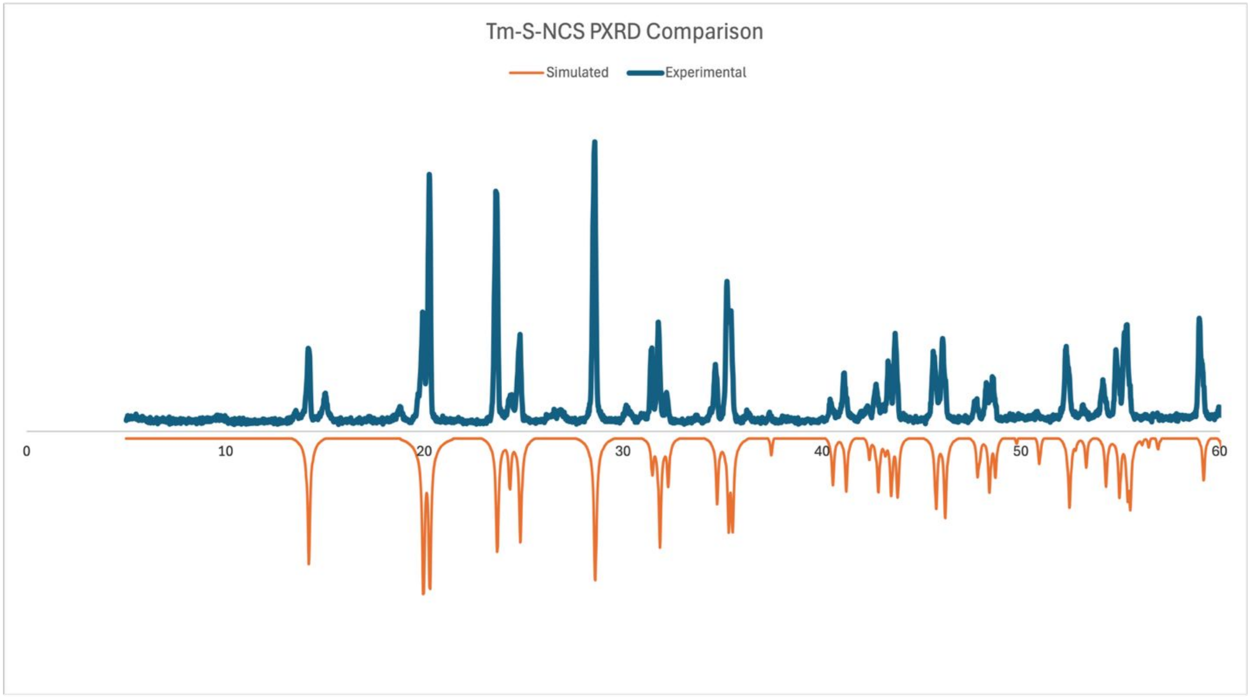

Figure S12: The powder pattern for compound **12.**

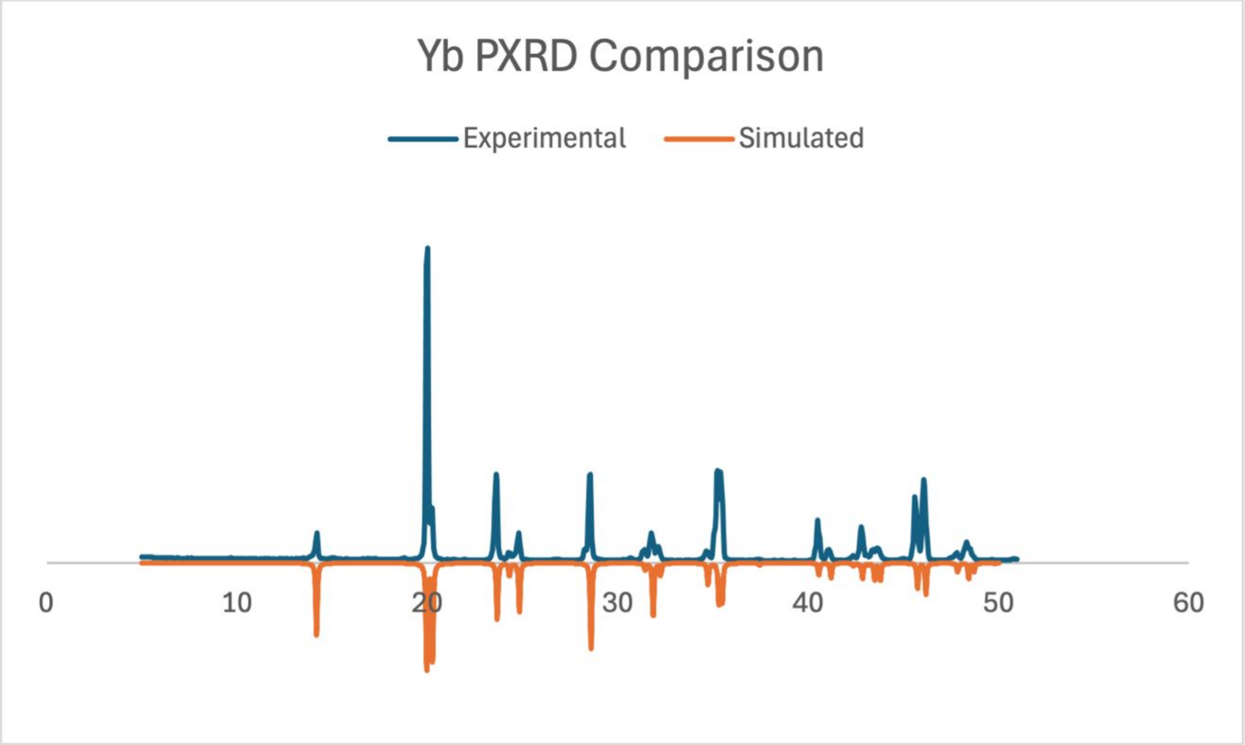

Figure S13: The powder pattern for compound **13.**

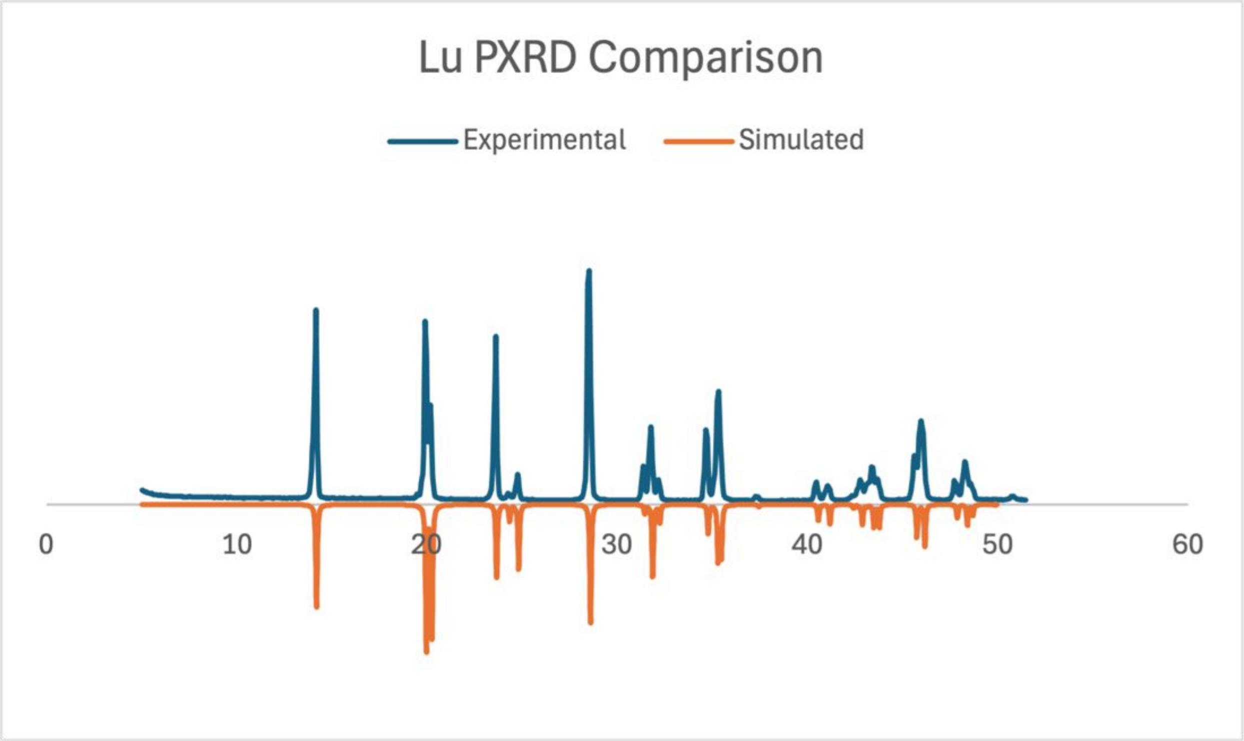

Figure S14: The powder pattern for compound **14.**

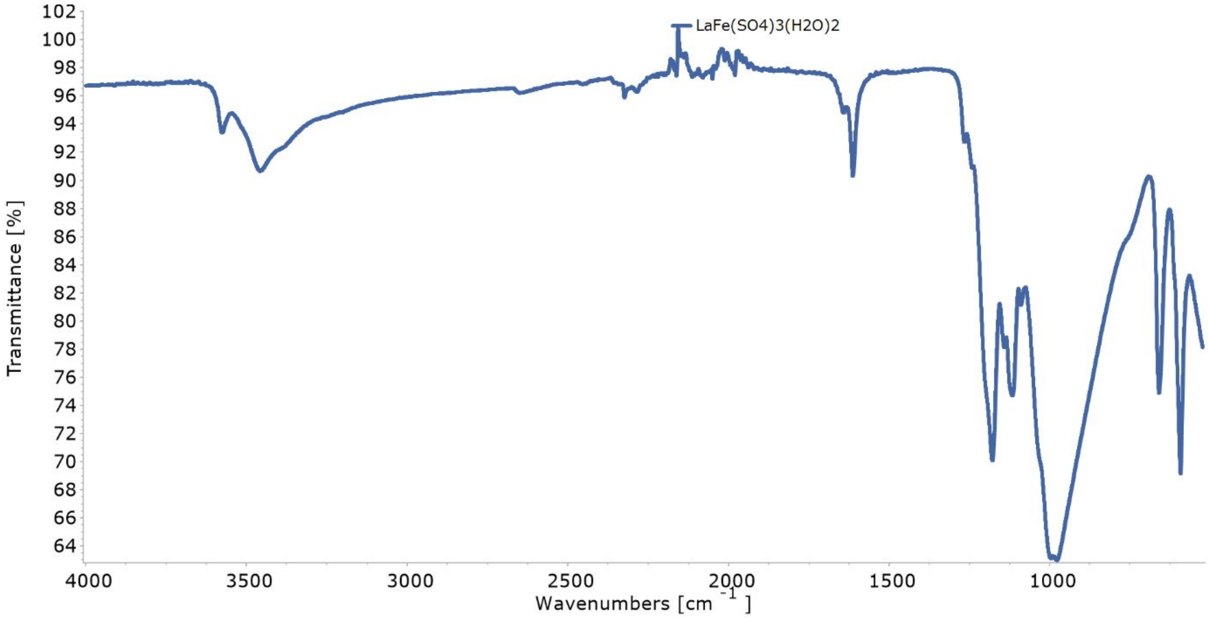

Figure S15: The FTIR Spectrum for compound **1.**

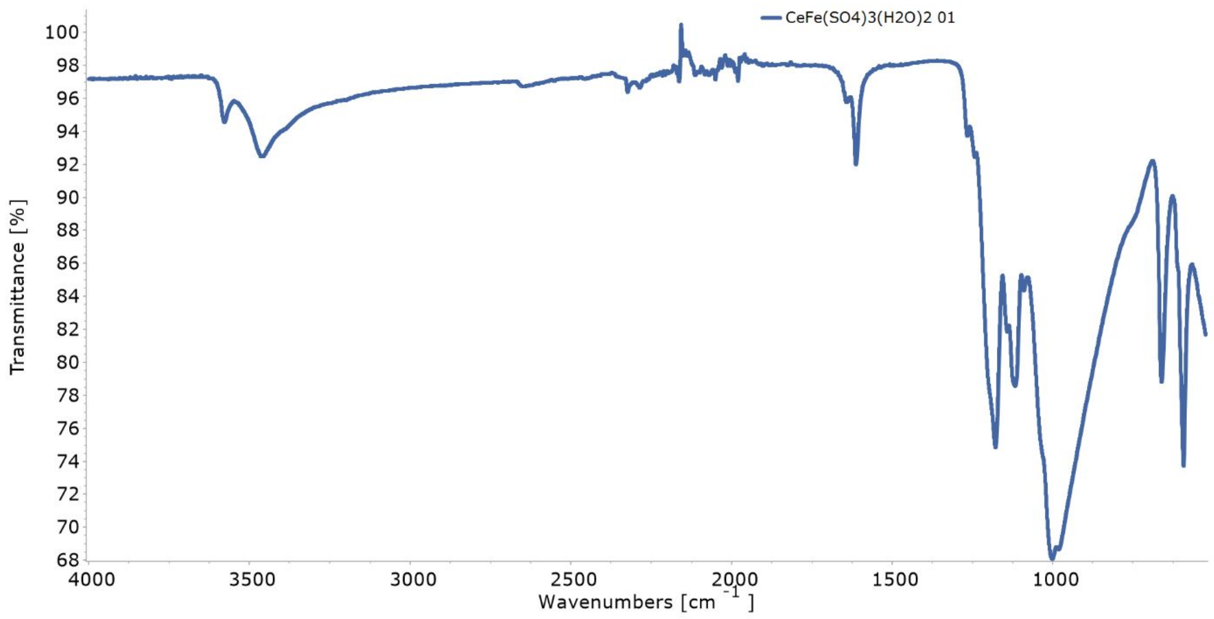

Figure S16: The FTIR Spectrum for compound **2.**

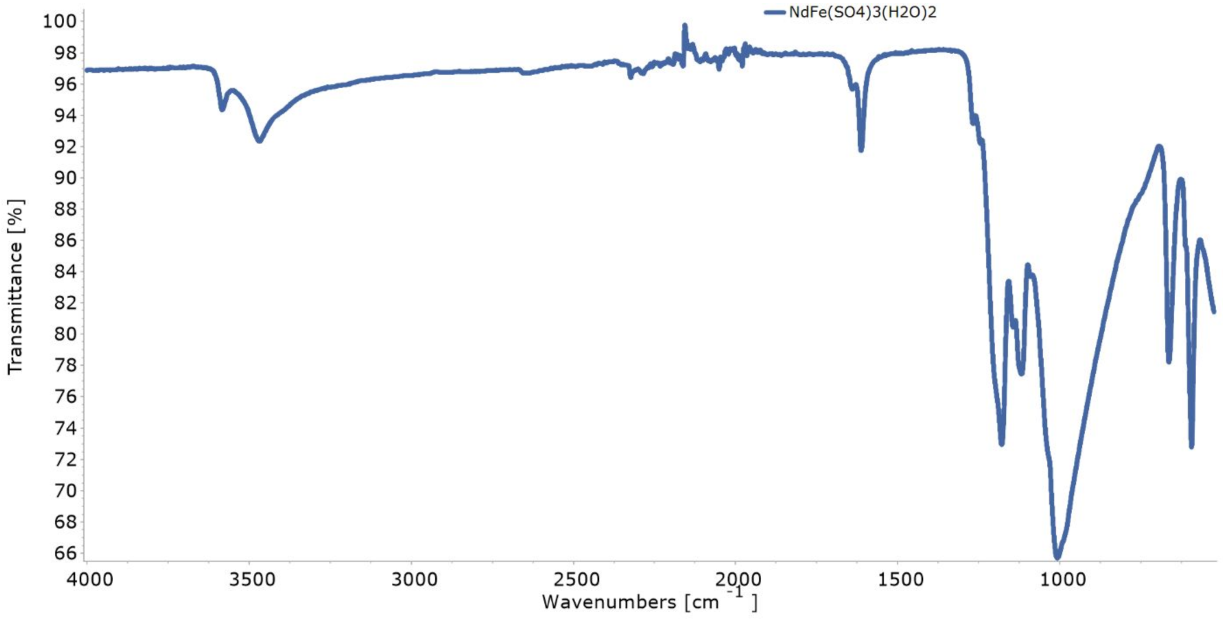

Figure S17: The FTIR Spectrum for compound **4.**

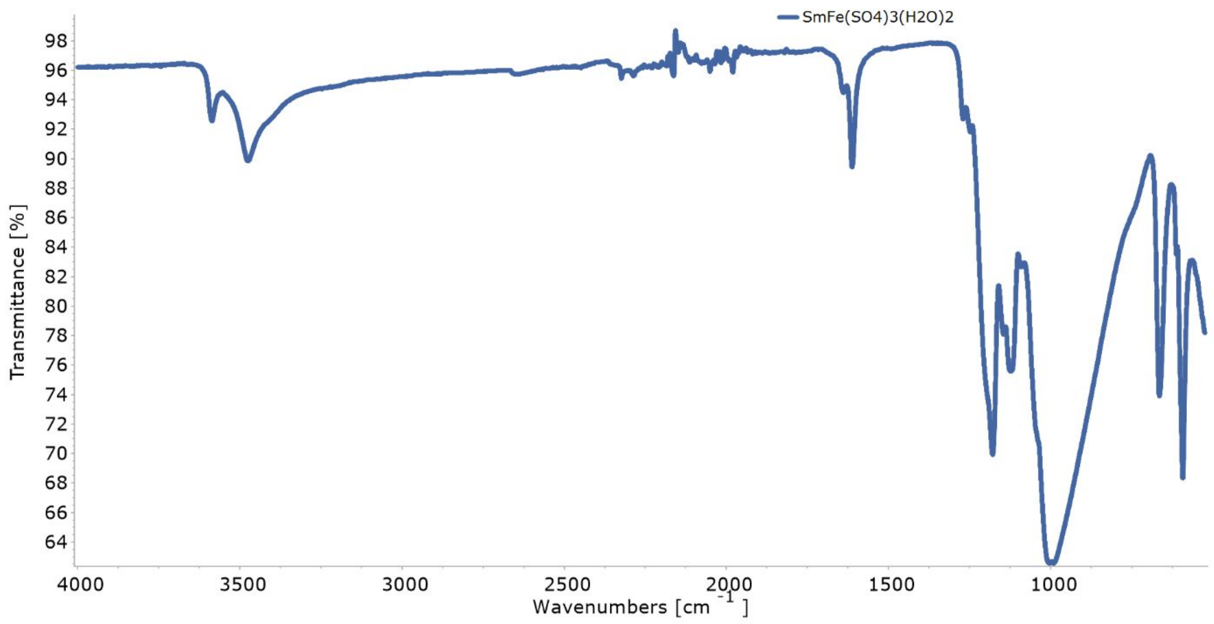

Figure S18: The FTIR Spectrum for compound **5.**

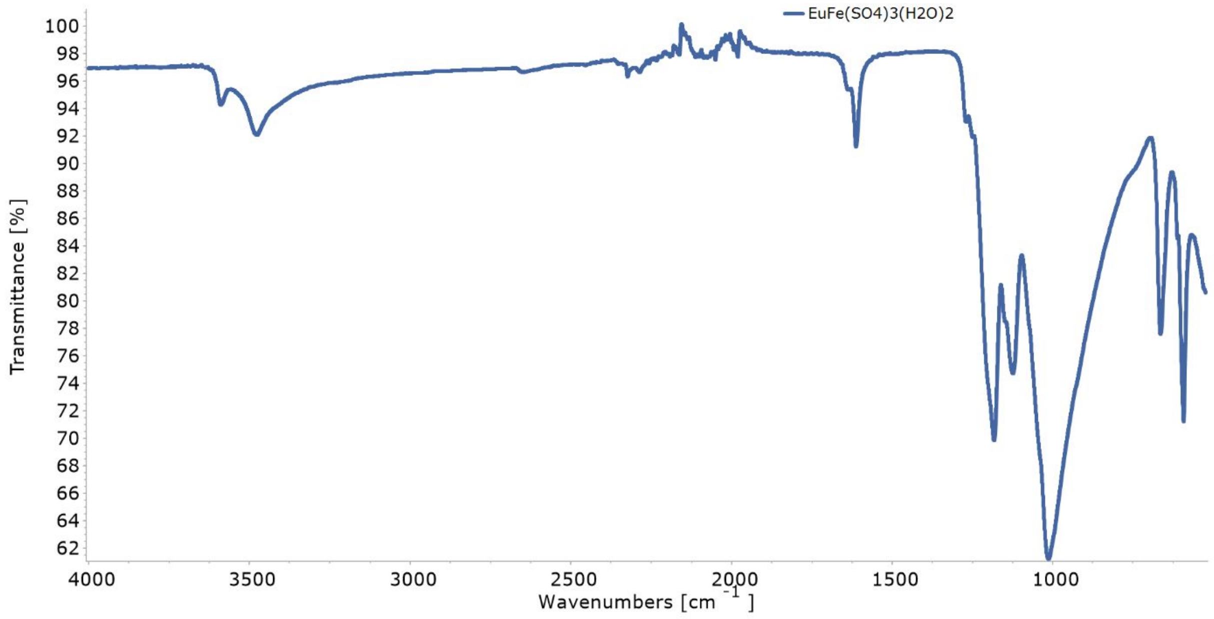

Figure S19: The FTIR Spectrum for compound **6.**

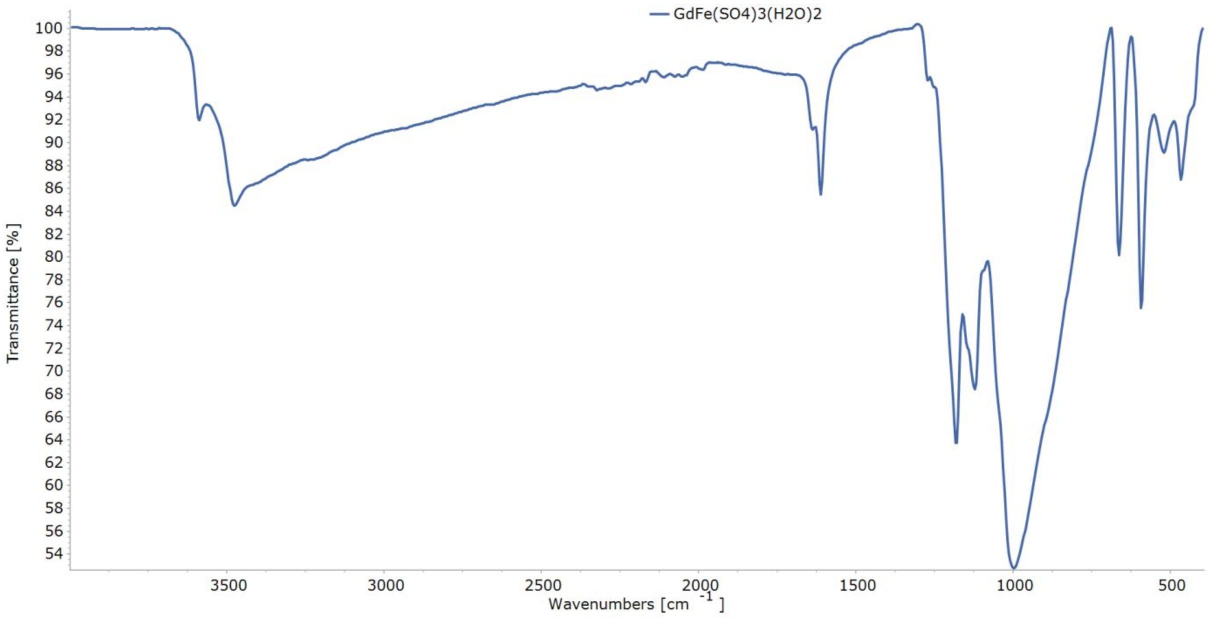

Figure S20: The FTIR Spectrum for compound **7.**

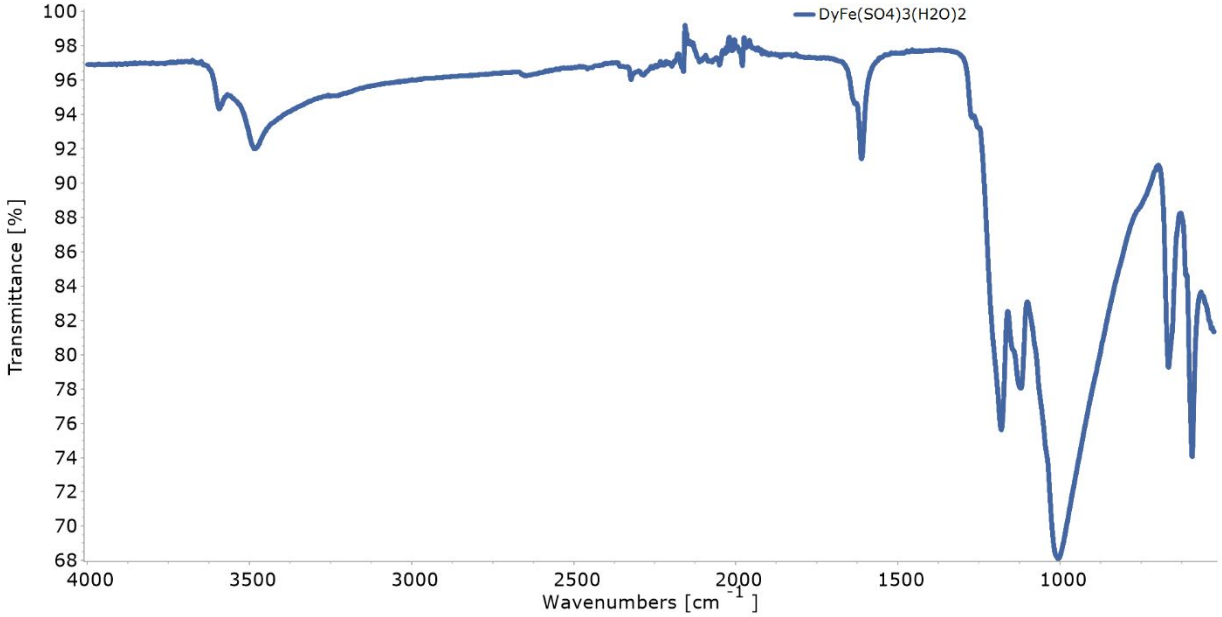

Figure S21: The FTIR Spectrum for compound **8.**

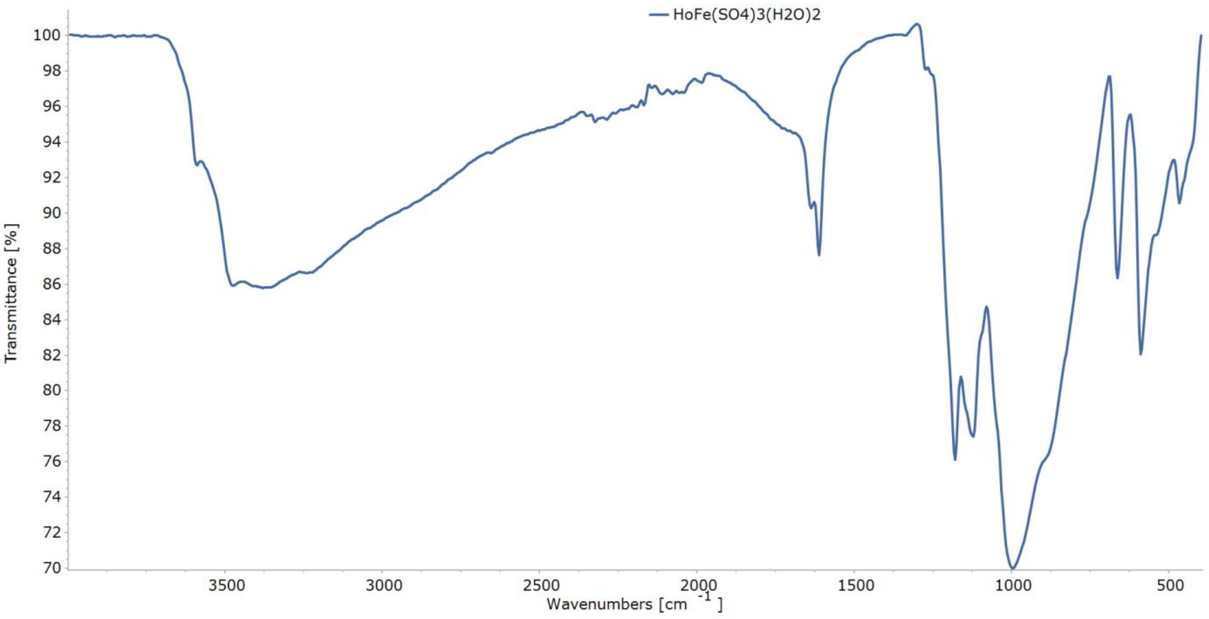

Figure S22: The FTIR Spectrum for compound **9.,**

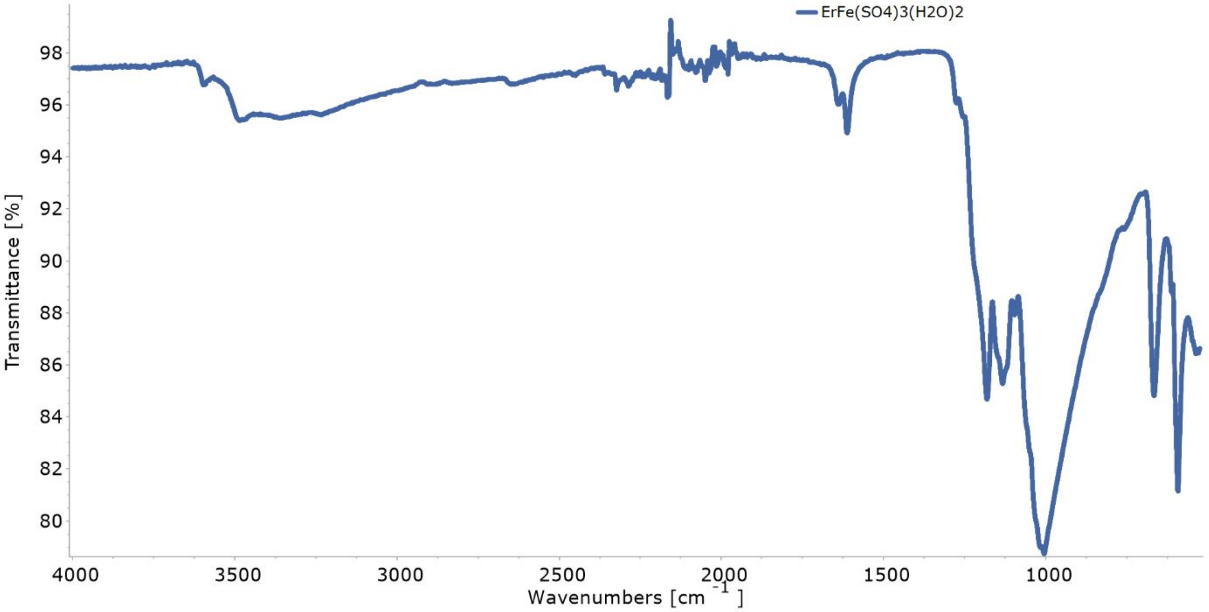

Figure S23: The FTIR Spectrum for compound **10.**

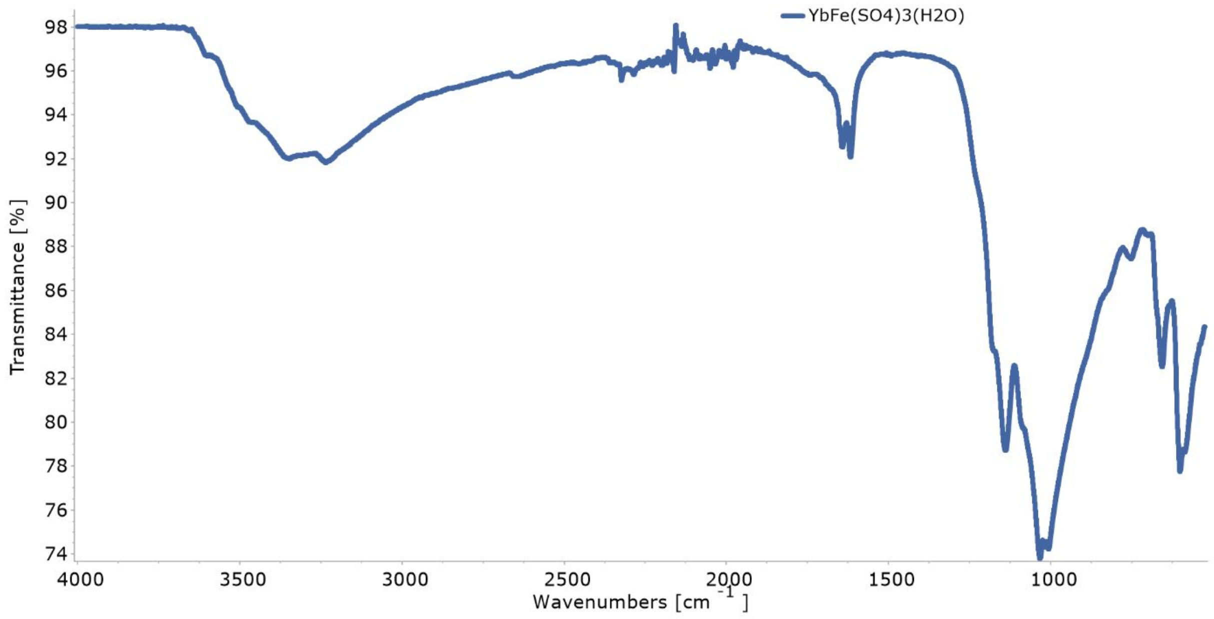

Figure S24: The FTIR Spectrum for compound **13.**

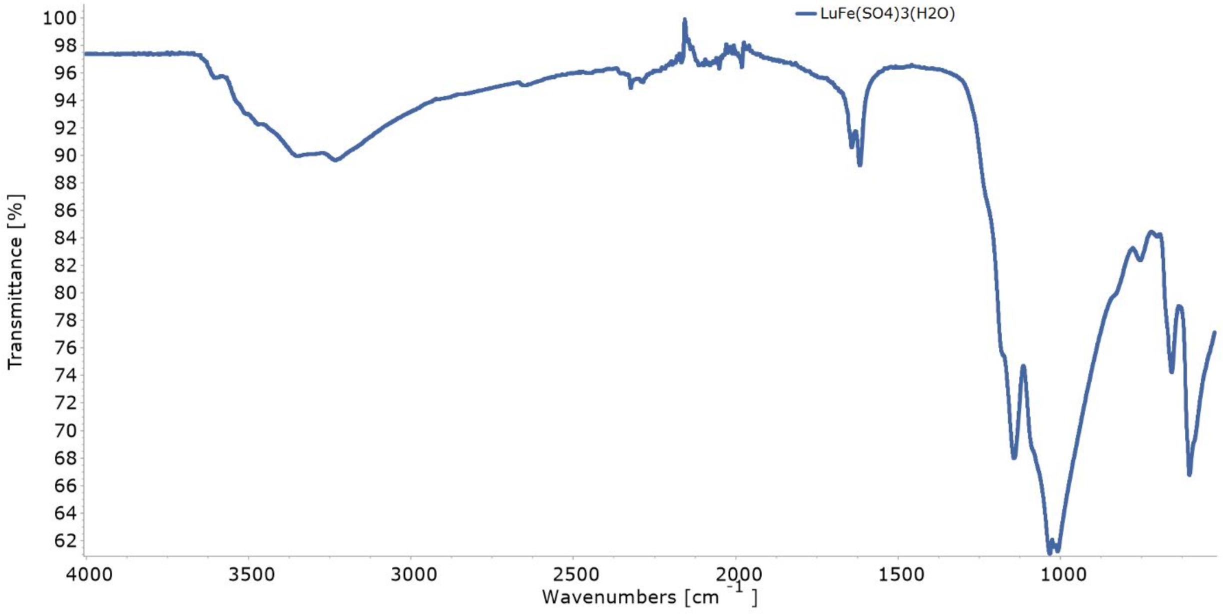

Figure S25: The FTIR Spectrum for compound **14.**

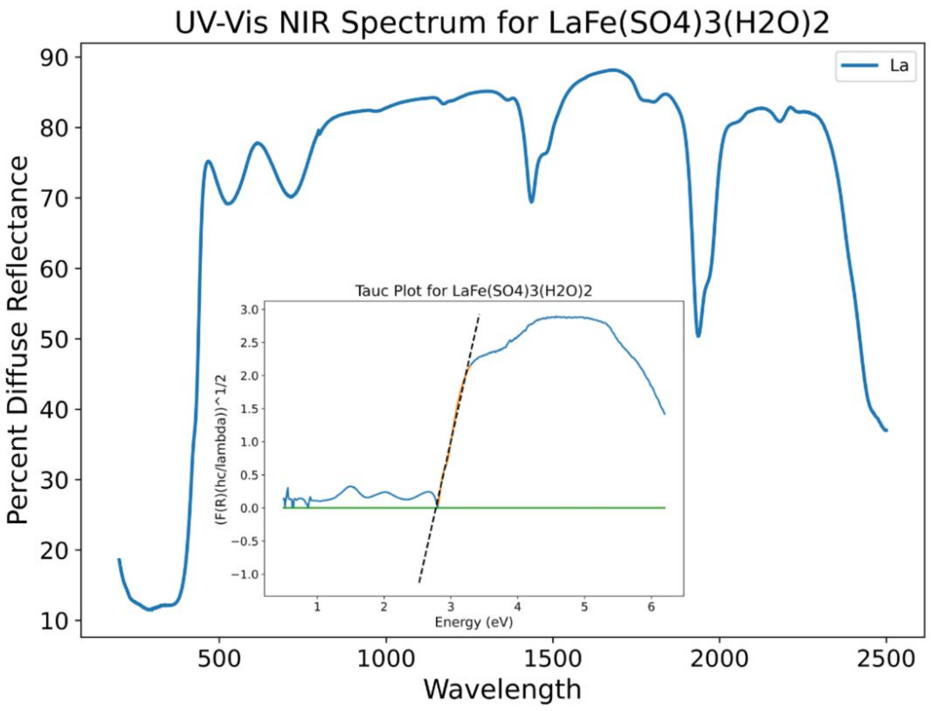

Figure S26: The UV-Vis NIR spectrum and Tauc plot for compound **1.**

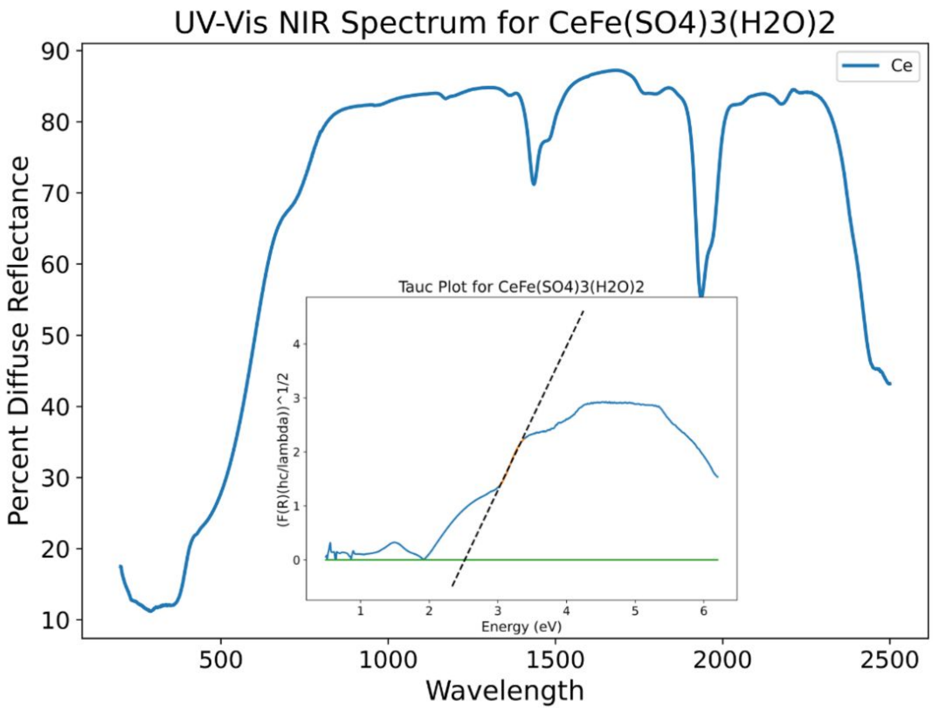

Figure S27: The UV-Vis NIR spectrum and Tauc plot for compound **2.**

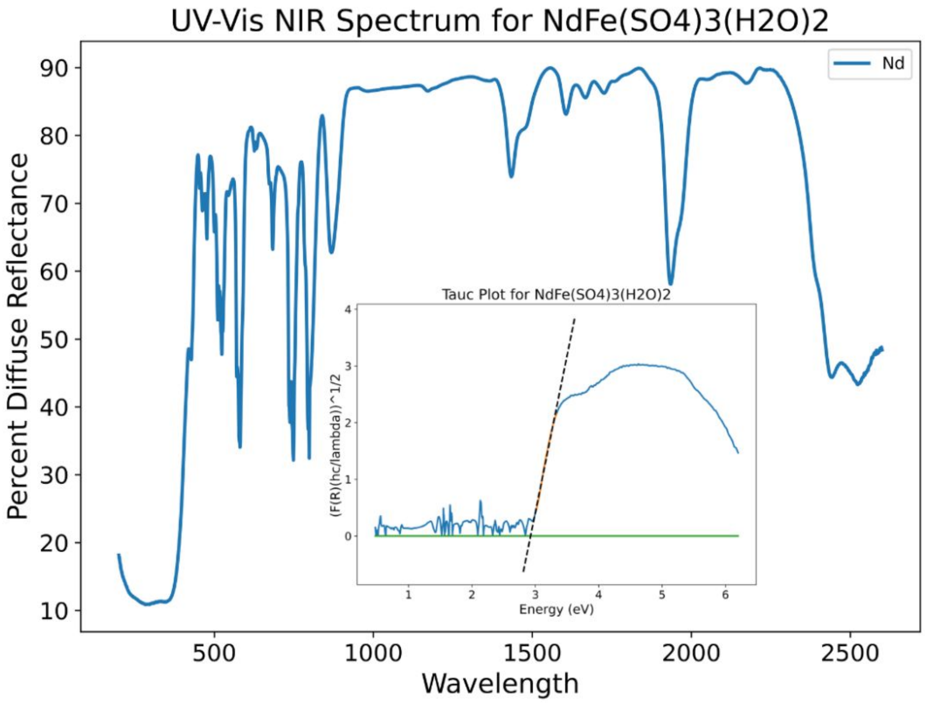

Figure S28: The UV-Vis NIR spectrum and Tauc plot for compound **4.**

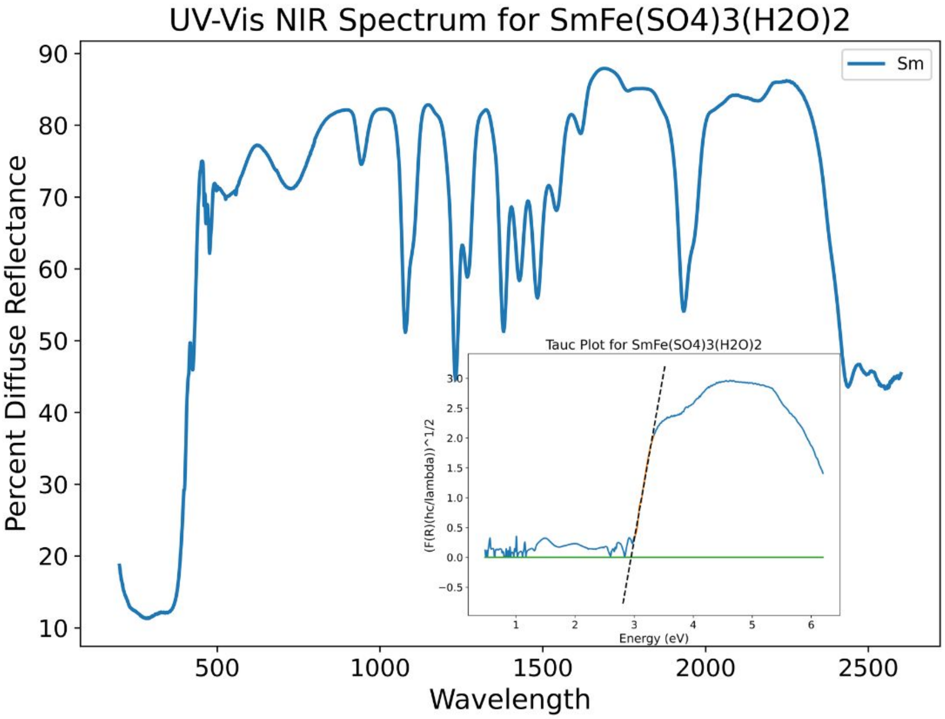

Figure S29: The UV-Vis NIR spectrum and Tauc plot for compound **5.**

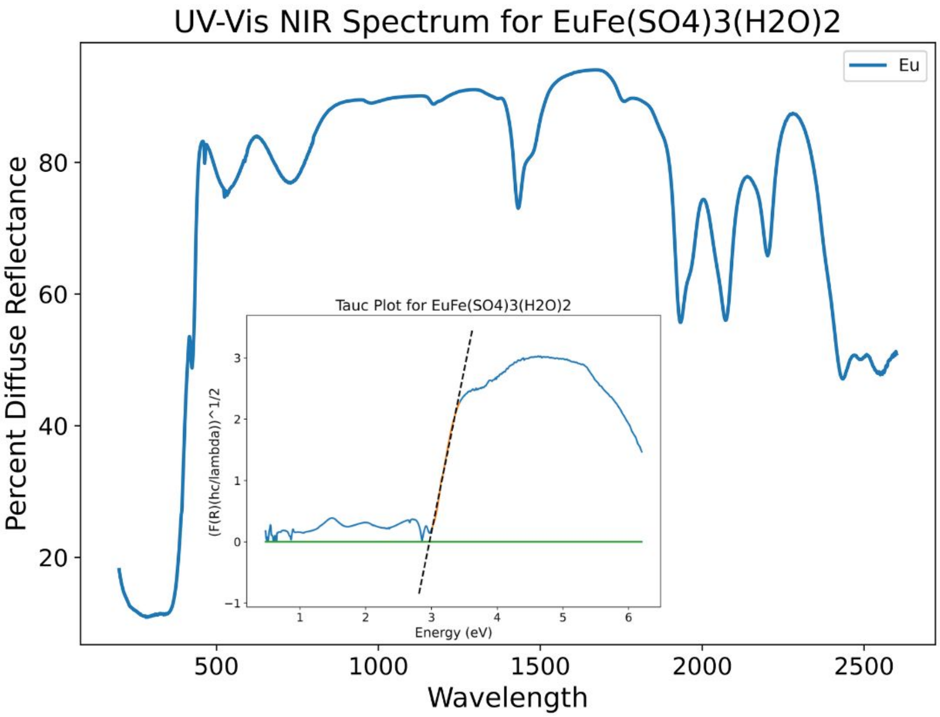

Figure S30: The UV-Vis NIR spectrum and Tauc plot for compound **6.**

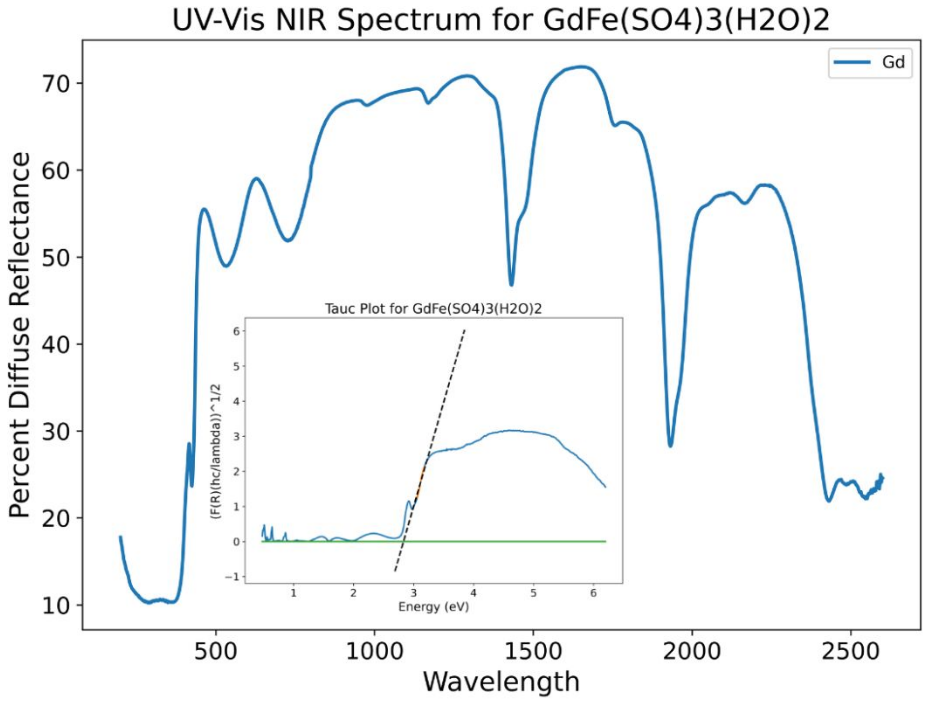

Figure S31: The UV-Vis NIR spectrum and Tauc plot for compound **7.**

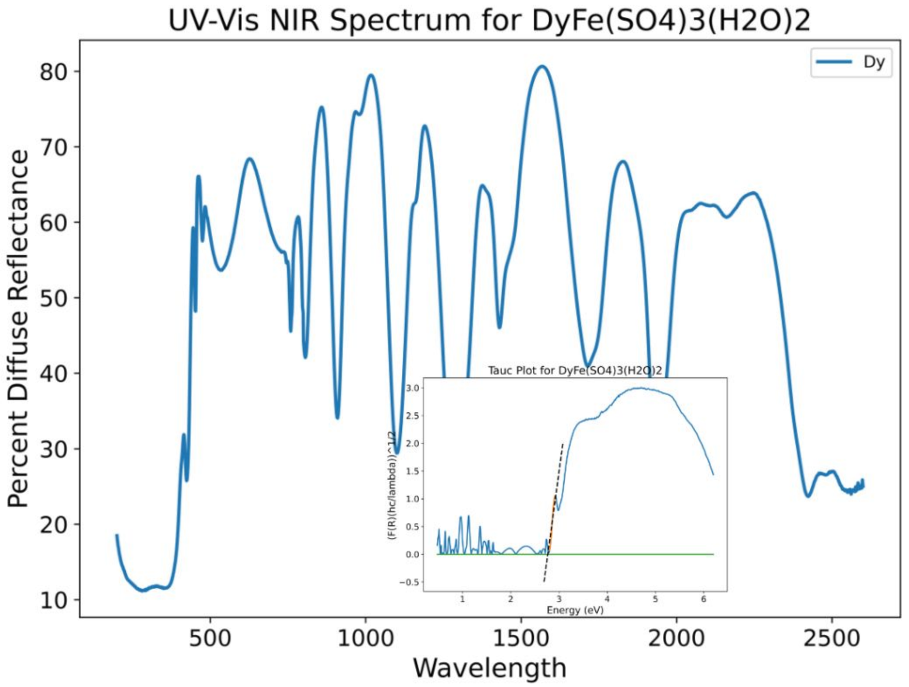

Figure S32: The UV-Vis NIR spectrum and Tauc plot for compound **8.**

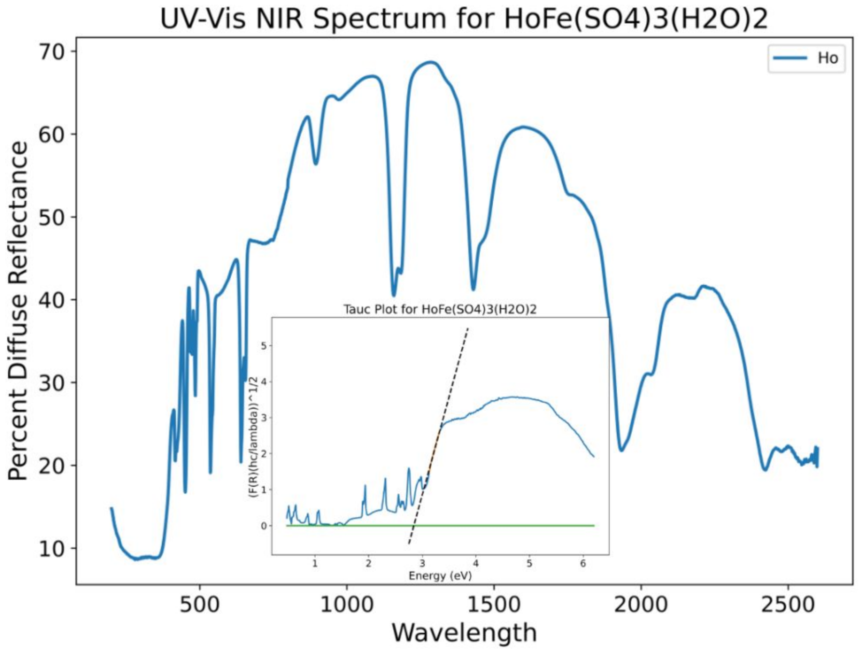

Figure S33: The UV-Vis NIR spectrum and Tauc plot for compound **9.**

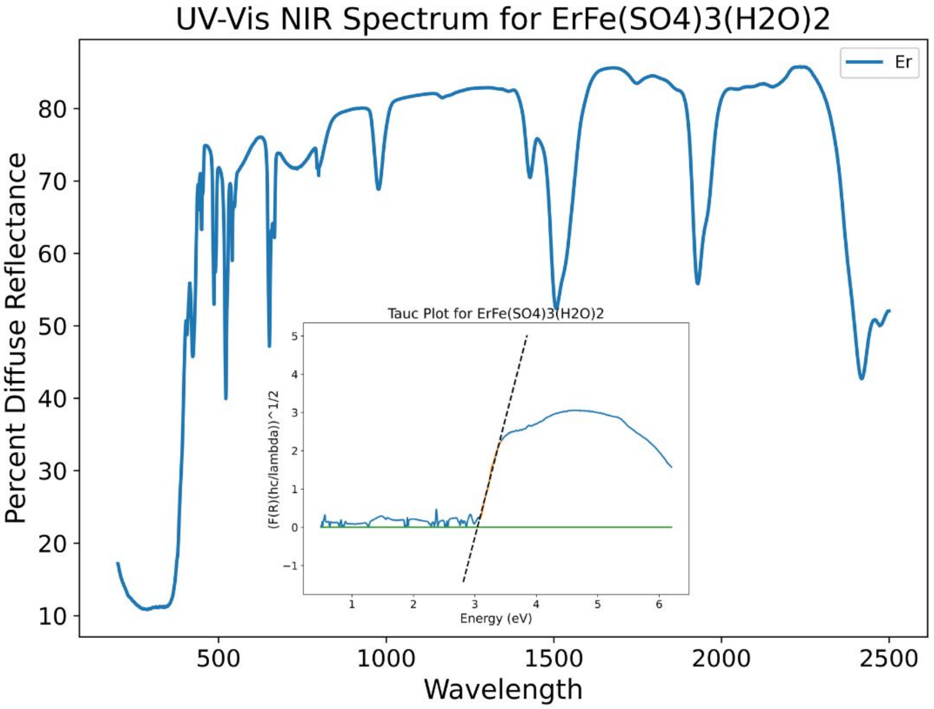

Figure S34: The UV-Vis NIR spectrum and Tauc plot for compound **10.**

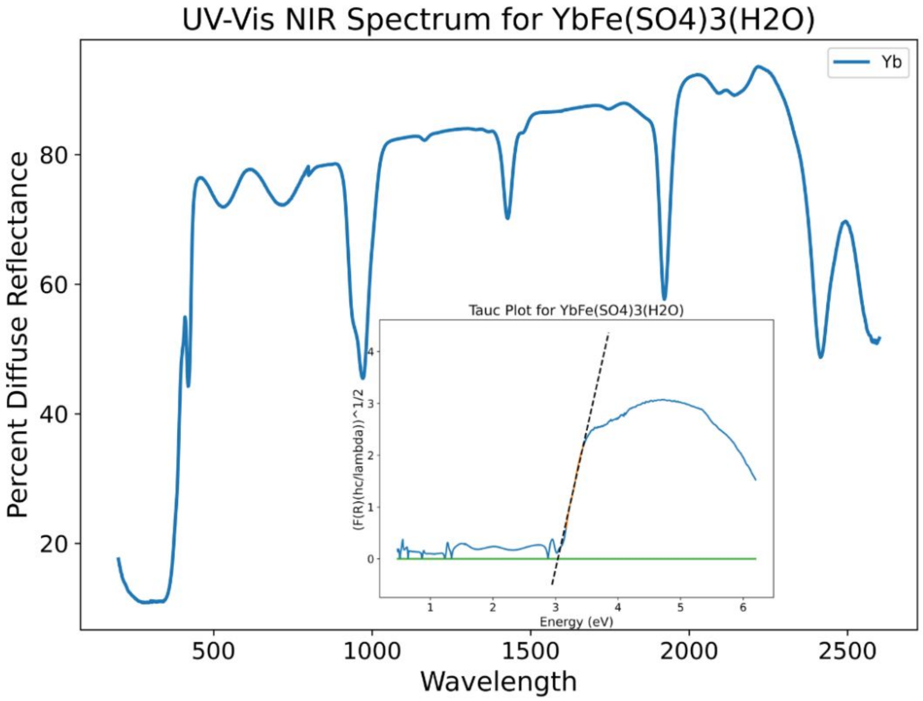

Figure S35: The UV-Vis NIR spectrum and Tauc plot for compound **13.**

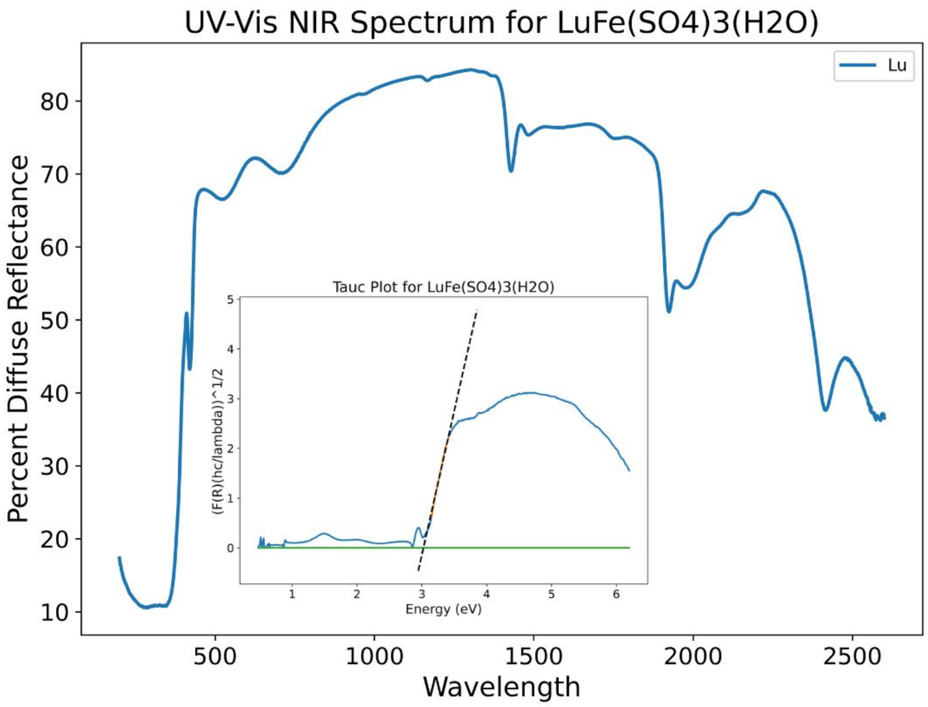

Figure S36: The UV-Vis NIR spectrum and Tauc plot for compound **14**.

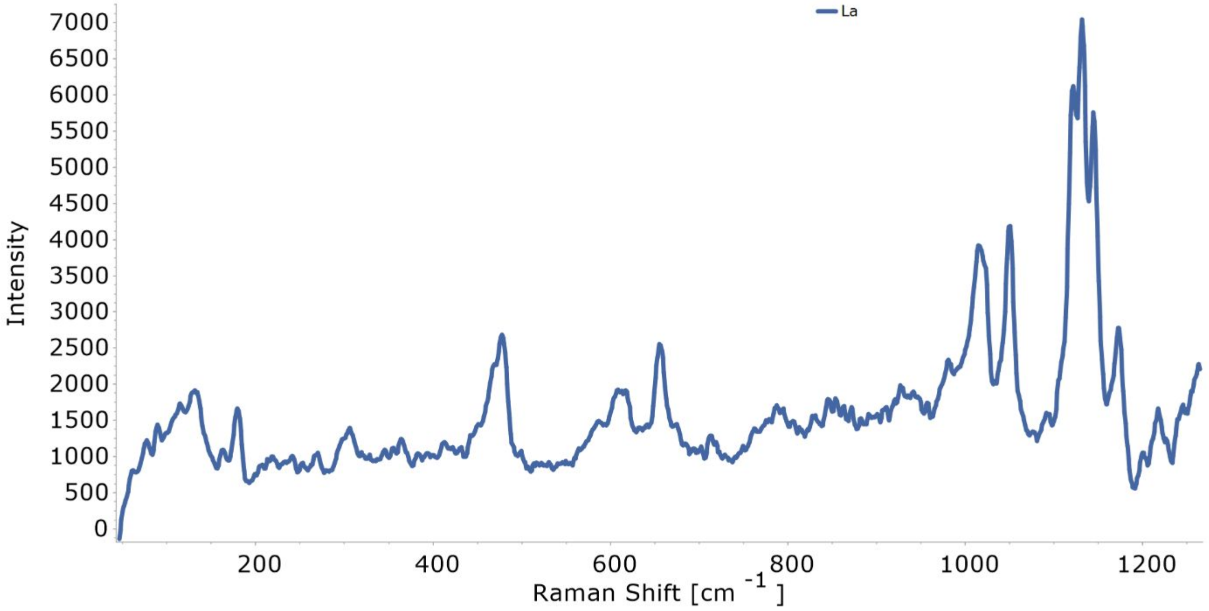

Figure S37: The Raman spectrum for compound **1.**

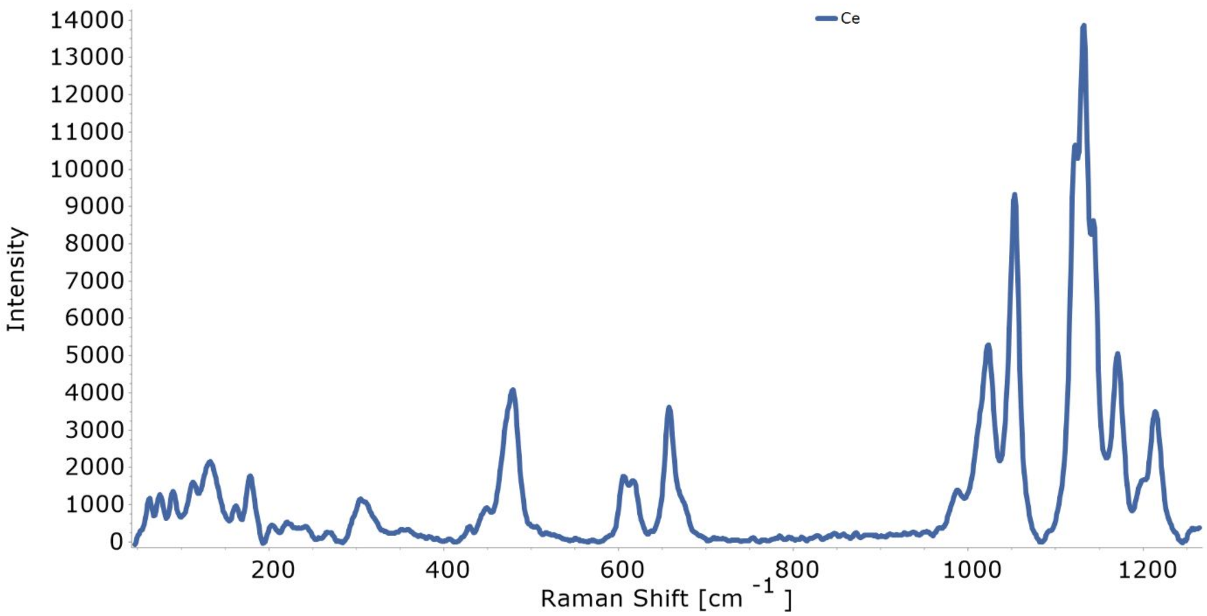

Figure S38: The Raman spectrum for compound **2.**

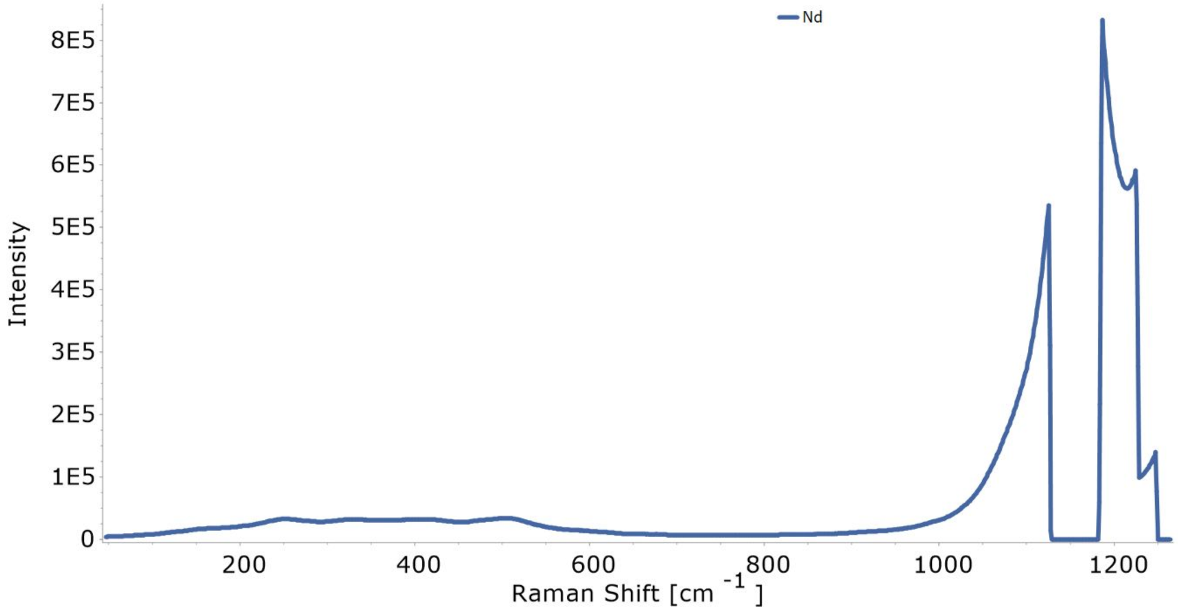

Figure S39: The Raman spectrum for compound **4.**

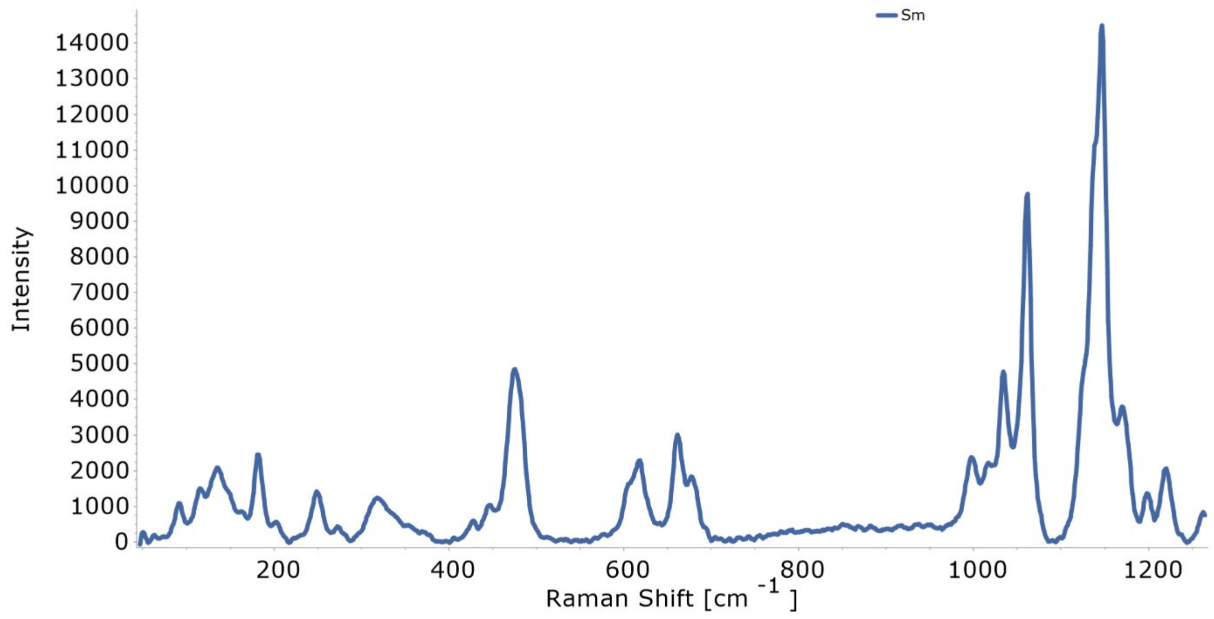

Figure S40: The Raman spectrum for compound **5.**

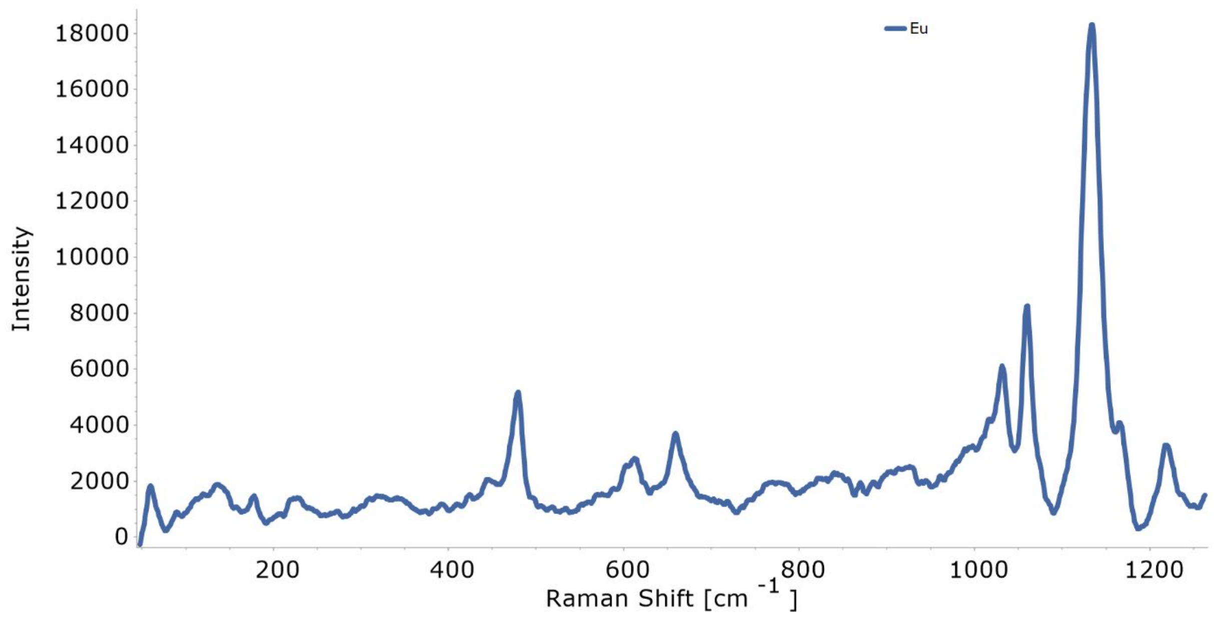

Figure S41: The Raman spectrum for compound **6.**

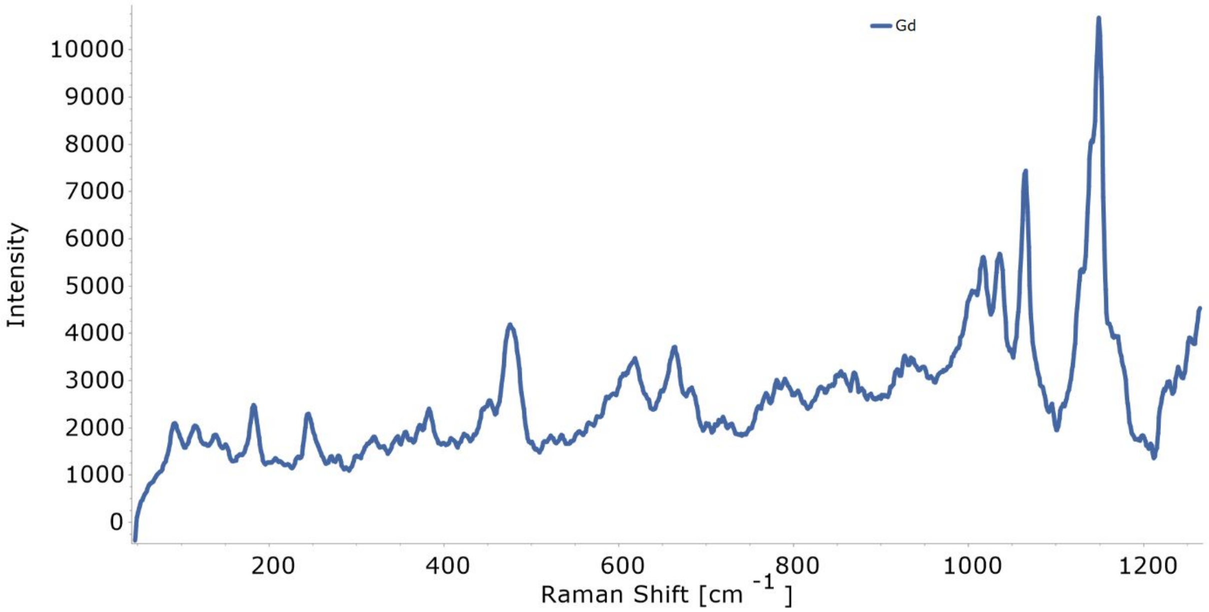

Figure S42: The Raman spectrum for compound **7.**

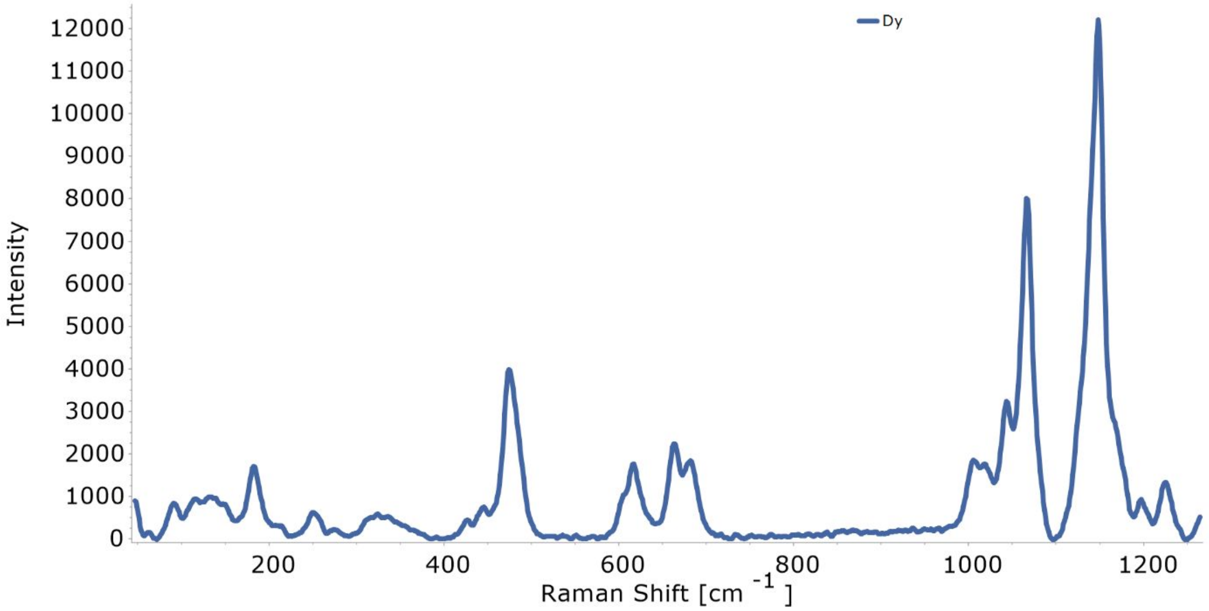

Figure S43: The Raman spectrum for compound **8.**

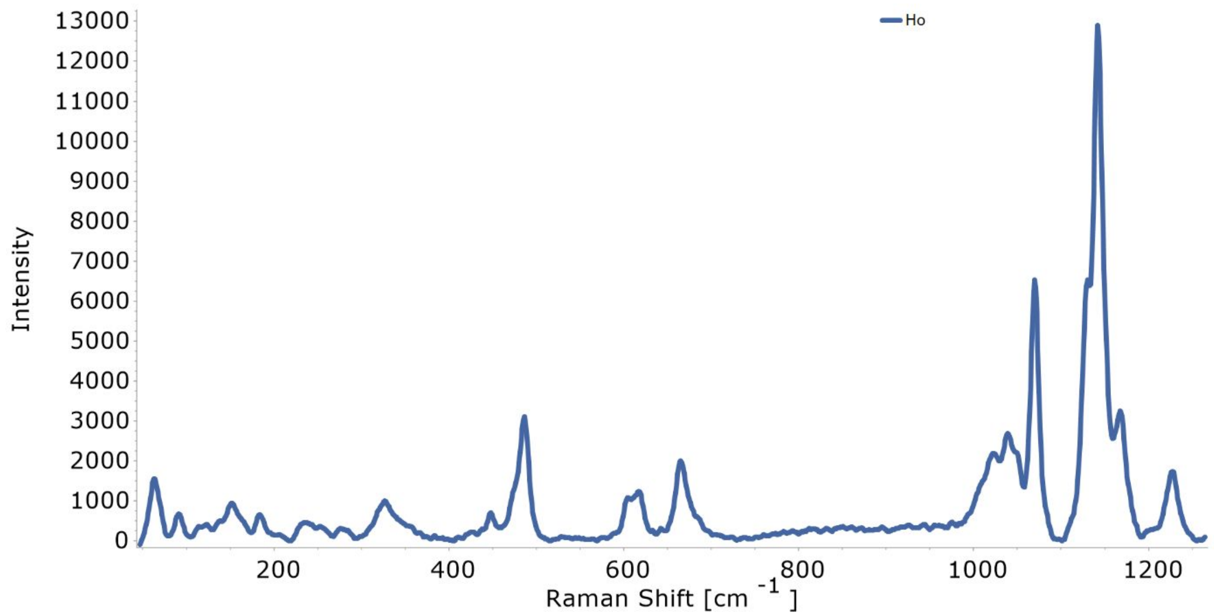

Figure S44: The Raman spectrum for compound **9.**

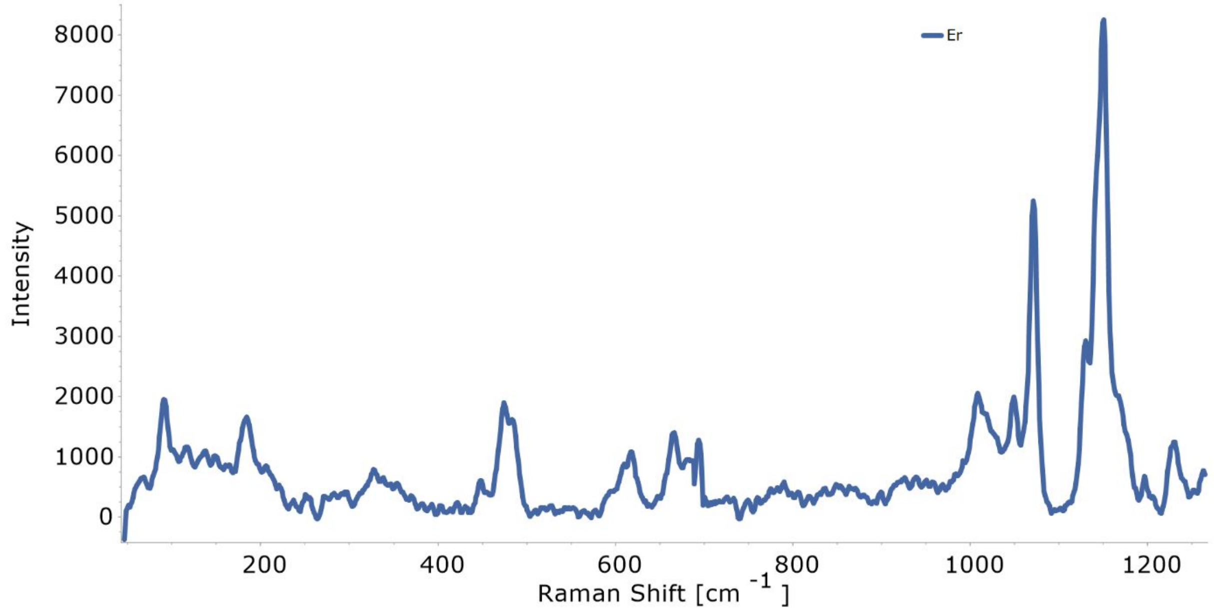

Figure S45: The Raman spectrum for compound **10.**

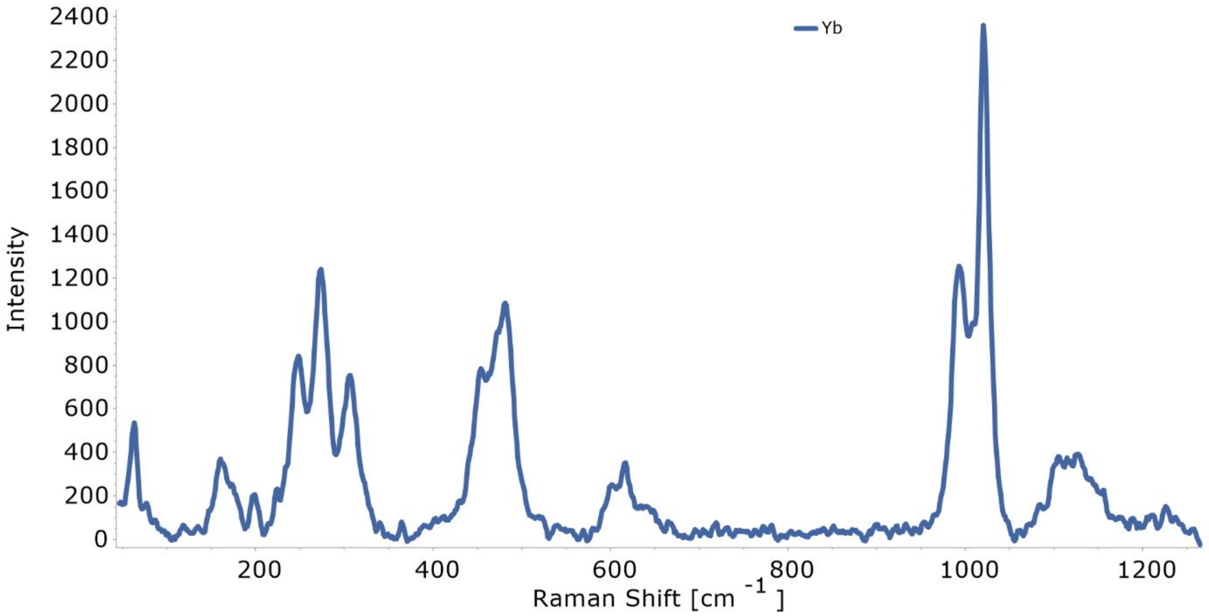

Figure S46: The Raman spectrum for compound **13.**

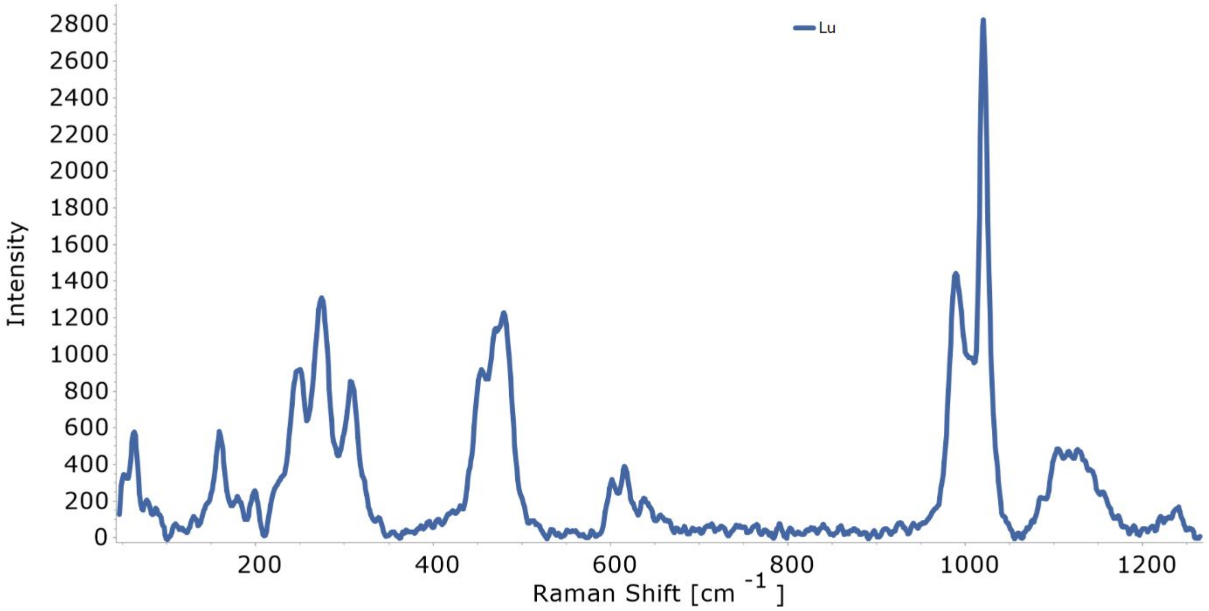

Figure S47: The Raman spectrum for compound **14.**

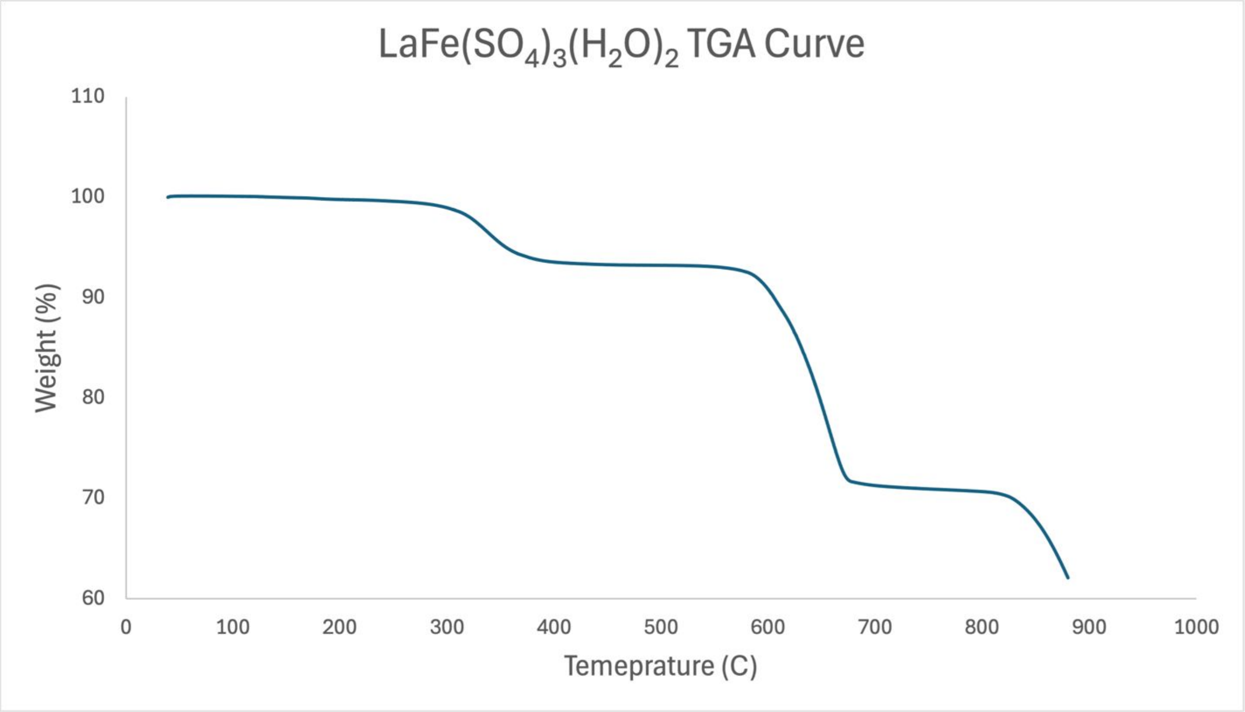

Figure S48: TGA curve for compound **1.**

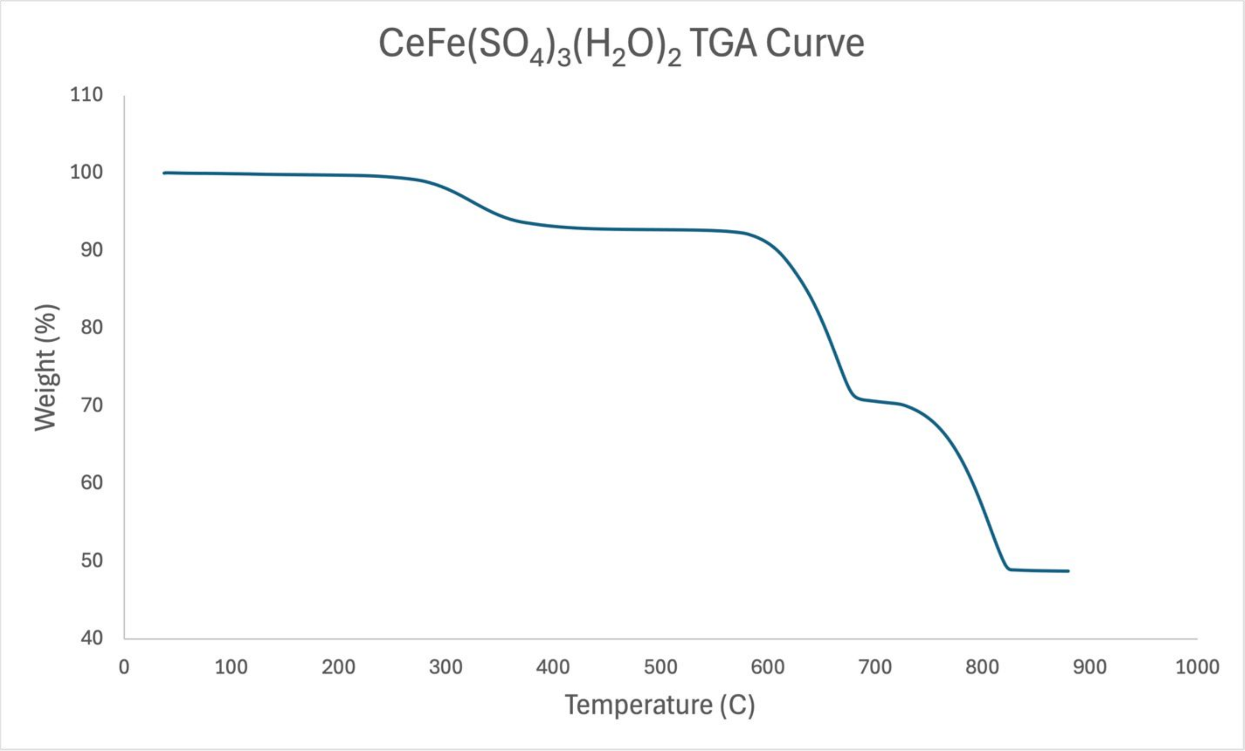

Figure S49: TGA curve for compound **2.**

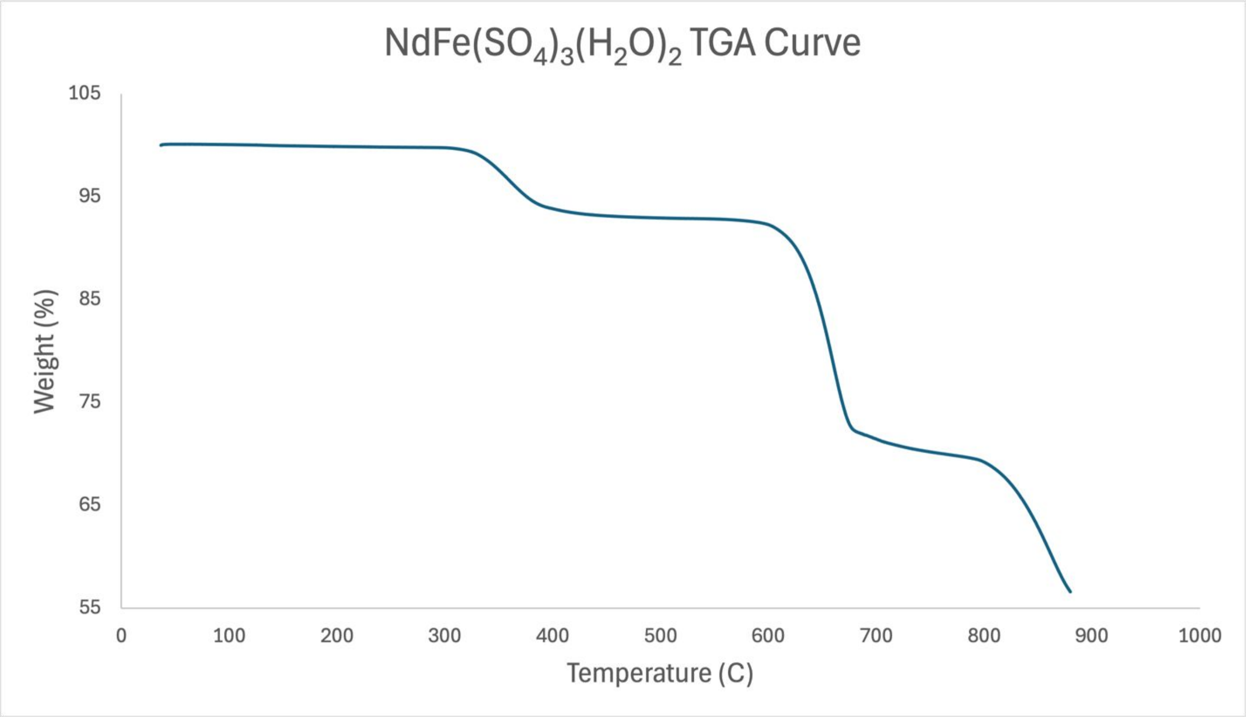

Figure S50: TGA curve for compound **4.**

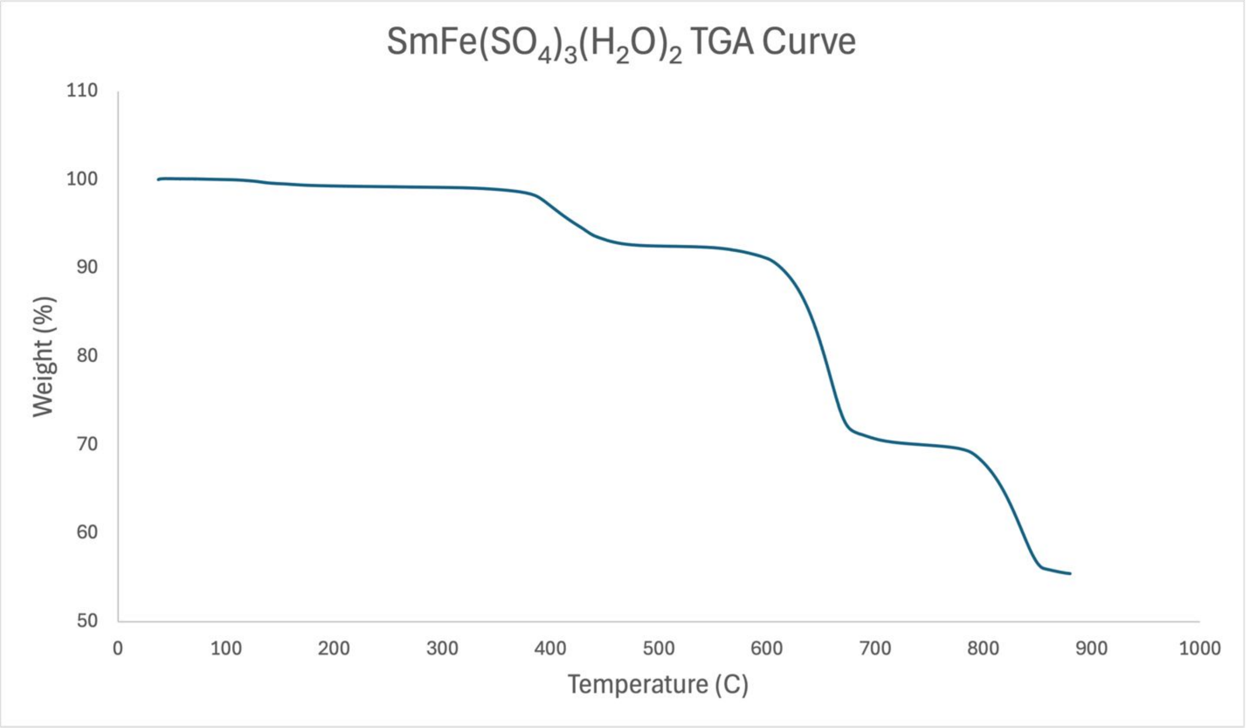

Figure S51: TGA curve for compound **5.**

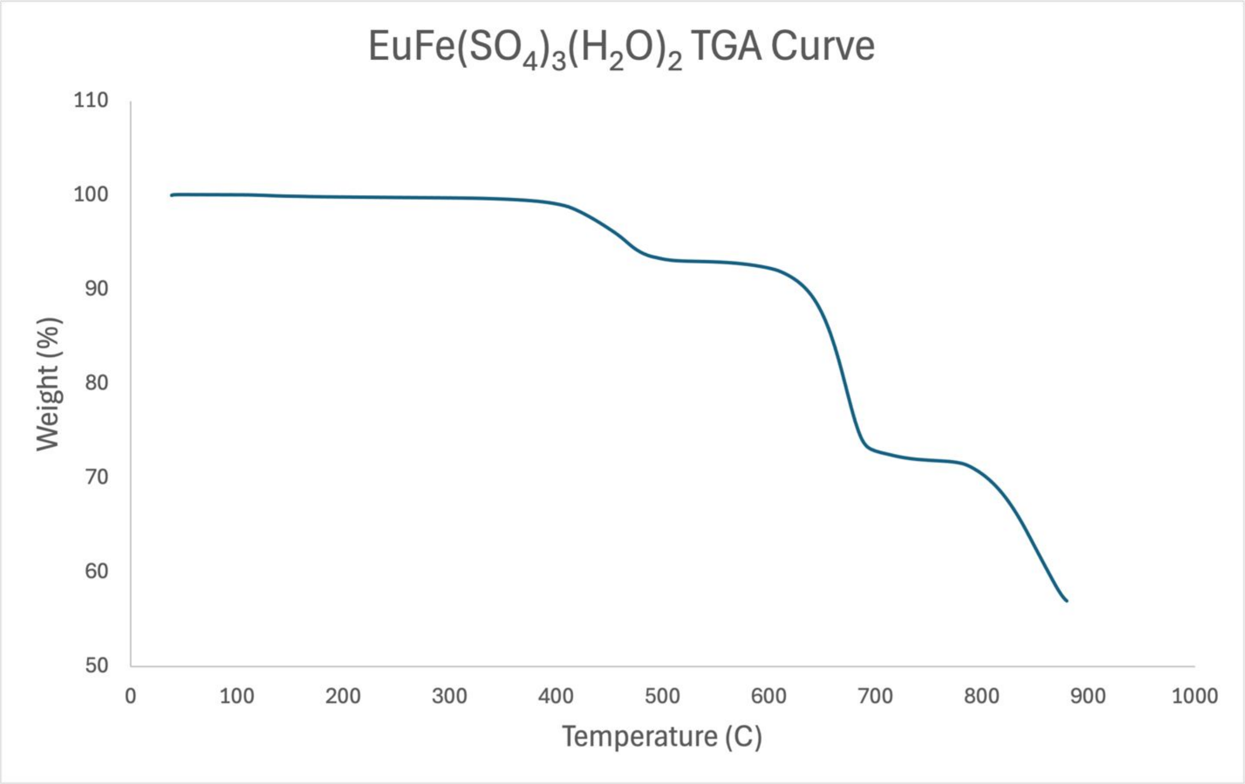

Figure S52: TGA curve for compound **6.**

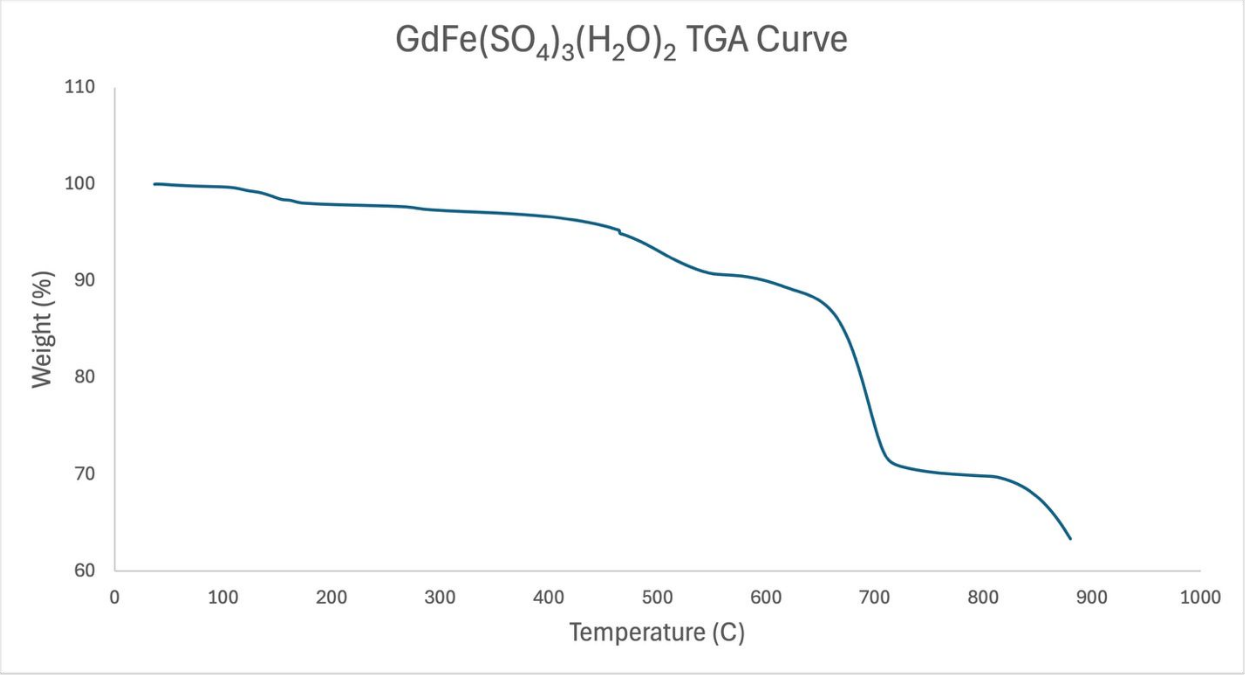

Figure S53: TGA curve for compound **7.**

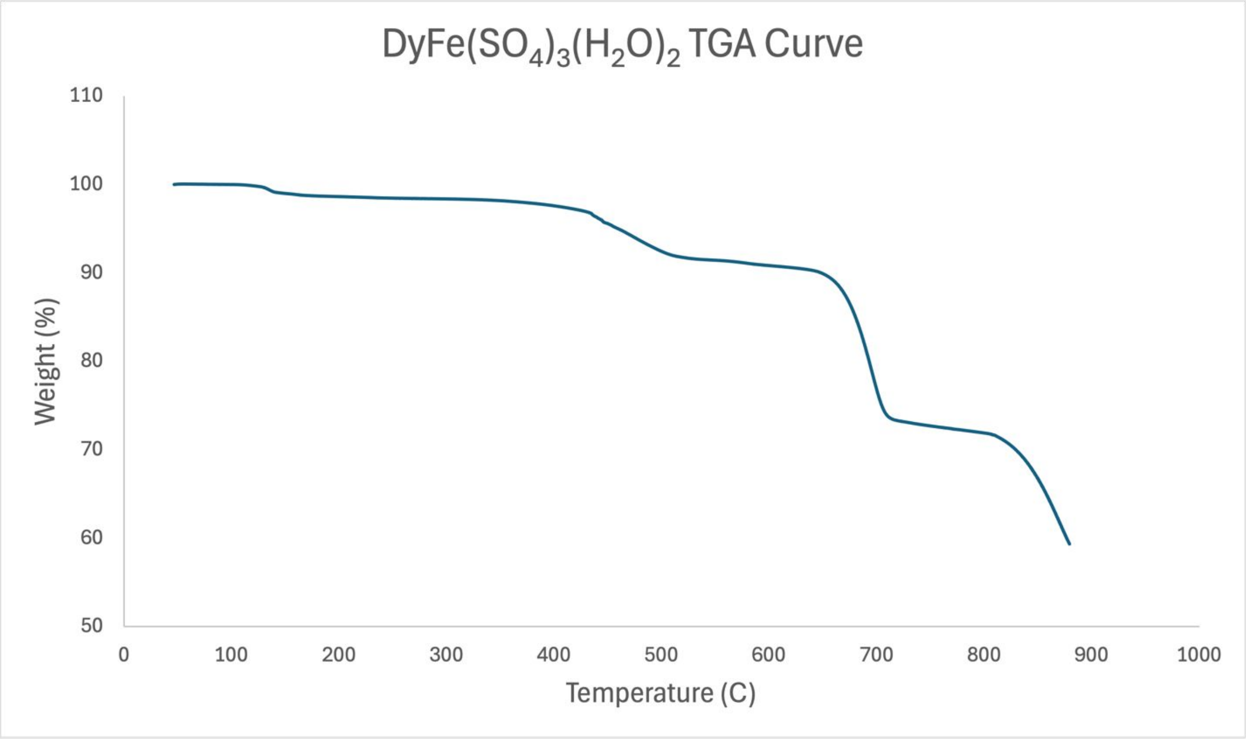

Figure S54: TGA curve for compound **8.**

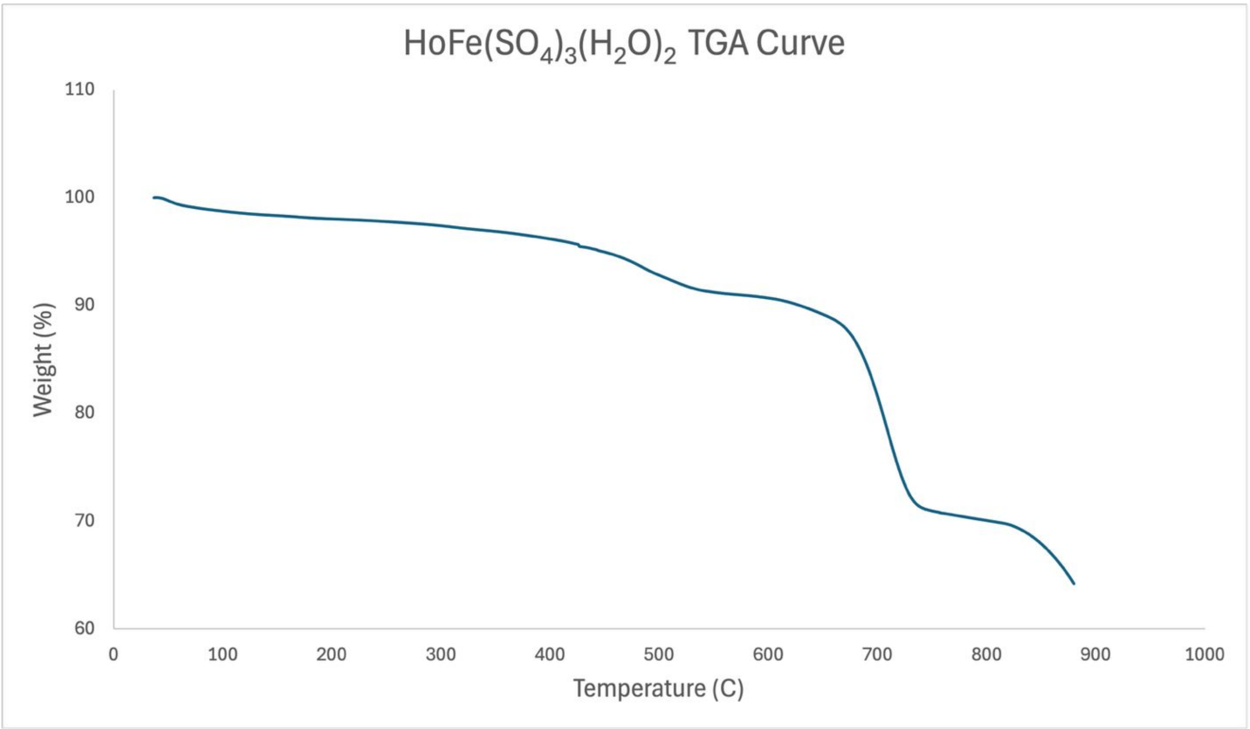

Figure S55: TGA curve for compound **9.**

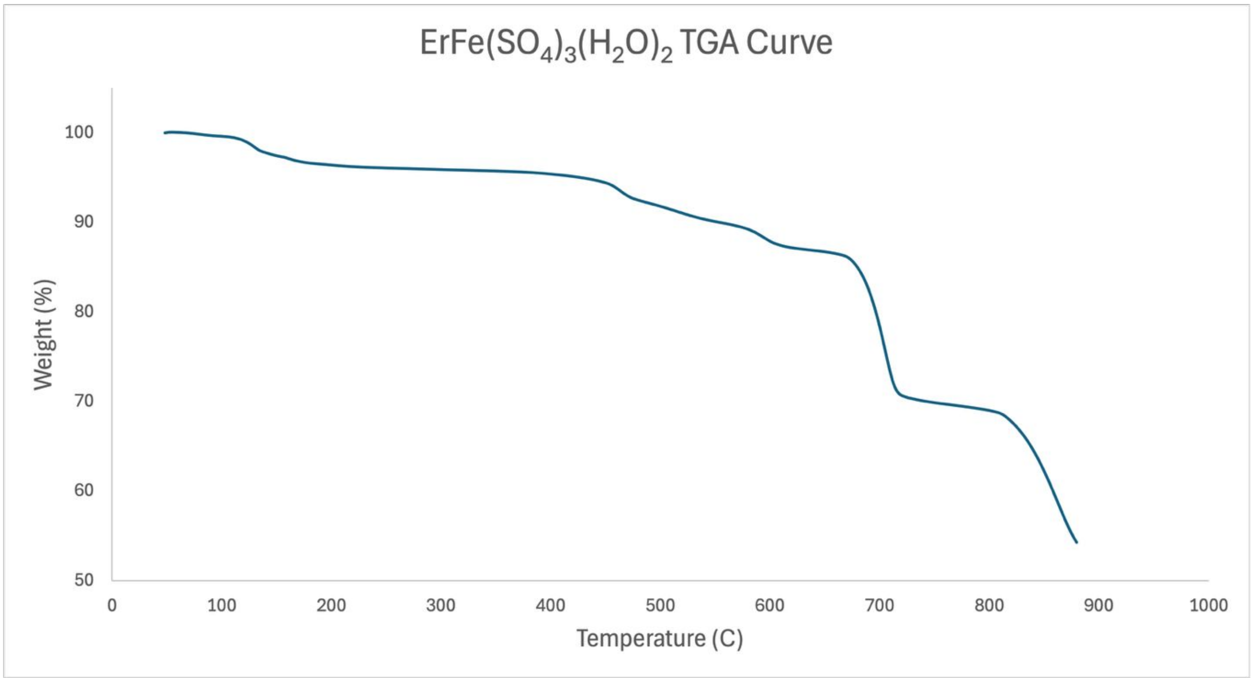

Figure S56: TGA curve for compound **10.**

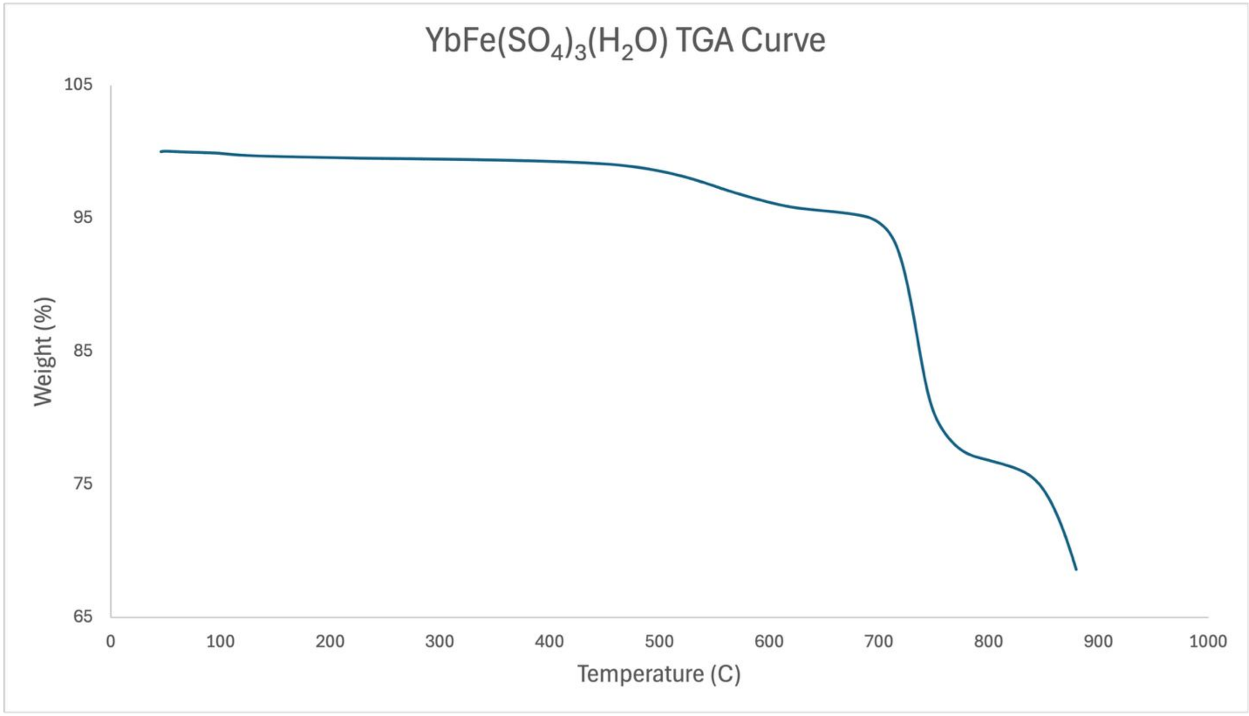

Figure S57: TGA curve for compound **13.**

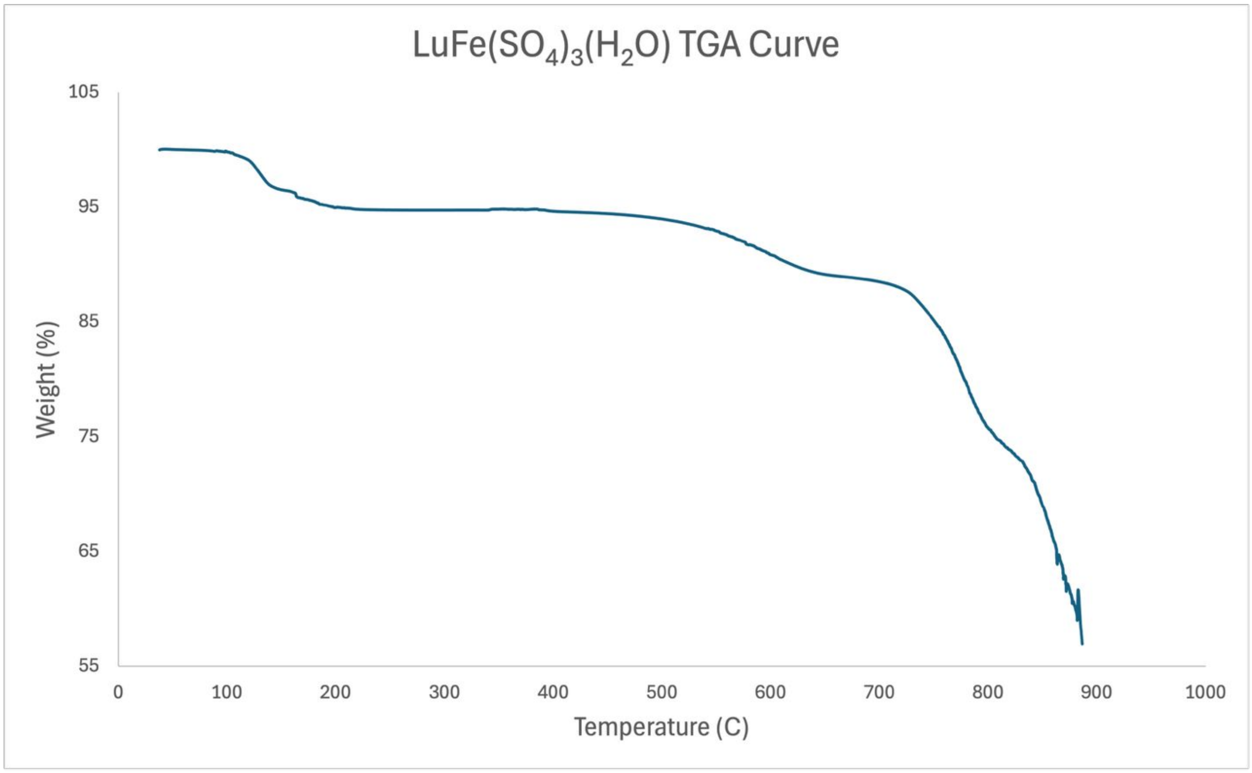

Figure S58: TGA curve for compound **14.**

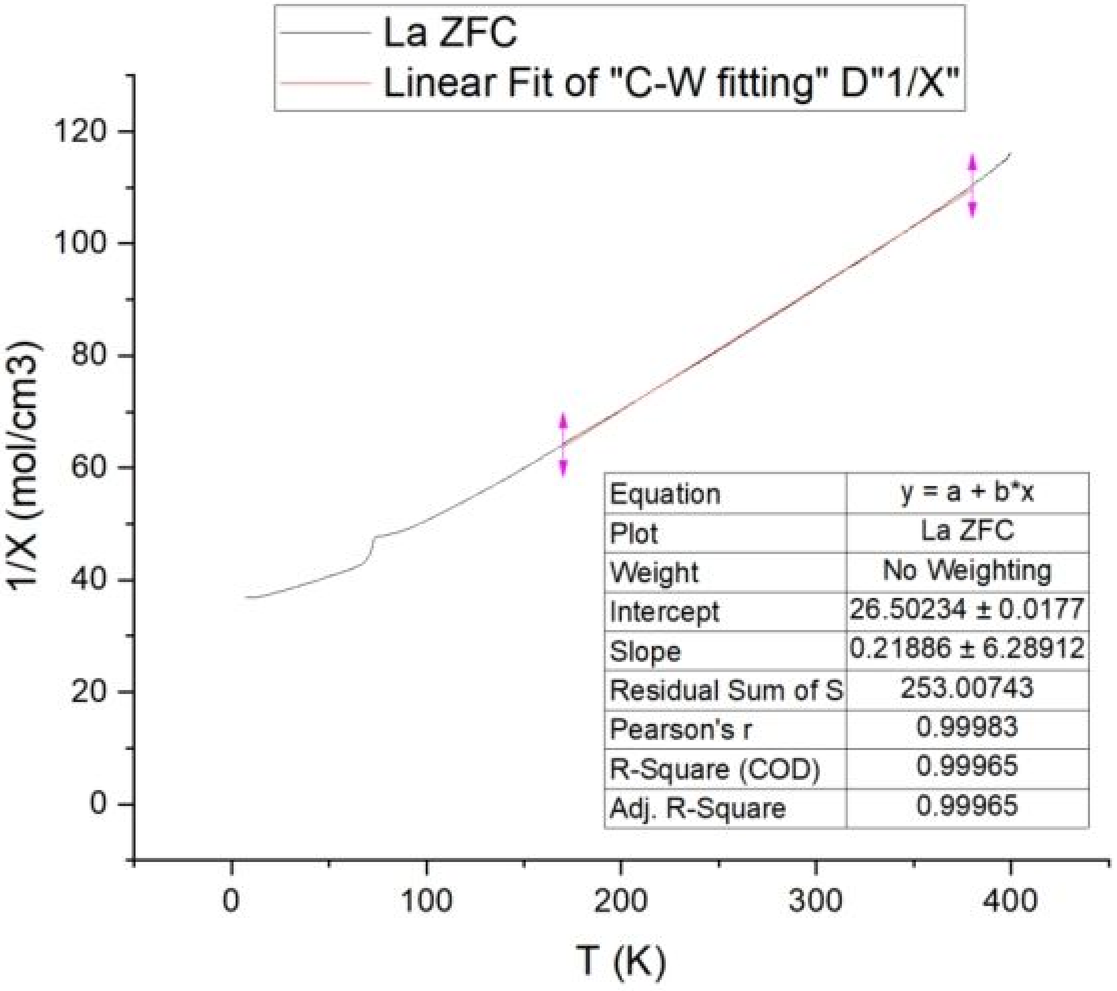

Figure S59: Temperature dependence of inverse magnetic susceptibility (1/χ) for compound **1.**

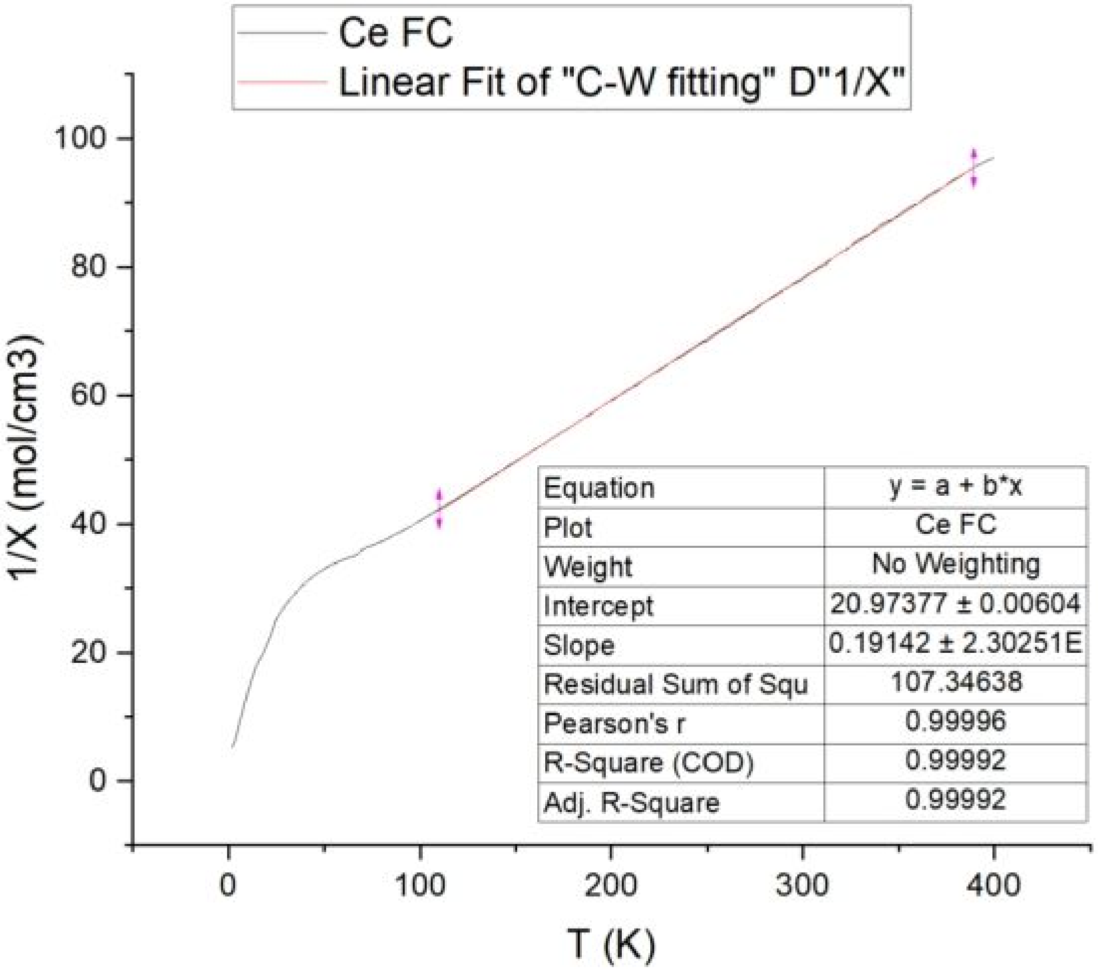

Figure S60: Temperature dependence of inverse magnetic susceptibility (1/χ) for compound **2.**

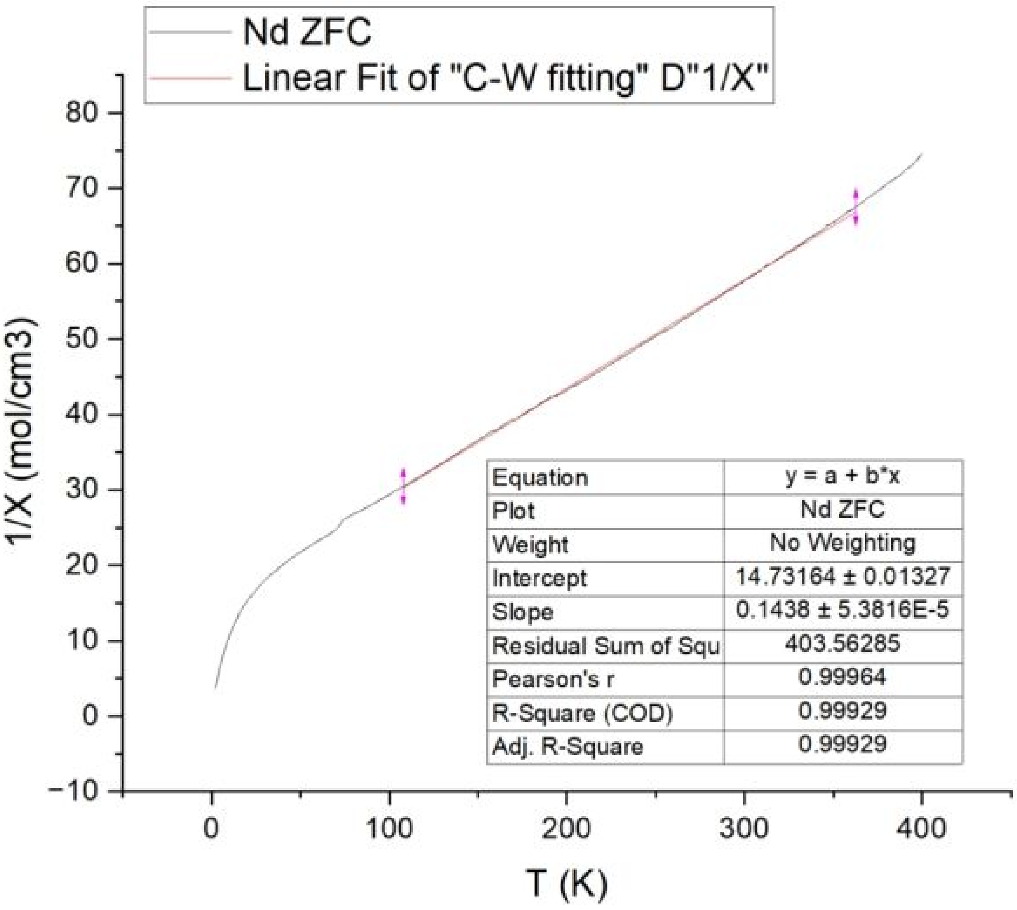

Figure S61: Temperature dependence of inverse magnetic susceptibility ($1/\chi$) for compound **4.**

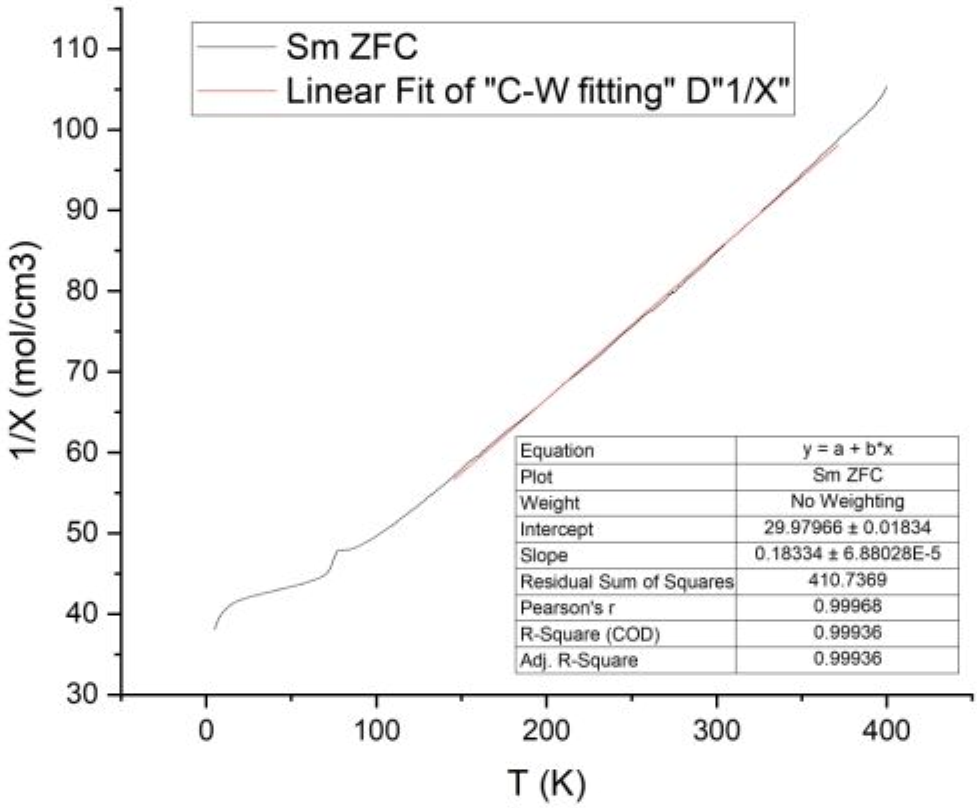

Figure S62: Temperature dependence of inverse magnetic susceptibility (1/χ) for compound **5.**

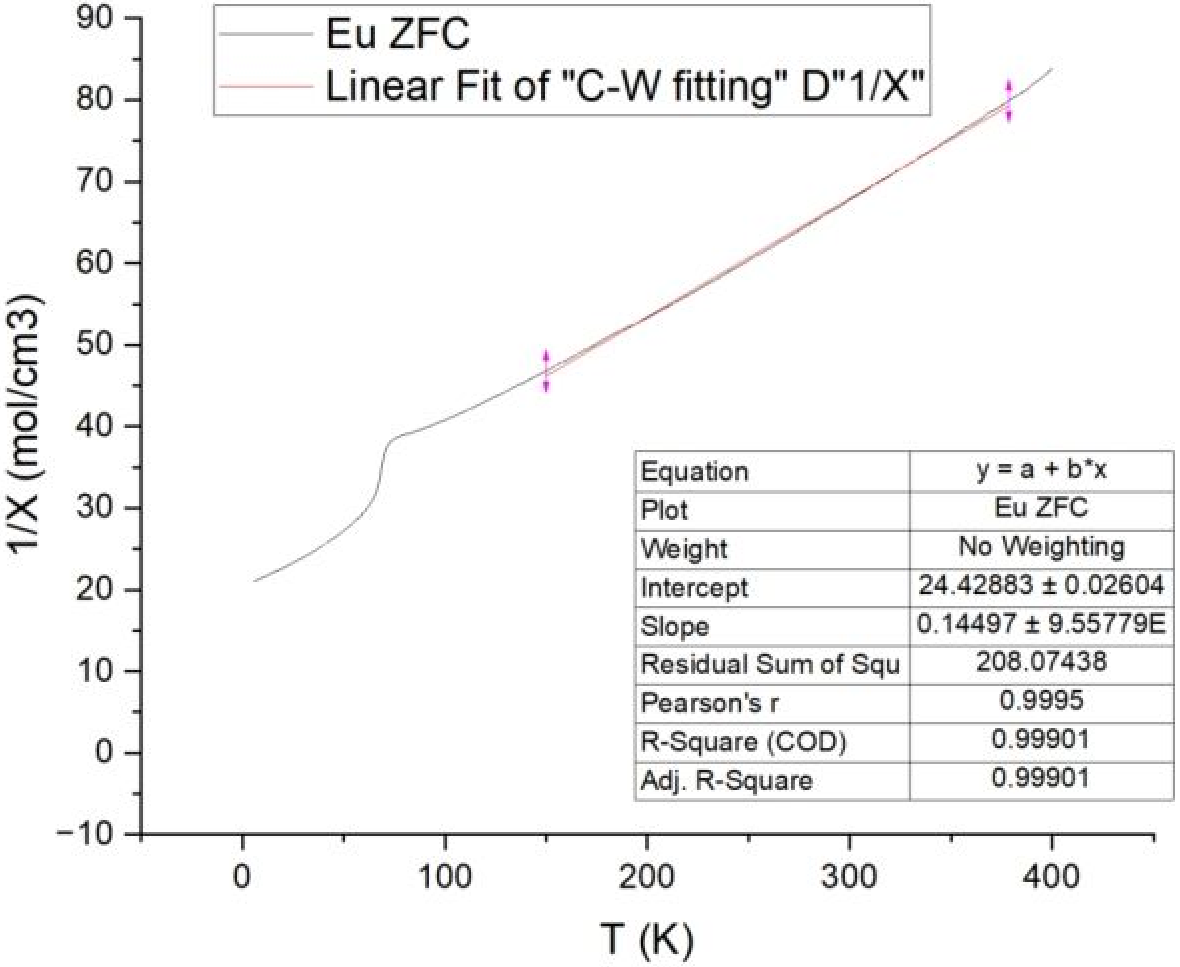

Figure S63: Temperature dependence of inverse magnetic susceptibility ($1/\chi$) for compound **6.**

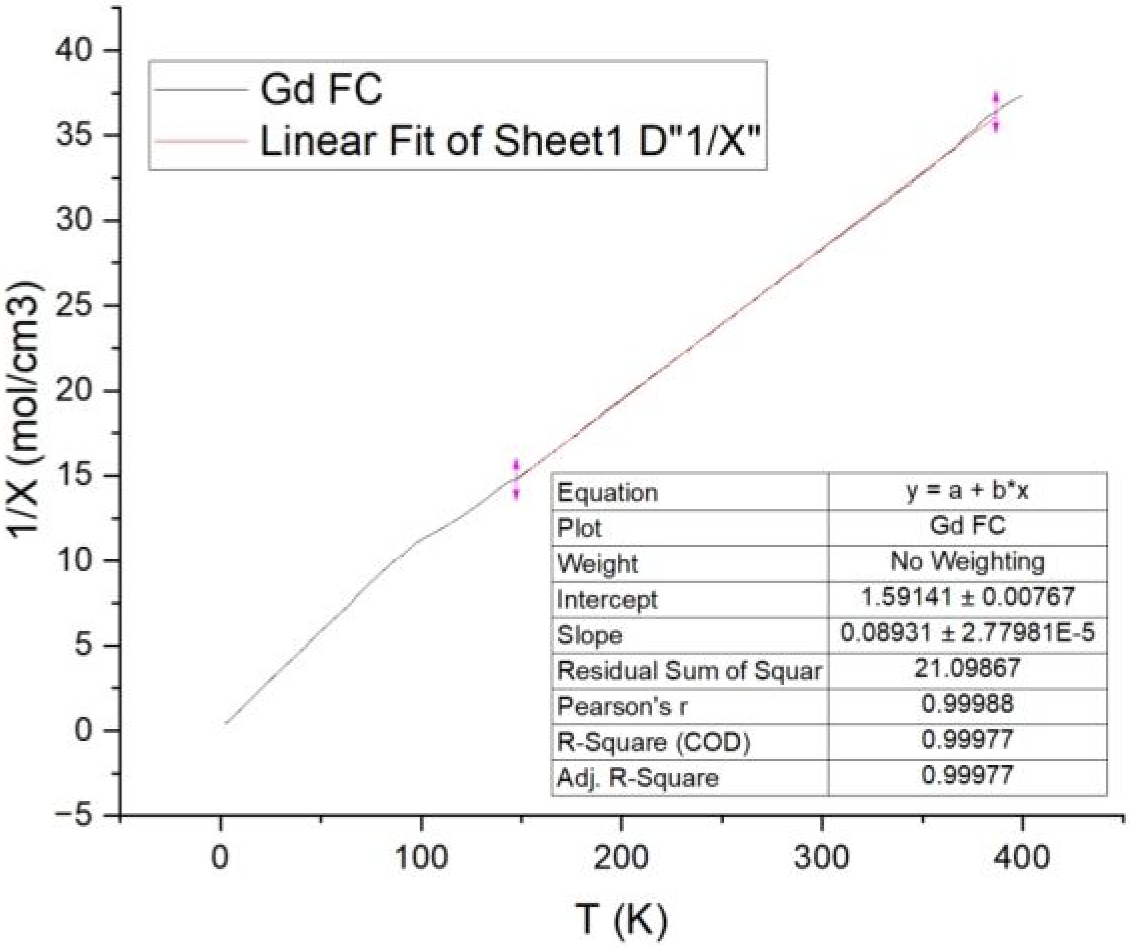

Figure S64: Temperature dependence of inverse magnetic susceptibility ($1/\chi$) for compound **7.**

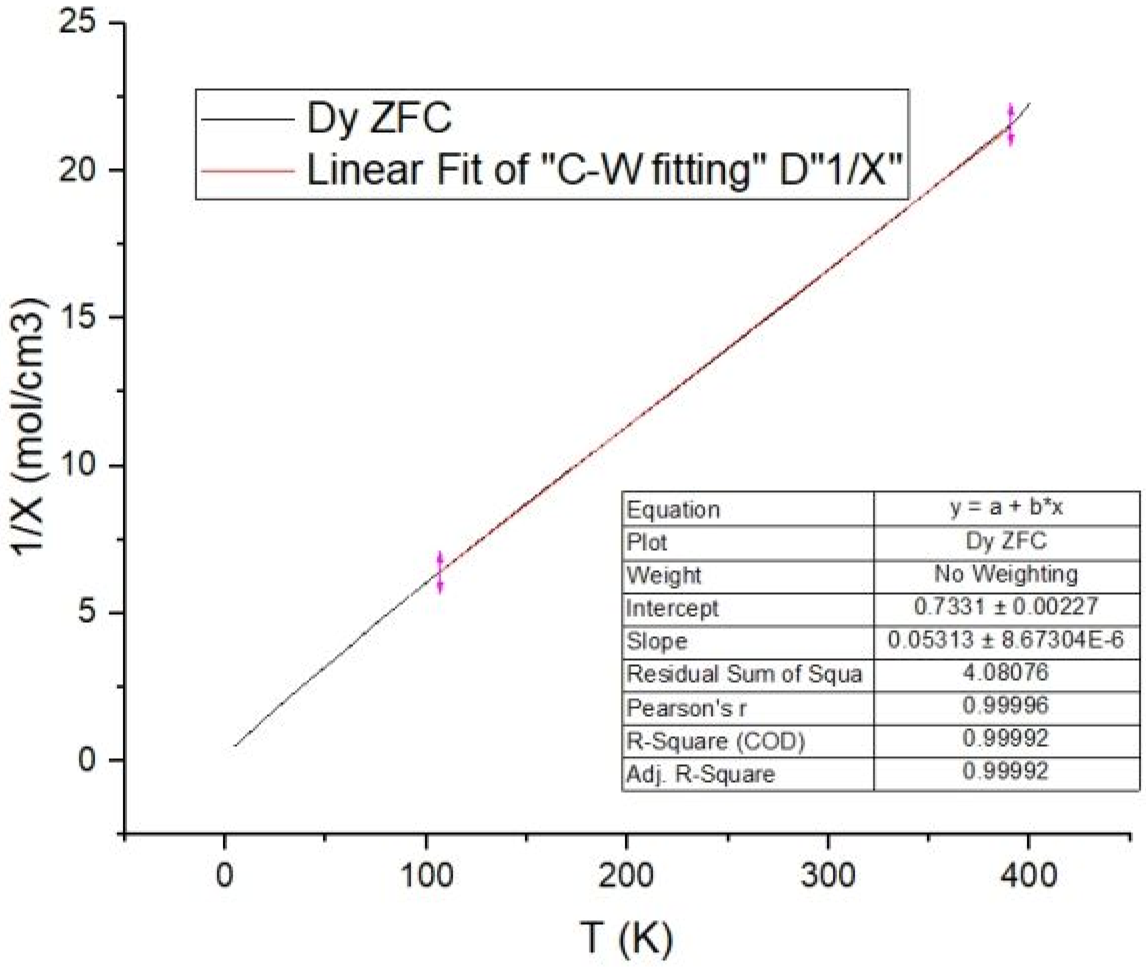

Figure S65: Temperature dependence of inverse magnetic susceptibility (1/χ) for compound **8.**

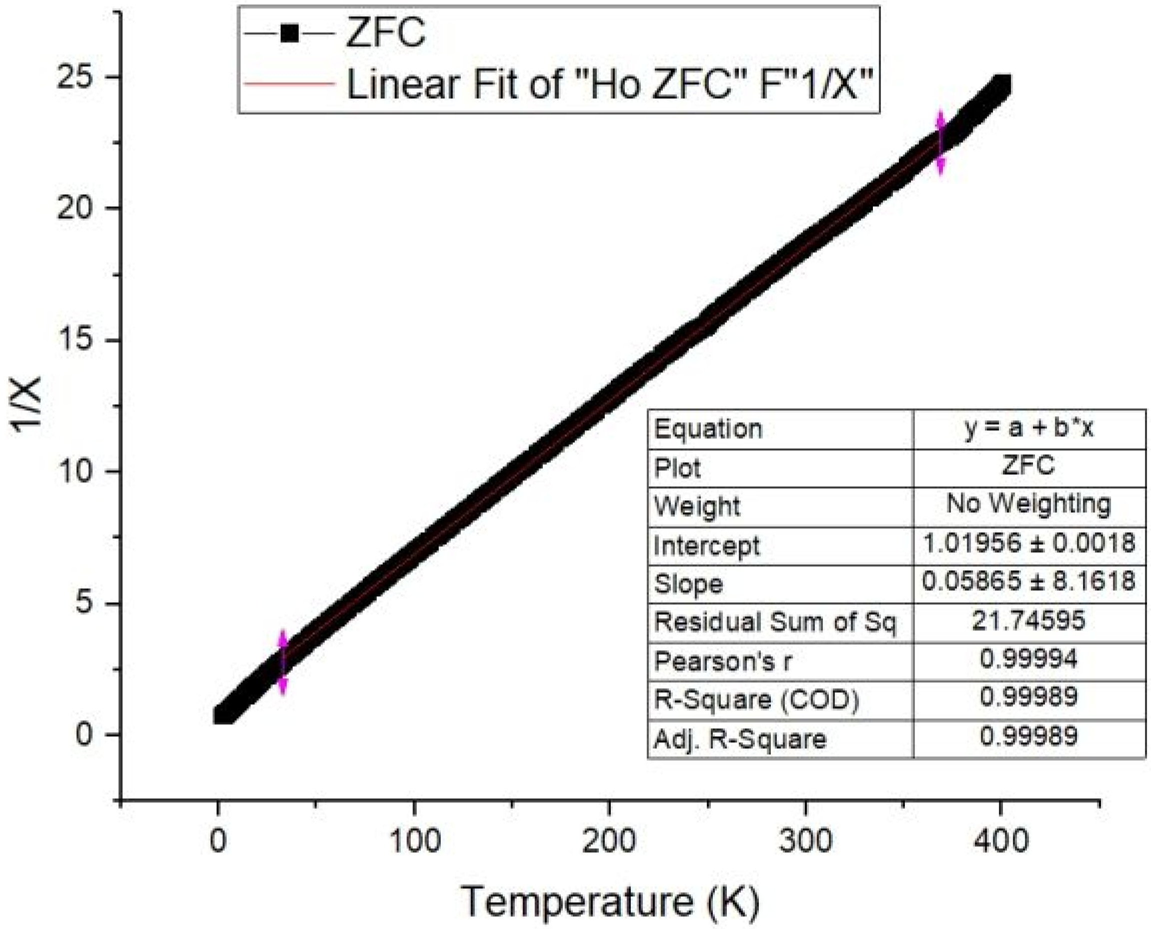

Figure S66: Temperature dependence of inverse magnetic susceptibility ($1/\chi$) for compound **9.**

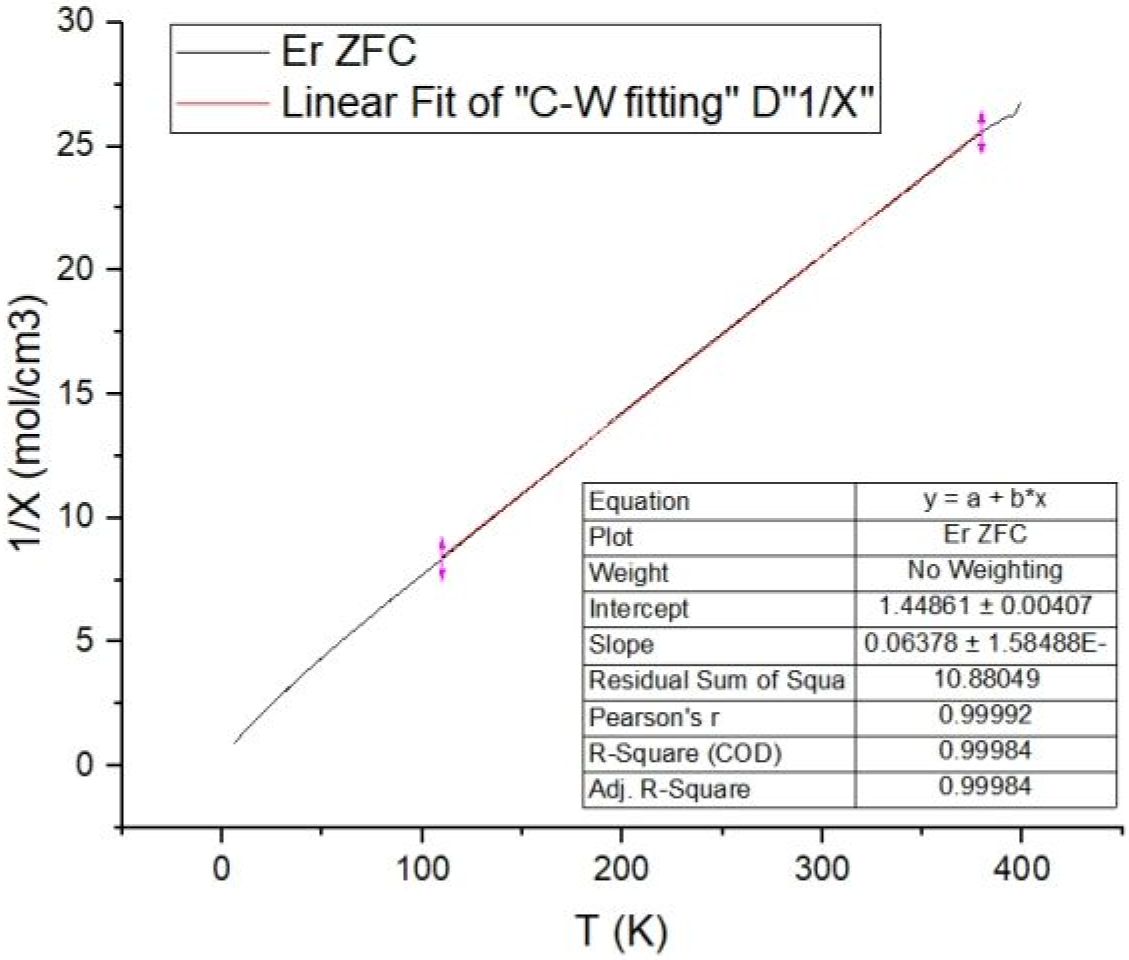

Figure S67: Temperature dependence of inverse magnetic susceptibility (1/χ) for compound **10.**

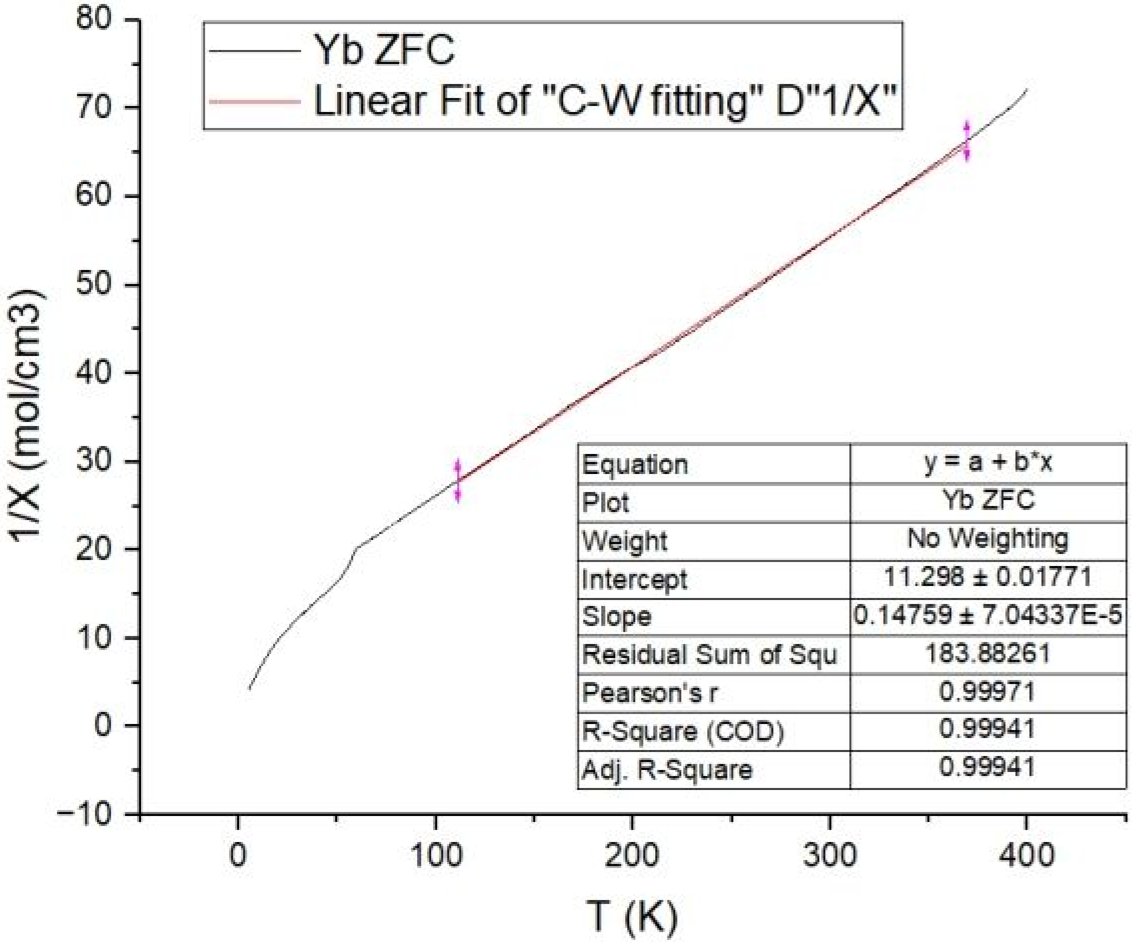

Figure S68: Temperature dependence of inverse magnetic susceptibility ($1/\chi$) for compound **13.**

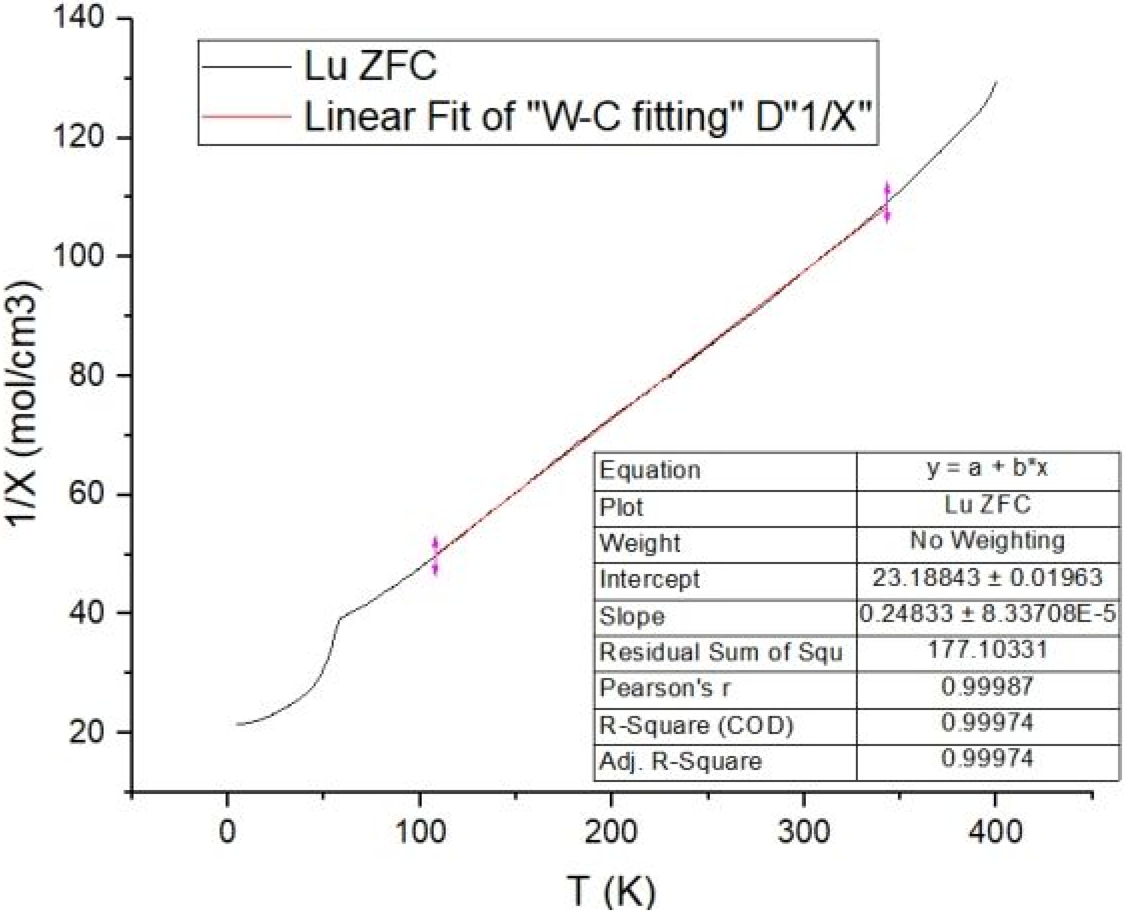

Figure S69: Temperature dependence of inverse magnetic susceptibility (1/χ) for compound **14.**
